# Implementing HIV pre-exposure prophylaxis (PrEP) among cisgender female sex workers: A worldwide scoping review to guide the future research agenda for HIV prevention

**DOI:** 10.1371/journal.pgph.0007582

**Published:** 2026-11-13

**Authors:** Mélanie Plazy, Valentine Becquet, Simon Amador-Paz, Marcellin Nouaman, Esther Maouhoub, Carla Meertens, Remi Mouquin, Hasna Youssoufa, Patrick Coffie, Joseph Larmarange

**Affiliations:** 1 University of Bordeaux, INSERM UMR 1219, IRD EMR 271, Bordeaux Population Health Research Center, Bordeaux, France; 2 ISPED, Bordeaux School of Public Health, University of Bordeaux, Bordeaux, France; 3 Ined, Aubervilliers, France; 4 Ceped, Université Paris-Cité, Université Sorbonne Paris Nord, IRD, Inserm, Paris, France; 5 Inserm – ANRS MIE, Paris, France; 6 PAC-CI, Abidjan, Côte d’Ivoire; 7 Département de Santé Publique, UFR d’Odonto-Stomatologie, Université Félix Houphouet-Boigny, Abidjan, Côte d’Ivoire; 8 Département de Dermatologie et Infectiologie, UFR des Sciences Médicales, Université Félix Houphouet-Boigny, Abidjan, Côte d’Ivoire; The University of Sydney, AUSTRALIA

## Abstract

Female sex workers (FSWs) experience a disproportionately high burden of HIV. To inform future research and policy efforts on HIV prevention, this study synthesizes global evidence on HIV pre-exposure prophylaxis (PrEP) among cisgender FSWs worldwide. This scoping review was conducted using six multidisciplinary databases searched up to December 2024. Titles and abstracts, then full texts, were independently screened. Data extraction comprised i) general study characteristics (UNAIDS region, country, study type, outcomes, type of PrEP, and study years), ii) detailed data of each outcome (including PrEP knowledge, a priori acceptability – interest, and effective acceptability – use, retention and adherence). Drivers and barriers to PrEP acceptability were analyzed using an adapted conceptual framework encompassing six domains: patient characteristics, individual knowledge and perceptions, medication-related factors, social processes, structural and health system factors, and sex work characteristics. After selection, 120 eligible studies were identified, mostly conducted in Eastern and Southern Africa. PrEP knowledge was initially low but increased quickly once programs were introduced. PrEP interest was consistently high across regions, driven by perceived HIV risk and desire for self‑protection. PrEP uptake, retention and adherence varied widely. Main barriers included sex work realities (i.e., unstable housing, alcohol use), social processes (i.e., stigma), individual characteristics (i.e., mobility), treatment-related characteristics (i.e., side effects), and health system challenges (i.e., clinic access constraints). Drivers included social support and reminder tools. While evidence on long‑acting PrEP remained limited, interest was consistently high as reducing daily burden. Overall, the findings support a socio-ecological understanding of PrEP engagement among FSWs, including the sex work context as a distinct intermediary layer. They highlight the need for differentiated and structurally responsive PrEP delivery models tailored to the realities of sex work. Expanding long‑acting PrEP options and improving service accessibility, community engagement, and stigma reduction strategies will be critical to strengthening HIV prevention among FSWs globally.

## Introduction

Female sex workers (FSWs) are among the populations most at risk of HIV infection. In 2022, the relative risk of acquiring HIV was globally nine times higher for sex workers than in the general adult population [1]. While consistent condom use remains a key prevention strategy, the vulnerability of FSWs to HIV is driven by multiple factors [2,3]: it is not always possible for FSWs to negotiate condom use due to client refusal and coercion, including gender-based violence and sexual violence, particularly in the context of alcohol use. This vulnerability is further exacerbated by economic dependency and power imbalances in sexual transactions. Additionally, because FSWs engage in frequent sexual intercourse with multiple partners, they face an increased risk of condom failure or breakage.

To address these challenges, oral daily pre-exposure prophylaxis (PrEP) has been recommended by the World Health Organization (WHO) since 2015 for individuals at substantial risk of HIV acquisition, including FSWs [4]. In recent years, new long-acting PrEP modalities have been developed and recommended by the WHO to expand prevention options: the dapivirine vaginal ring for women in 2021 [5], followed by the injectable Cabotegravir (CAB), which protects for two months, in 2022 [6] and the injectable Lenacapavir, designed to last for six months, in 2025 [7]. Other PrEP formulations are currently being tested in various phases of clinical trials, including islatravir implants, vaginal rings [8], and gel-based PrEP. These new methods could offer FSWs more flexible and discreet prevention options, potentially improving uptake and adherence.

Two previous literature reviews have been conducted on oral PrEP use among female sex workers: one included studies published up to the end of 2018 [9], while the other focused on Africa [10]. No recent comprehensive synthesis of PrEP research among cisgender FSWs worldwide is currently available. To inform future research and policy efforts, this review therefore aims to fill this gap by synthesizing evidence of PrEP implementation among cisgender FSWs worldwide. Specifically, it examines PrEP knowledge, *a priori* and effective PrEP acceptability, socio-behavioral impacts of PrEP use, as well as identifies key drivers and barriers of PrEP implementation in FSWs.

## Methods

### Search strategy

We have conducted a scoping literature review following the Preferred Reporting Items for Systematic Reviews and Meta-Analysis extension for Scoping Reviews (PRISMA-ScR) guidelines [11] (S1 Table). Six databases covering medical and social sciences literature were used: Pubmed, Scopus, SocINDEX, APA PsycArticles, APA PsycInfo, Psychology and Behavioral Sciences Collection. The search strategy combined the following terms, searched using database-specific Title and Abstract fields: (“PrEP” OR “preexposure prophylaxis” OR “pre-exposure prophylaxis”) AND (“HIV” OR “AIDS”) AND “sex-work*” (S1 Fig). The search included records published up to 31 December 2024.

### Eligibility criteria and studies’ selection

Inclusion and exclusion criteria are presented in Table 1. All manuscripts on PrEP among cisgender women who identified as FSWs were included worldwide, regardless of the type of PrEP, the study design, or the type of data collection. We focused specifically on cisgender women to maintain a clear and sufficiently homogeneous scope, as the social, structural, and healthcare contexts influencing PrEP engagement may differ from those of transgender women and other gender-diverse populations. More specifically, papers were excluded if: (1) they did not present specific or stratified results on cisgender FSWs; (2) they did not focus on PrEP; (3) they focused exclusively on biological aspects of PrEP; (4) they were not original research or were modeling studies; (5) they were not written in English or French; (6) full text was not available. Data screening was conducted on the online PICO Portal software, by a large team composed of research assistants (EM, CM, RM, HY for studies published until Dec 2022, and SAP for studies published in 2023–2024) and senior researchers (MP, VB, JL). After the removal of duplicate references, each title and abstract was independently screened by three reviewers (two research assistants and one senior researcher). The full texts of selected abstracts were then independently read by two senior researchers for final inclusion. At each of these selection steps, disagreements were resolved through discussion with a third senior researcher.

**Table 1:** Inclusion and exclusion criteria. Scoping review on PrEP among female sex workers.

	Inclusion Criteria	Exclusion Criteria
**1. Population**	- Female cisgender sex workers Worldwide	- Men - Transgender - Transactional sex (e.g., sugar daddies) - Staff/ healthcare professionals only
**2. Intervention**	- All types of PrEP (*oral, implant, injection, vaginal douche, vaginal rings, gel-based*)	- No data on PrEP
**3. Outcomes**	- Knowledge - A priori acceptability (*willingness/interest*) - Effective acceptability (*uptake/use, retention, adherence*) - Impacts *(social, epidemiological, behavioral*) - Efficacy (*on HIV incidence)*, Cost-efficacy - Costs - PrEP delivery/ Preparation of the intervention	- Biological development
**5. Paper types**	- Original papers other than modeling studies	- Protocols - Pre-print - Modeling studies - Editorial/opinion papers - Literature reviews - Conference abstracts - Reports that did not clearly present the following sections: Introduction, Methods, Results, Discussion
**6. Languages**	- French or English	- Other than French or English

### Data extraction

Data extraction was organized in two steps. First, to provide a global cartography of available data on PrEP among FSWs, each included paper was independently reviewed by two research assistants (EM, CM, RM, SAP) to extract the following information using the PICO Portal software: UNAIDS region, country, study type, outcomes, type of PrEP, and study years. Any discrepancies were resolved with one senior researcher (MP, VB or JL). Secondly, a senior researcher (MP) with the help of a junior researcher (SAP) extracted specific results focusing on each of the following outcomes using standardized Word tables: i) PrEP knowledge (S2 Table), ii) a priori PrEP acceptability - willingness or interest (S3 Table), iii) effective PrEP acceptability - use/uptake, retention, or adherence (S4 Table), iv) other outcomes of PrEP use (S5 Table). A coding system was used to classify findings as barriers or drivers (facilitators) and according to whether they were derived from quantitative or qualitative data. Standardized data extraction tools (on PICO Portal software and on Word tables) were jointly developed by the three senior researchers (MP, VB, JL).

### Data analysis

A conceptual framework was first developed based on two previous conceptual frameworks on medication use and adherence [12,13]. Our conceptual framework includes five relevant domains derived from these frameworks, related to i) patients’ characteristics, ii) individual knowledge and perceptions, iii) medication, iv) social processes, v) structural and health system characteristics. We added a sixth domain related to sex work characteristics. After data extraction, all drivers and barriers to a priori and effective acceptability of PrEP were analyzed and categorized within this conceptual framework.

## Results

### Study selection

Among the 615 published references identified through the search equation after duplicate removal, 273 were excluded based on the title and abstract, and 222 were excluded after full-text reading (Fig 1). Overall, 120 original papers were included.

**Fig 1:**
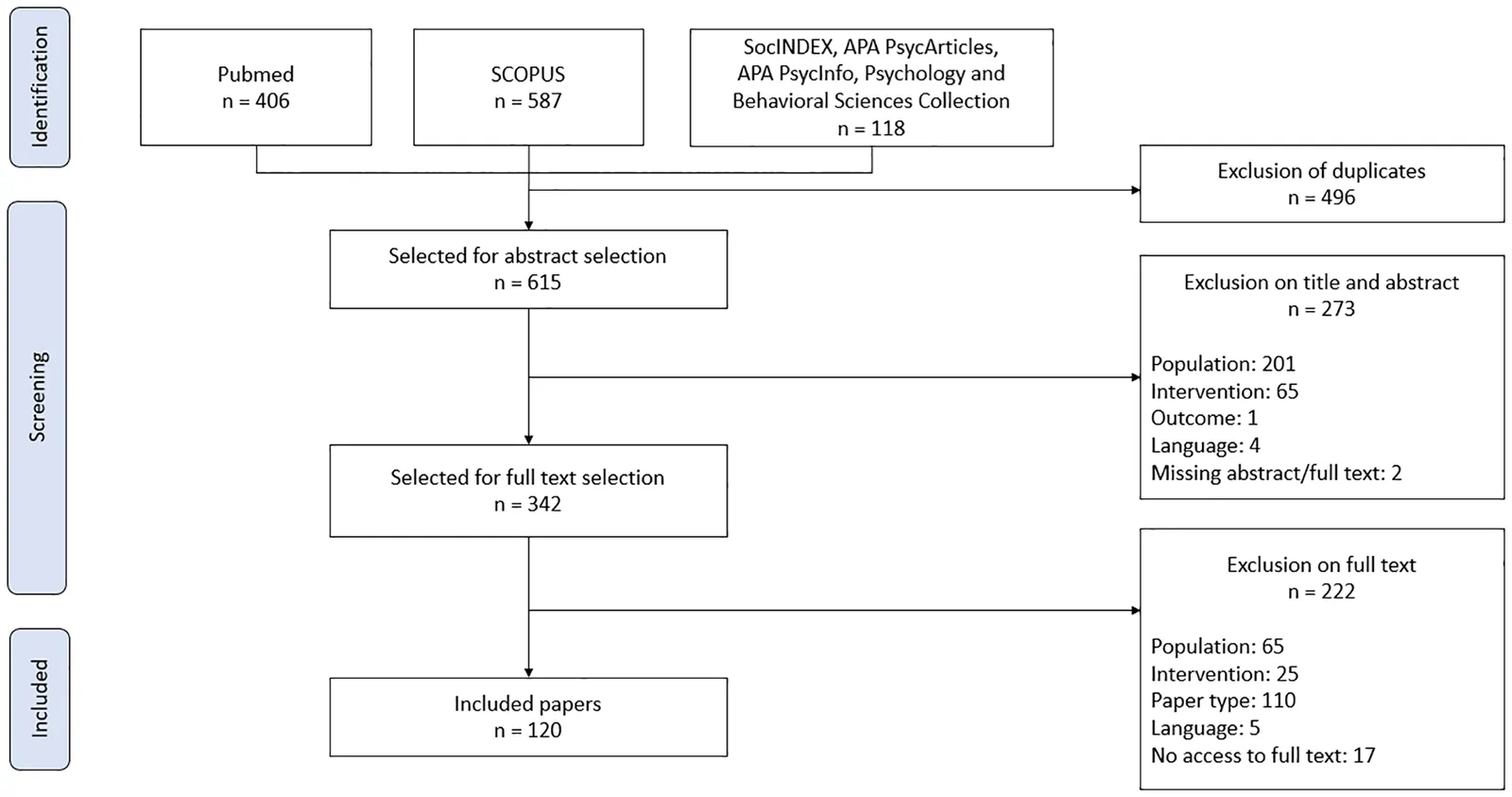
Flowchart of the scoping review on PrEP among female sex workers.

### Global cartography of PrEP papers on FSWs worldwide

Among the 120 included studies, n = 75 were conducted in Eastern and Southern Africa, n = 15 in Western and Central Africa, n = 2 in the Middle East and North Africa, n = 12 in Western and Central Europe and North America, n = 1 in Eastern Europe and Central Asia, n = 10 in Asia and the Pacific and n = 6 in Latin America and the Caribbean (Fig 2); one study was conducted in both Tanzania and the Dominican Republic [14].

**Fig 2:**
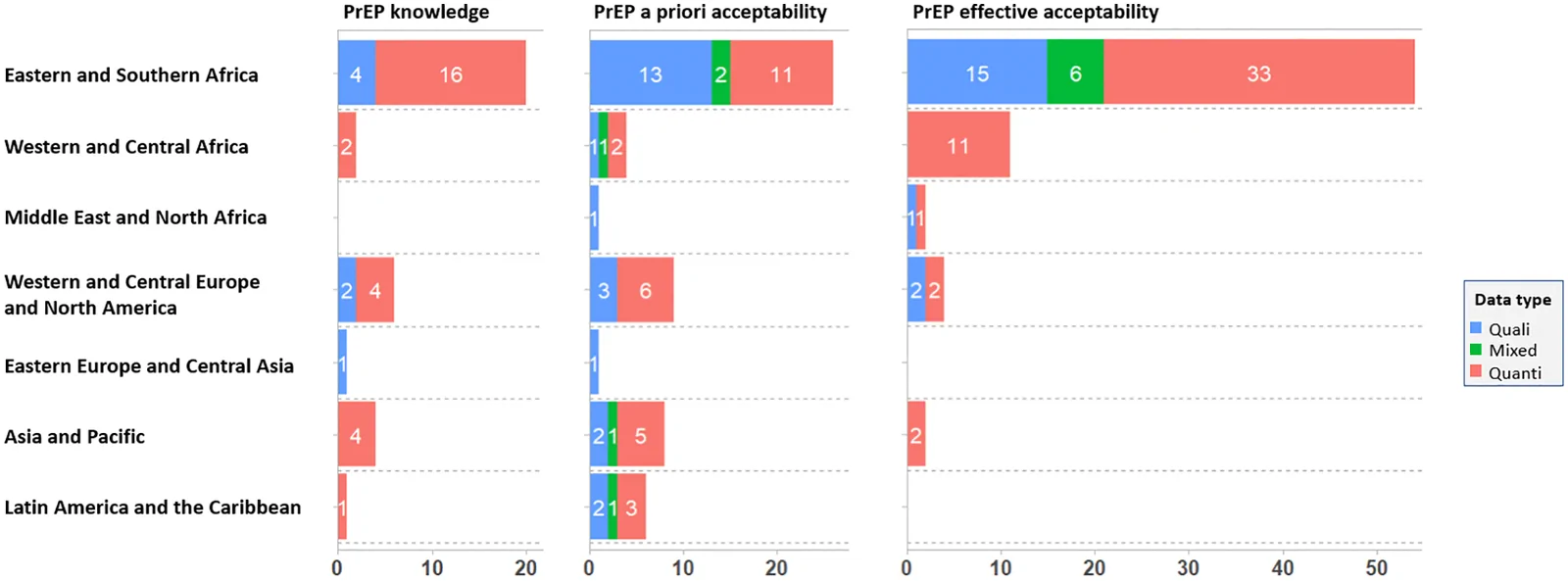
Cartography of original papers published on PrEP among female sex workers until the end of 2024, stratified by UNAIDS region and data type. Note: one paper can cover several outcomes with the same or different data types.

### PrEP knowledge (n=34 studies, S2 Table)

Quantitative data showed that before PrEP became widely available, few FSWs reported having heard of PrEP [15–27]. However, once PrEP was integrated into demonstration projects or national programs, PrEP knowledge increased rapidly, as especially shown in Eastern and Southern Africa [28–33]. Four qualitative studies conducted in Eastern and Southern Africa [34–36] and in Eastern Europe and Central Asia [37] highlighted some misconceptions about PrEP among FSWs, such as the confusion between PrEP and post-exposure prophylaxis or between PrEP and ARV treatment. A study conducted in South Africa emphasized the time needed for FSWs to become confident in using PrEP, given that for decades, HIV prevention messages had stressed that condoms were the only way to prevent HIV transmission [34]. Another study conducted in the USA reported that other priorities of FSWs, such as substance use or housing, could be barriers to PrEP education [38].

### PrEP a priori acceptability (willingness/interest) (n=55 studies, S3 Table)

*Oral PrEP:* FSWs were generally very interested in taking oral PrEP [16,22,23,25,31,39–49]. Qualitative studies showed that willingness to use PrEP was mostly related to the importance of self-care and self-love [15–53]. Oral PrEP was indeed perceived as an added protection during sex work in case of condomless sex because of client-perpetrated sexual violence [14,15,19,35,46,54,55], condom break [15,46,51,54,56], alcohol or drug consumption [35,57,58] or during sexual intercourse with regular partners with whom FSWs do not always use condoms [46,54,56,59,60]. PrEP knowledge was also found as a key determinant of PrEP interest in both quantitative and qualitative studies [21–23,29,42,58,61–63]. Other factors positively associated with high oral PrEP interest in quantitative studies included having an STI history [15,22,23,58] or HIV-risks, including recent unprotected intercourse [42,64]. Some FSWs also reported that oral PrEP could be a way to earn more money [15,35,55,65]. However, one of the main barriers to *a priori* oral PrEP acceptability included concerns regarding adherence to a daily pill, especially considering that FSWs can have unpredictable lives, which can make it difficult to maintain a regular medication routine, or in the context of alcohol use, which can affect the ability to remember daily pill-taking [14,15,26,37,40,46,51–55,57,60,62,66,67]. Other important barriers regarding PrEP willingness included fear of stigma, as PrEP can be confused with ARVs used for HIV treatment or inadvertently reveal sex work engagement [14,23,29,40,45,46,52,53,55,63,66,68,69] as well as concerns about potential side-effects [14,23,29,37,46,50–52,54,55,57,60,61,65]. In some studies, FSWs reported competing responsibilities (e.g., chores, work, rest, or children), and thus low priority regarding healthcare with no time to spend in clinics to receive PrEP [16,53,68], especially when these healthcare facilities were opened at limited times [63]. The cost of PrEP was also found as a potential perceived obstacle to PrEP [22,24,42,57,60,63].

*Other forms of PrEP:* Nineteen studies were conducted on long-acting PrEP a priori acceptability, including injections [14,15,26,38,43,47,56,64,65,67,70–73], implants [39,47,66,67], vaginal rings [15,19,43,47,56,64,71–73] or microarray patches [74,75]. Interest in other forms of PrEP, such as gels [43,47,65,73,76], was also investigated. As for oral PrEP, long-acting PrEP was described as an effective way to protect against HIV [64,73]. FSWs expressed a preference for long-acting PrEP as it can alleviate the burden of daily oral PrEP [14,15,38,55,56,65–67,71,72,74,75], even if adherence challenges were perceived with the 2-month injectable PrEP requiring regular follow-up [38,56]. In studies comparing several long-acting PrEP, injections were more often preferred [43,70,73]. Injections and microarray patches were liked as they facilitate discreet use [15,56,71,75] while implants were perceived as more difficult to hide [67]. In some studies, FSWs also feared that a vaginal ring might be invasive and be felt or displaced, especially during sexual intercourse [15,56,71,72]. Other studies also highlighted some concerns regarding potential long-acting PrEP irreversible side-effects [14,15,26,67,71,75].

### PrEP effective acceptability (uptake/use, retention, adherence) (n = 73 studies, S4 Table)

In all the UNAIDS regions, uptake of oral PrEP ranged from relatively low levels (<30%) [17,27,31,33,39,41,58,77–87] to over 80% [88–95]. Retention at 1 month also showed wide variability, often about or below 50% [53,83,88,96–101] to >75% [48,80,92,94,102–106]. Only a few studies showed retention >75% at 6 months [48,92,94,103,107] and 1 year >75% [48,94,95,108].

Oral PrEP adherence, most often measured as self-declared, also varied, with several studies showing rates below 50% [33,79,82,87,89,109] to >70% [84,88,94,95,110,111]. Three studies showed different PrEP adherence numbers according to how it is measured: higher if self-reported while lower in TDF concentration in blood and in pill count [84,95,106].

Regarding socio-demographic characteristics, most of the studies showed that younger individuals were more likely to initiate PrEP [28,83,108,112] while, once on PrEP, they were less likely to stay on PrEP [27,82,83,87,92,97,111,113]. PrEP use was also higher in FSWs with a higher education level [28,77,84] or who had or wanted children [49,102,114].

Perceived benefits for HIV prevention, especially in the case of condom breakage or condomless sex, was one of the most cited motivators to initiate PrEP [30,34,38,53,72,85,114–123], while using condoms was associated with high PrEP adherence [82,89,102]. Alongside, in the quantitative studies, oral PrEP uptake, retention and adherence were associated with STI history [17,58,111], longer duration of sex work [33,77,82], high number of clients [30,33,100,111] and already experienced physical violence from a client or from authorities [84,124]. In this context, a study suggested that FSWs who were on PrEP for more than one year were less worried about HIV [125].

The burden of daily PrEP was cited by FSWs as a major barrier to oral PrEP initiation [30,33,41,83,85,104,121,122], retention and adherence [56,59,92,109,110,115,122,123,126], especially in the context of alcohol use, which can interfere with remembering to take the pills daily [59,72,82,118,122,126], or lack of housing, which can make it difficult to store medication safely [38]. Fear of stigma, especially because of the confusion between PrEP and antiretroviral treatment, either in sex work sites or when FSWs went back home, was also an important reason for FSWs to not start oral PrEP [17,30,33,34,36,41,72,117,118,121] or to stop it [30,33,56,83,109,110,115,117,122,123]. In this context, high mobility of FSWs was an obstacle to oral PrEP retention and adherence [99,104,110,113,117,118]. Fear of side effects could also prevent PrEP initiation [17,30,38,41,104,117,121,122,127], while experiencing side effects (e.g., headaches, gastrointestinal symptoms such as stomach pain, nausea, vomiting, bloating and diarrhoea, nightmares, back pain, menstrual changes, swelling, sweating, loss of appetite, weight loss and fatigue) was found as a barrier to PrEP continuation [30,33,49,53,56,59,72,83,95,99,104,109,110,115,117,118,121–123,126,128].

Challenges in PrEP use were also related to difficulties in accessing health facilities where PrEP was delivered, because of distance and transportation costs [17,33,72,115,123], high number of appointments required before PrEP initiation, and time for clinic follow-up visits, with sometimes clinic hours conflicting with work schedules [83,121,127] or being unfamiliar with the clinic delivery schedule [115,117].

Encouragement and support from others (family, friends, colleagues) were facilitators for FSWs to initiate and stay on PrEP [30,34,84,89,104,114,118,121]. In addition, several remembered strategies to take pills, such as phone alarms, placing the pill bottle in a routine location, or taking PrEP alongside oral contraception, were identified as helping FSWs to adhere to oral PrEP [34,104,118,123]. The use of SMS support or mHealth applications also increased retention in PrEP programs [96,98]. Yet, communication issues, including phone number changes, intermittent phone access [117,128], or housing instability [128] could limit PrEP retention strategies through phones or peer navigators (i.e., trained peers with shared community experience who support FSWs in accessing PrEP).

Only one study explored facilitators and barriers to injectable CAB use [126]: even if some FSWs complained about some pain, they mostly tolerated it and found it more convenient than a daily oral regimen as it fits better into women’s lifestyles, especially when traveling or having unpredictable work. FSWs also appreciated the long-lasting protective effect of the injectable CAB and found it more discreet, allowing them to maintain privacy with their sexual clients or friends and family.

### Other outcomes of PrEP use (n=7 studies, S5 Table)

Seven studies have explored the impact of PrEP use on sexual risks. None of these studies found evidence of increased sexual risk-taking attributable to PrEP use, based on either self-reported behaviors or biological measurements [94,95,129–131], nor an increase in sexually transmitted infections [95,130]. However, one paper underlined that PrEP-related stigma was relatively high among FSWs on PrEP [132].

## Discussion

This scoping review provides a comprehensive global synthesis of PrEP research among cisgender FSWs, spanning all UNAIDS regions. Compared to the extensive literature on PrEP among men who have sex with men (with 2,700 references identified on Pubmed by the end of 2024), the overall literature on PrEP among FSWs is relatively scarce. Most of the studies were conducted in Eastern and Southern Africa, while the literature remains scarce in the other UNAIDS regions, particularly in the Middle East and North Africa and Eastern Europe and Central Asia. In these regions, where sex work can be highly criminalized or severely punished, leading to FSWs being at greater risk of HIV [133–135], research access and funding may be limited.

Once knowledge was established, *a priori* acceptability of PrEP (interest and willingness) was consistently high across settings, yet effective engagement (uptake, retention and adherence) remained suboptimal. The determinants of oral PrEP engagement were numerous and occurred at various levels (Fig 3). Taken together, these findings support a socio-ecological understanding of PrEP engagement among FSWs that is adapted to the context. While socio-ecological models typically distinguish between the individual, interpersonal, and structural levels, our findings highlight the importance of also considering the sex work context as a distinct intermediary layer.

**Fig 3:**
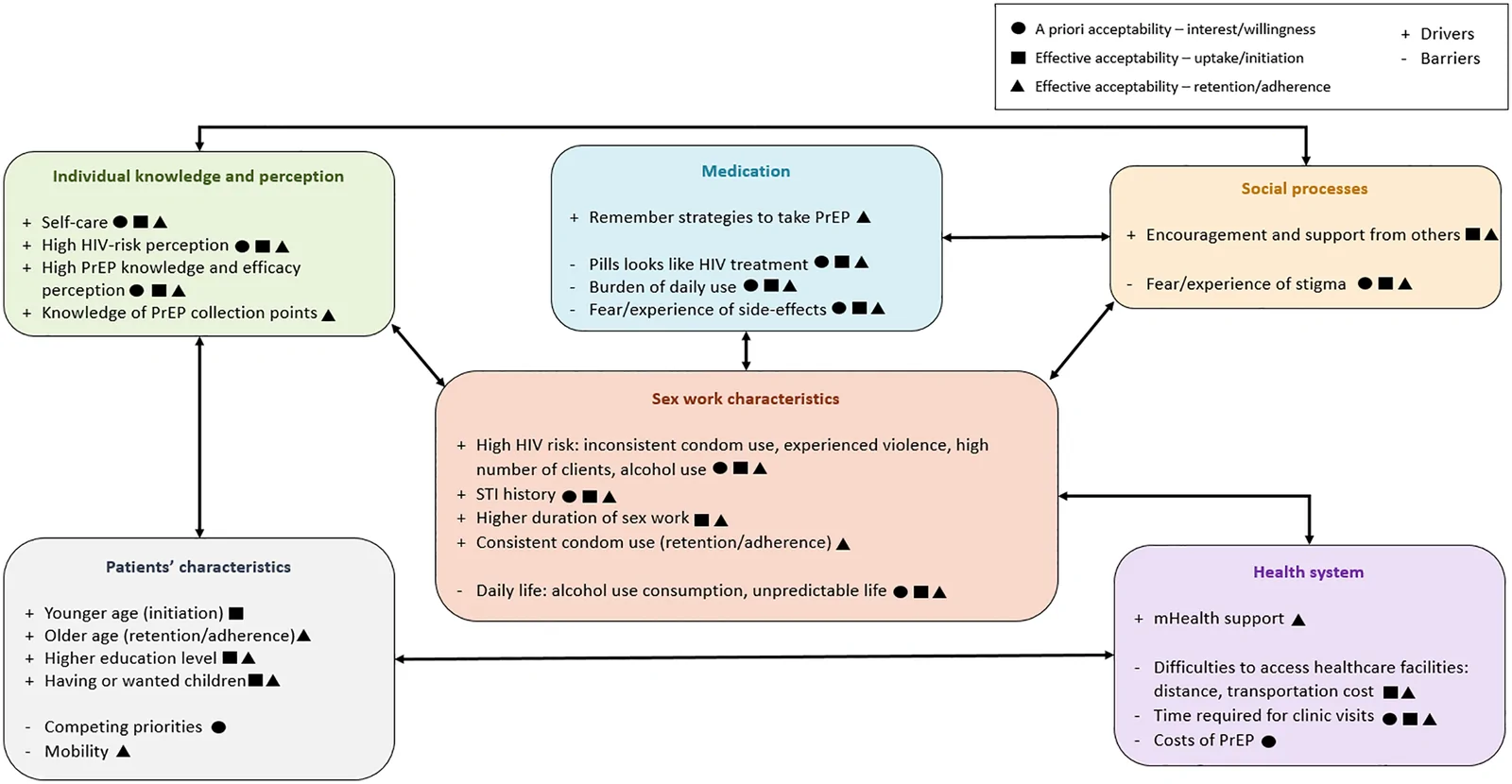
Drivers and barriers to oral PrEP engagement among female sex workers.

Perceived HIV risk due to the realities of sex work – particularly in the context of a high number of clients, condom breakage, client-perpetrated violence, and substance use – was one of the main motivators for PrEP *a priori* and effective acceptability. In this context, narratives of self-care and self-love were particularly salient in qualitative studies, presenting PrEP as an empowering prevention strategy. Individual knowledge and perception of PrEP, especially with regard to its efficacy, increased willingness to use PrEP. Similarly, PrEP acceptability was greater among FSWs with a higher level of education. These results highlight the need to reinforce PrEP awareness among FSWs [14,136–138]. It would be important to include information on how to manage side effects [136], as these could also strongly influence PrEP engagement and retention, as shown in studies included in this scoping review.

However, these individual motivations could be outweighed by social determinants. Stigma could decrease PrEP acceptability, from initial willingness to long-term adherence. In the included studies, FSWs may fear being perceived as HIV-positive if there is confusion between PrEP and HIV treatment, or they may be concerned that taking PrEP could lead to disclosure of their sex work activity. Conversely, encouragement and support from others could facilitate PrEP engagement. These results highlight the need for community-level education strategies and individualized support to destigmatize PrEP [14,136,137]. Several studies also underlined that PrEP implementation should rely on friendly services, with trusted providers who understand their needs and remain non-judgmental [35,37,50,54,56,62,68,121,138], while also ensuring privacy and confidentiality [50,71].

Several structural determinants related to both sex work and health system characteristics also posed challenges to PrEP use and retention. Several studies included in the scoping review reported that FSWs experienced constraints in accessing clinics, such as clinic opening hours conflicting with work schedules or other competing priorities, transportation costs, and the need to attend multiple pre-initiation visits. In this context, improving accessibility and flexibility of services, including differentiated service delivery models that adapt where, when, and how PrEP is delivered to the needs and preferences of FSWs, may help address these barriers. This could include outreach or easily accessible services, short waiting times, and convenient opening hours [16,35,54,68,71,121,138]. In addition, integrating PrEP into existing services, delivered alongside sexual and reproductive health services (e.g., family planning, HIV testing, STI care, and cervical cancer screening) or within one-stop-shop models, could increase FSWs’ motivation to access care [16,35,68,138].

FSWs also faced the burden of the daily oral PrEP regimen, which does not align with the realities of sex work and their unpredictable schedules. This was particularly challenging in contexts involving alcohol use, unstable housing, or frequent travel outside of sex work sites. Digital adherence tools and reminder strategies have shown promise [139], but may be limited in contexts of housing instability or disrupted phone access. In this context, although research on long-acting PrEP remains limited among FSWs, the available studies suggest a strong interest in these methods, particularly injectable PrEP [140]. Such HIV prevention methods are perceived as reducing the daily pill burden, enhancing discretion, and potentially being better suited to unpredictable schedules and mobility. These potential advantages could be further enhanced when long-acting PrEP is delivered through differentiated service delivery models adapted to FSWs’ needs, for example through outreach or community-based services that reduce the need for frequent clinic visits. However, although these new forms of PrEP have only recently been recommended [5–7], evidence remains scarce regarding their uptake, retention, and real-world implementation among FSWs.

Importantly, the review found no evidence of an increase in sexual risk-taking or STI acquisition associated with PrEP use. These findings are consistent with those of other studies conducted among different populations [141] and contribute to the ongoing debate about behavioral disinhibition. However, the available evidence remains insufficient to exclude risk compensation in all contexts or to draw conclusions about broader population-level trends in bacterial STI incidence.

All these findings are broadly consistent with those reported among other key populations, as shown in a recent narrative review [142] identifying facilitators and barriers to oral PrEP uptake at the individual level (e.g., awareness, HIV risk perception, and competing priorities), interpersonal and community levels (e.g., stigma, provider-client interactions, and peer and community support), health system and institutional levels (e.g., opening hours), structural and policy levels (e.g., poverty and economic hardship, gender inequality, and criminalization). However, our findings also highlight barriers that may be particularly relevant to FSWs, including the unpredictability and mobility associated with sex work, concerns about disclosure of sex work, and the need for services adapted to their working conditions. These findings underscore the importance of developing PrEP delivery approaches that are responsive to the specific social and structural contexts of each key population.

This review has several strengths. First, the use of multiple multidisciplinary bibliographic databases enabled broad coverage of relevant studies across various disciplines. Additionally, the independent screening process minimized bias in study selection. Second, it integrates both quantitative and qualitative evidence to allow for a nuanced understanding of the determinants of PrEP acceptability. Third, the resulting conceptual framework reflects a comprehensive synthesis of the available evidence on PrEP among cisgender FSWs worldwide through December 2024. However, subsequent research has been published since the search was conducted and may have further enriched or modified these findings, highlighting the need for continued monitoring of the evolving evidence on PrEP among sex workers.

This review has other limitations. As a scoping review, we did not formally assess the methodological quality or risk of bias of the included studies. This may affect the robustness and reliability of some findings and make it more difficult to assess the strength of the available evidence. Furthermore, while the screening process was conducted independently, a single senior reviewer extracted specific results focusing on each outcome, which may have introduced subjectivity. Heterogeneity in adherence measurement and program contexts limited comparability. Lastly, publication bias remains a potential concern, as the results of some PrEP implementation projects, particularly those conducted in programs or non-research settings, may not have been published. The geographical concentration of the evidence in Eastern and Southern Africa may also limit the transferability of our findings to other settings. The organization and working conditions of sex work, healthcare systems, access to HIV prevention services, and the availability and implementation of PrEP may differ substantially across regions. Consequently, determinants of PrEP engagement and the feasibility or acceptability of specific service delivery models identified in the included studies may not be directly applicable to FSWs in other contexts.

In conclusion, this scoping review revealed that, although there is significant interest in PrEP, sustained engagement with oral PrEP is influenced by various constraints related to sex work, social processes, and the healthcare system. This scoping review also highlights several research priorities. First, research should expand beyond the geographical concentration of existing evidence in Eastern and Southern Africa, particularly towards regions where evidence remains scarce, including the Middle East and North Africa and Eastern Europe and Central Asia. Second, as biomedical HIV prevention research continues to advance, efforts should focus on addressing the specific challenges that FSWs face in preventing HIV and identifying the most effective, suitable, and equitable HIV prevention strategies, including long-acting and other non-daily PrEP options. Research should determine whether these approaches can overcome barriers associated with daily pill-taking, mobility, unstable housing, unpredictable schedules, and concerns about privacy, and identify which prevention options are preferred by different groups of FSWs. Finally, future research should include more longitudinal and implementation studies to improve sustained PrEP engagement under real-world conditions. Such studies should evaluate differentiated service delivery models and assess how PrEP delivery can be better aligned with the lived realities of sex work, including mobility, stigma, and constraints to service access. Together, these priorities can help translate the high interest in PrEP among FSWs into sustained HIV prevention impact.

## Supporting information

S1 Fig Research equations. Scoping review on PrEP among female sex workers.
(PNG)

S1 Table PRISMA-ScR checklist. [Scoping review on PrEP among female sex workers - until end 2024].
(DOCX)

S2 Table PrEP knowledge. [Scoping review on PrEP among female sex workers - until end 2024].
(DOCX)

S3 Table PrEP a priori acceptability [Scoping review on PrEP among female sex workers - until end 2024]. (DOCX)

S4 Table PrEP effective acceptability [Scoping review on PrEP among female sex workers - until end 2024]. (DOCX)

S5 Table Other outcomes of PrEP use [Scoping review on PrEP among female sex workers - until end 2024]. (DOCX)

## References

[1] Korenromp EL, Sabin K, Stover J, Brown T, Johnson LF, Martin-Hughes R. New HIV infections among key populations and their partners in 2010 and 2022, by world region: A multisources estimation. J Acquir Immune Defic Syndr. 2024;95(1S):e34–45. doi: 10.1097/QAI.0000000000003340 38180737 PMC10769164

[2] Simegn MB, Geremew H, Chinkey FD, Alemneh ZT, Golla EB, Abate A, et al. Inconsistent condom use and its associated factors among female sex workers in African countries: Systematic review and meta-analysis. PLoS One. 2026;21(4):e0346903. doi: 10.1371/journal.pone.0346903 41961838 PMC13068245

[3] Segosebe K, Kirwan M, Davis KC. Barriers to condom negotiation and use among female sex workers in the United States and United States-Mexico border cities: A systematic review. AIDS Behav. 2023;27(9):2855–64. doi: 10.1007/s10461-023-04009-z 36786938

[4] World Health Organization. Guideline on when to start antiretroviral therapy and on pre-exposure prophylaxis for HIV [Internet]. 2015 [cited 2021 Jun 22]. Available from: https://www.who.int/publications/i/item/9789241509565 26598776

[5] World Health Organization. Consolidated Guidelines on HIV prevention, testing, treatment, service delivery and monitoring: recommendations for a public health approach [Internet]. World Health Organization; 2021 [cited 2021 Feb 9]. Available from: https://iris.who.int/server/api/core/bitstreams/a4c03ed9-5446-4958-a26f-10e505c2679c/content 34370423

[6] World Health Organization. Guidelines on long-acting injectable cabotegravir for HIV prevention [Internet]. World Health Organization; 2022 [cited 2026 Feb 25]. Available from: https://iris.who.int/server/api/core/bitstreams/f3c82c5c-97c5-4651-a4c2-a909eda7eddc/content 36417549

[7] World Health Organization. Guidelines on lenacapavir for HIV prevention and testing strategies for long-acting injectable pre-exposure prophylaxis [Internet]. World Health Organization; 2025 [cited 2026 Feb 25]. Available from: https://iris.who.int/server/api/core/bitstreams/30ec1404-5338-4459-a6f0-a187c85f3ff1/content 40743386

[8] Flexner C. The future of long acting agents for PrEP. Curr Opin HIV AIDS. 2022;17(4):192–8. doi: 10.1097/COH.0000000000000735 35762373 PMC9467455

[9] Glick JL, Russo R, Jivapong B, Rosman L, Pelaez D, Footer KHA, et al. The PrEP care continuum among cisgender women who sell sex and/or use drugs globally: A systematic review. AIDS Behav. 2020;24(5):1312–33. doi: 10.1007/s10461-019-02733-z 31807973 PMC7899785

[10] Mpirirwe R, Segawa I, Ojiambo KO, Kamacooko O, Nangendo J, Semitala FC, et al. HIV pre-exposure prophylaxis uptake, retention and adherence among female sex workers in sub-Saharan Africa: A systematic review. BMJ Open. 2024;14(4):e076545. doi: 10.1136/bmjopen-2023-076545 38670600 PMC11057315

[11] Tricco AC, Lillie E, Zarin W, O’Brien KK, Colquhoun H, Levac D, et al. PRISMA extension for scoping reviews (PRISMA-ScR): Checklist and explanation. Ann Intern Med. 2018;169(7):467–73. doi: 10.7326/M18-0850 30178033

[12] Kvarnström K, Westerholm A, Airaksinen M, Liira H. Factors contributing to medication adherence in patients with a chronic condition: A scoping review of qualitative research. Pharmaceutics. 2021;13(7):1100. doi: 10.3390/pharmaceutics13071100 34371791 PMC8309154

[13] Peh KQE, Kwan YH, Goh H, Ramchandani H, Phang JK, Lim ZY, et al. An adaptable framework for factors contributing to medication adherence: Results from a systematic review of 102 conceptual frameworks. J Gen Intern Med. 2021;36(9):2784–95. doi: 10.1007/s11606-021-06648-1 33660211 PMC8390603

[14] Mantsios A, Muraleetharan O, Donastorg Y, Perez M, Gomez H, Shembilu C, et al. “She is the one who knows”: A qualitative exploration of oral and injectable PrEP as part of a community empowerment approach to HIV prevention among female sex workers in the Dominican Republic and Tanzania. PLOS Glob Public Health. 2022;2(9):e0000981. doi: 10.1371/journal.pgph.0000981 36712835 PMC9880901

[15] Beckham SW, Mantsios A, Galai N, Likindikoki S, Mbwambo J, Davis W, et al. Acceptability of multiple modalities of pre-exposure prophylaxis (PrEP) among female sex workers in Tanzania: A mixed-methods study. BMJ Open. 2022;12(8):e058611. doi: 10.1136/bmjopen-2021-058611 35977762 PMC9389123

[16] Lancaster KE, Lungu T, Bula A, Shea JM, Shoben A, Hosseinipour MC, et al. Preferences for pre-exposure prophylaxis service delivery among female sex workers in Malawi: A discrete choice experiment. AIDS Behav. 2020;24(5):1294–303. doi: 10.1007/s10461-019-02705-3 31720955 PMC7995177

[17] Shibesh BF, Admas AB, Lake AW, Getu SB, Worede DT. Uptake of retroviral pre-exposure prophylaxis and its associated factors among female sex workers, Northwest Ethiopia. AIDS Res Ther. 2023;20(1):77. doi: 10.1186/s12981-023-00573-5 37926821 PMC10626692

[18] Sosnowy C, Tao J, Nunez H, Montgomery MC, Ndoye CD, Biello K, et al. Awareness and use of HIV pre-exposure prophylaxis among people who engage in sex work presenting to a sexually transmitted infection clinic. Int J STD AIDS. 2020;31(11):1055–62. doi: 10.1177/0956462420943702 32753003

[19] Peitzmeier SM, Tomko C, Wingo E, Sawyer A, Sherman SG, Glass N, et al. Acceptability of microbicidal vaginal rings and oral pre-exposure prophylaxis for HIV prevention among female sex workers in a high-prevalence US city. AIDS Care. 2017;29(11):1453–7. doi: 10.1080/09540121.2017.1300628 28271718 PMC5591065

[20] Tomko C, Park JN, Allen ST, Glick J, Galai N, Decker MR, et al. Awareness and interest in HIV pre-exposure prophylaxis among street-based female sex workers: Results from a US context. AIDS Patient Care STDS. 2019;33(2):49–57. doi: 10.1089/apc.2018.0182 30632769 PMC6386071

[21] Garg PR, Uppal L, Kurian K, Heng T, Akhtar F, Mehra S. Pre-exposure prophylaxis (PrEP) awareness, willingness to use, and preferred places for access among the Cambodian female entertainment workers: A cross-sectional study. AIDS Behav. 2022. doi: 10.1007/s10461-022-03877-1 36348190

[22] Peng B, Yang X, Zhang Y, Dai J, Liang H, Zou Y, et al. Willingness to use pre-exposure prophylaxis for HIV prevention among female sex workers: a cross-sectional study in China. HIV AIDS (Auckl). 2012;4:149–58. doi: 10.2147/HIV.S33445 23055781 PMC3463398

[23] Ye L, Wei S, Zou Y, Yang X, Abdullah AS, Zhong X, et al. HIV pre-exposure prophylaxis interest among female sex workers in Guangxi, China. PLoS One. 2014;9(1):e86200. doi: 10.1371/journal.pone.0086200 24465956 PMC3899205

[24] Poon AN, Han L, Li Z, Zhou C, Li Y, Huang L, et al. Acceptability and willingness of HIV pre-exposure prophylaxis amongst female sex workers in China. AIDS Care. 2019;31(12):1555–64. doi: 10.1080/09540121.2019.1612001 31046413

[25] Logie CH, Wang Y, Lalor P, Williams D, Levermore K. Pre and post-exposure prophylaxis awareness and acceptability among sex workers in Jamaica: A cross-sectional study. AIDS Behav. 2021;25(2):330–43. doi: 10.1007/s10461-020-02972-5 32666244

[26] Lunkuse JF, Kamacooko O, Muturi-Kioi V, Chinyenze K, Kuteesa MO, Price MA, et al. Low awareness of oral and injectable PrEP among high-risk adolescent girls and young women in Kampala, Uganda. BMC Infect Dis. 2022;22(1):467. doi: 10.1186/s12879-022-07398-z 35578163 PMC9109298

[27] Olakunde BO, Folala-Anoemuah Y, Ujam C, Ndukwe CD, Olaifa Y, Yahaya HB, et al. Awareness and uptake of oral pre-exposure prophylaxis among adolescent and young key populations in Nigeria: A secondary data analysis of the 2020 Integrated Biological & Behavioural Surveillance Survey. AIDS Care. 2024;36(1):146–52. doi: 10.1080/09540121.2023.2254547 37683258

[28] Ademe B, Eyob Ketema Anagaw TF. Utilization of HIV pre-exposure prophylaxis and associated factors among female sex workers in Bahir Dar City, North-West Ethiopia, cross-sectional study. Int J Gen Med. 2023;16:6015. doi: 10.2147/IJGM.S443477 PMC10749782 38146329

[29] Berhe TT, Elefie Asfaw Tedla GW. Assessment of acceptance and associated factors of HIV pre-exposure prophylaxis among commercial female sex workers in drop-in centers selected sub-cities of Addis Ababa, Ethiopia. Front Public Health. 2024;12:1462648. doi: 10.3389/fpubh.2024.1462648 PMC11638227 39678245

[30] Pillay D, Stankevitz K, Lanham M, Ridgeway K, Murire M, Briedenhann E, et al. Factors influencing uptake, continuation, and discontinuation of oral PrEP among clients at sex worker and MSM facilities in South Africa. PLoS One. 2020;15(4):e0228620. doi: 10.1371/journal.pone.0228620 32352969 PMC7192496

[31] Faini D, Munseri P, Sandstrom E, Hanson C, Bakari M. Awareness, willingness and use of HIV pre-exposure prophylaxis among female sex workers living in Dar-es-Salaam, Tanzania. AIDS Behav. 2022. doi: 10.1007/s10461-022-03769-4 PMC9852168 35838859

[32] Bongomin F, Kibone W, Okot J, Ouma S, Madraa G, Ojara FW, et al. Pre-exposure prophylaxis use among female sex workers in Gulu city, Uganda: A community-based cross-sectional study. Ther Adv Infect Dis. 2023;10. doi: 10.1177/20499361231199550 37693859 PMC10492468

[33] Hensen B, Machingura F, Busza J, Birdthistle I, Chabata ST, Chiyaka T, et al. How can we support the use of oral PrEP among young women who sell sex? A PrEP Cascade analysis. J Acquir Immune Defic Syndr. 2021;88(1):45–56. doi: 10.1097/QAI.0000000000002733 34050101 PMC8357043

[34] Eakle R, Bothma R, Bourne A, Gumede S, Motsosi K, Rees H. “I am still negative”: Female sex workers’ perspectives on uptake and use of daily pre-exposure prophylaxis for HIV prevention in South Africa. PLoS One. 2019;14(4):e0212271. doi: 10.1371/journal.pone.0212271 30964874 PMC6456175

[35] Bazzi AR, Yotebieng K, Otticha S, Rota G, Agot K, Ohaga S, et al. PrEP and the syndemic of substance use, violence, and HIV among female and male sex workers: A qualitative study in Kisumu, Kenya. J Int AIDS Soc. 2019;22(4):e25266. doi: 10.1002/jia2.25266 30983147 PMC6462807

[36] Makhakhe NF, Sliep Y, Meyer-Weitz A. “Whatever is in the ARVs, is also in the PrEP” challenges associated with oral pre-exposure prophylaxis use among female sex workers in South Africa. Front Public Health. 2022;10:691729. doi: 10.3389/fpubh.2022.691729 35784260 PMC9247347

[37] McCrimmon T, Frye V, Darisheva M, Starbird L, Cordingley O, Terlikbayeva A, et al. “Protected Means Armed”: Perspectives on pre-exposure prophylaxis (PrEP) among women who engage in sex work and use drugs in Kazakhstan. AIDS Educ Prev. 2023;35(5):347–61. doi: 10.1521/aeap.2023.35.5.347 37843909 PMC10901454

[38] Harris MT, Weinberger E, O’Brien C, Althoff M, Paltrow-Krulwich S, Taylor JL, et al. PrEP facilitators and barriers in substance use bridge clinics for women who engage in sex work and who use drugs. Addict Sci Clin Pract. 2024;19(1):47. doi: 10.1186/s13722-024-00476-4 38831359 PMC11145858

[39] Little KM, Flomen L, Hanif H, Anderson SM, Thurman AR, Clark MR, et al. HIV pre-exposure prophylaxis implant stated preferences and priorities: Results of a discrete choice experiment among women and adolescent girls in Gauteng Province, South Africa. AIDS Behav. 2022;26(9):3099–109. doi: 10.1007/s10461-022-03658-w 35360893 PMC9371991

[40] Muwonge TR, Nsubuga R, Brown C, Nakyanzi A, Bagaya M, Bambia F, et al. Knowledge and barriers of PrEP delivery among diverse groups of potential PrEP users in Central Uganda. PLoS One. 2020;15(10):e0241399. doi: 10.1371/journal.pone.0241399 33112907 PMC7592843

[41] Witte SS, Filippone P, Ssewamala FM, Nabunya P, Bahar OS, Mayo-Wilson LJ, et al. PrEP acceptability and initiation among women engaged in sex work in Uganda: Implications for HIV prevention. EClinicalMedicine. 2022;44:101278. doi: 10.1016/j.eclinm.2022.101278 35128367 PMC8808048

[42] Mudzviti T, Dhliwayo A, Chingombe B, Ngara B, Monera-Penduka TG, Maponga CC, et al. Perspectives on oral pre-exposure prophylaxis use amongst female sex workers in Harare, Zimbabwe. South Afr J HIV Med. 2020;21(1):1039. doi: 10.4102/sajhivmed.v21i1.1039 32158559 PMC7059237

[43] Ortblad KF, Chanda MM, Musoke DK, Ngabirano T, Mwale M, Nakitende A, et al. Acceptability of HIV self-testing to support pre-exposure prophylaxis among female sex workers in Uganda and Zambia: Results from two randomized controlled trials. BMC Infect Dis. 2018;18(1):503. doi: 10.1186/s12879-018-3415-z 30286737 PMC6172754

[44] Blumenthal J, Landovitz R, Jain S, He F, Kofron R, Ellorin E, et al. Pre-exposure prophylaxis perspectives, sociodemographic characteristics, and HIV risk profiles of cisgender women seeking and initiating PrEP in a US demonstration project. AIDS Patient Care STDS. 2021;35(12):481–7. doi: 10.1089/apc.2021.0114 34851726 PMC8905235

[45] Jackson T, Huang A, Chen H, Gao X, Zhang Y, Zhong X. Predictors of willingness to use HIV pre-exposure prophylaxis among female sex workers in Southwest China. AIDS Care. 2013;25(5):601–5. doi: 10.1080/09540121.2012.726341 23062151

[46] Reza-Paul S, Lazarus L, Doshi M, Hafeez Ur Rahman S, Ramaiah M, Maiya R, et al. Prioritizing risk in preparation for a demonstration project: A mixed methods feasibility study of oral pre-exposure prophylaxis (PREP) among female sex workers in South India. PLoS One. 2016;11(11):e0166889. doi: 10.1371/journal.pone.0166889 27880833 PMC5120793

[47] Pines HA, Strathdee SA, Hendrix CW, Bristow CC, Harvey-Vera A, Magis-Rodríguez C, et al. Oral and vaginal HIV pre-exposure prophylaxis product attribute preferences among female sex workers in the Mexico-US border region. Int J STD AIDS. 2019;30(1):45–55. doi: 10.1177/0956462418793038 30170533 PMC6391168

[48] Munyaneza A, Bhutada K, Shi Q, Zotova N, Nsereko E, Muhoza B, et al. High retention among key populations initiated on HIV pre-exposure prophylaxis in Kigali City, Rwanda. J Int AIDS Soc. 2024;27(11):e26392. doi: 10.1002/jia2.26392 39568160 PMC11578929

[49] Martin VO, Tesha NA, Sunguya BF. Uptake of oral hiv pre-exposure prophylaxis (PrEP) and associated factors among female sex workers in Tanga, Tanzania. Viruses. 2023;15(10):2125. doi: 10.3390/v15102125 37896903 PMC10611315

[50] Restar AJ, Tocco JU, Mantell JE, Lafort Y, Gichangi P, Masvawure TB, et al. Perspectives on HIV pre- and post-exposure prophylaxes (PrEP and PEP) among female and male sex workers in Mombasa, Kenya: Implications for integrating biomedical prevention into sexual health services. AIDS Educ Prev Off Publ Int Soc AIDS Educ. 2017;29(2):141–53. doi: 10.1521/aeap.2017.29.2.141 PMC5706461 28467163

[51] Galea JT, Kinsler JJ, Salazar X, Lee S-J, Giron M, Sayles JN, et al. Acceptability of pre-exposure prophylaxis as an HIV prevention strategy: barriers and facilitators to pre-exposure prophylaxis uptake among at-risk Peruvian populations. Int J STD AIDS. 2011;22(5):256–62. doi: 10.1258/ijsa.2009.009255 21571973 PMC3096991

[52] Shea J, Bula A, Dunda W, Hosseinipour MC, Golin CE, Hoffman IF, et al. “The Drug Will Help Protect My Tomorrow”: Perceptions of integrating PrEP into HIV prevention behaviors among female sex workers in Lilongwe, Malawi. AIDS Educ Prev. 2019;31(5):421–32. doi: 10.1521/aeap.2019.31.5.421 31550194 PMC6793990

[53] Lichtwarck HO, Mmbaga EJ, Peter E. What can PrEP do for female sex workers? Unpacking the “effectosphere” of biomedical HIV prevention in Dar es Salaam. Soc Sci Med. 2024;358:117245. doi: 10.1016/j.socscimed.2024.117245 39197277

[54] Eakle R, Bourne A, Mbogua J, Mutanha N, Rees H. Exploring acceptability of oral PrEP prior to implementation among female sex workers in South Africa. J Int AIDS Soc. 2018;21(2):e25081. doi: 10.1002/jia2.25081 29457868 PMC5817972

[55] Sahay S, Verma A, Shewale S, Bangar S, Bijeshkumar A, Angolkar M, et al. Understanding issues around use of oral pre exposure prophylaxis among female sex workers in India. BMC Infect Dis. 2021;21(1):930. doi: 10.1186/s12879-021-06612-8 34496781 PMC8424160

[56] Evens E, Munthali T, Mangunje F, Kotaka ML, Burke HM, Musonda B, et al. Qualitative focus group discussions exploring PrEP method and service delivery preferences among female sex workers and their managers in four Zambian provinces. BMJ Public Health. 2024;2(1):e000483. doi: 10.1136/bmjph-2023-000483 40018231 PMC11816290

[57] Foley S, Keene DE, Shrestha R, Brown S-E, Gautam K, Sutherland RA, et al. Exploring attitudes toward pre-exposure prophylaxis for HIV prevention prior to implementation among female sex workers in Malaysia: Results from a qualitative study. Patient Prefer Adherence. 2024;18:797–807. doi: 10.2147/PPA.S413423 38595805 PMC11003429

[58] Guure C, Afagbedzi S, Torpey K. Willingness to take and ever use of pre-exposure prophylaxis among female sex workers in Ghana. Medicine (Baltimore). 2022;101(5). doi: 10.1097/MD.0000000000028798 PMC8812709 35119051

[59] Kawuma R, Ssemata AS, Bernays S, Seeley J. Women at high risk of HIV-infection in Kampala, Uganda, and their candidacy for PrEP. SSM Popul Health. 2021;13:100746. doi: 10.1016/j.ssmph.2021.100746 33604448 PMC7873676

[60] Becquet V, Nouaman M, Plazy M, Masumbuko J-M, Anoma C, Kouame S, et al. Sexual health needs of female sex workers in Côte d’Ivoire: A mixed-methods study to prepare the future implementation of pre-exposure prophylaxis (PrEP) for HIV prevention. BMJ Open. 2020;10(1):e028508. doi: 10.1136/bmjopen-2018-028508 31919122 PMC6955511

[61] Omo-Emmanuel UK, Udah DC, Airiagbonbu BO, Usman HB, Jegede FE, Aka-Okeke C, et al. Assessment of awareness, willingness, and practice of human immunodeficiency virus pre-exposure prophylaxis among female sex workers in Uyo, Akwa Ibom, Nigeria. Texila Int J Public Health. 2023;11(4). doi: 10.21522/TIJPH.2013.11.04.Art003

[62] Glick JL, Jivapong B, Russo R, Pelaez D, Piser R, Footer K, et al. Cultivating PEARL (Promoting Empowerment and Risk Reduction): Formative Research for a PrEP Intervention Among Female Sex Workers in Baltimore, Maryland. AIDS Behav. 2022;26(8):2664–75. doi: 10.1007/s10461-022-03600-0 35124751 PMC9885761

[63] Jain JP, Strathdee SA, Patterson TL, Semple SJ, Harvey-Vera A, Magis-Rodríguez C, et al. Perceived barriers to pre-exposure prophylaxis use and the role of syndemic factors among female sex workers in the Mexico-United States border region: a latent class analysis. AIDS Care. 2020;32(5):557–66. doi: 10.1080/09540121.2019.1626338 31163975 PMC6891112

[64] Vazquez Guillamet LJ, Valencia J, Ryan P, Cuevas-Tascón G, Del-Olmo-Morales MA, Cobo I, et al. Determinants of intention to use HIV pre-exposure prophylaxis and condom use among a sample of cisgender female sex workers working mostly outdoors in Madrid, Spain. Arch Sex Behav. 2024;53(7):2817–31. doi: 10.1007/s10508-024-02834-4 38858230 PMC11219370

[65] Mack N, Evens EM, Tolley EE, Brelsford K, Mackenzie C, Milford C, et al. The importance of choice in the rollout of ARV-based prevention to user groups in Kenya and South Africa: A qualitative study. J Int AIDS Soc. 2014;17(3 Suppl 2):19157. doi: 10.7448/IAS.17.3.19157 25224616 PMC4164014

[66] Brown MS, Hanif H, Little KM, Clark MR, Thurman AR, Flomen L, et al. End-user research in support of long-acting systemic antiretroviral delivery systems: insights from qualitative research with providers and target users in South Africa. BMC Infect Dis. 2022;22(1):919. doi: 10.1186/s12879-022-07907-0 36482336 PMC9733032

[67] Footer KHA, Lim S, Rael CT, Greene GJ, Carballa-Diéguez A, Giguere R, et al. Exploring new and existing PrEP modalities among female sex workers and women who inject drugs in a U.S. city. AIDS Care. 2019;31(10):1207–13. doi: 10.1080/09540121.2019.1587352 30822101 PMC6663578

[68] Emmanuel G, Folayan M, Undelikwe G, Ochonye B, Jayeoba T, Yusuf A, et al. Community perspectives on barriers and challenges to HIV pre-exposure prophylaxis access by men who have sex with men and female sex workers access in Nigeria. BMC Public Health. 2020;20(1):69. doi: 10.1186/s12889-020-8195-x 31941469 PMC6964078

[69] Ramos SD, Du Bois S. Facilitators and barriers to pre-exposure prophylaxis uptake willingness for full-service sex workers: A social-ecological approach. Sociol Inq. 2024;94(4):890–909. doi: 10.1111/soin.12589 39831188 PMC11737633

[70] Quaife M, Eakle R, Cabrera Escobar MA, Vickerman P, Kilbourne-Brook M, Mvundura M, et al. Divergent preferences for HIV prevention: A discrete choice experiment for multipurpose HIV prevention products in South Africa. Med Decis Making. 2018;38(1):120–33. doi: 10.1177/0272989X17729376 28863752

[71] Mataboge P, Nzenze S, Mthimkhulu N, Mazibuko M, Kutywayo A, Butler V, et al. Planning for decentralized, simplified prEP: Learnings from potential end users in Ga-Rankuwa, gauteng, South Africa. Front Reprod Health. 2023;4:1081049. doi: 10.3389/frph.2022.1081049 36699142 PMC9868940

[72] Moussa AB, Badahdah AM, Hidous K, Barakad R, Diallo F, Traoré M. Barriers to oral PrEP: A qualitative study of female sex workers, PrEP prescribers, policymakers, and community advocates in Morocco. J Int Assoc Provid AIDS Care. 2024;23. doi: 10.1177/23259582241266691 PMC11299217 39099547

[73] Rosen JG, Park JN, Schneider KE, White RH, Beckham SW, Glick JL, et al. Mapping interests in event-driven and long-acting pre-exposure prophylaxis formulations onto the HIV risk environment of street-based female sex workers: A latent class analysis. AIDS Behav. 2022;26(6):1992–2002. doi: 10.1007/s10461-022-03613-9 35362908 PMC9050919

[74] Gachigua SG, Karuga R, Ngunjiri A, Jarrahian C, Coffey PS, Kilbourne-Brook M, et al. Microarray patch for HIV prevention and as a multipurpose prevention technology to prevent HIV and unplanned pregnancy: An assessment of potential acceptability, usability, and programmatic fit in Kenya. Front Reprod Health. 2023;5:1125159. doi: 10.3389/frph.2023.1125159 37168102 PMC10164997

[75] Ismail A, Magni S, Katahoire A, Ayebare F, Siu G, Semitala F, et al. Exploring user and stakeholder perspectives from South Africa and Uganda to refine microarray patch development for HIV PrEP delivery and as a multipurpose prevention technology. PLoS One. 2023;18(8):e0290568. doi: 10.1371/journal.pone.0290568 37651432 PMC10470907

[76] Robertson AM, Syvertsen JL, Martinez G, Rangel MG, Palinkas LA, Stockman JK, et al. Acceptability of vaginal microbicides among female sex workers and their intimate male partners in two Mexico-US border cities: a mixed methods analysis. Glob Public Health. 2013;8(5):619–33. doi: 10.1080/17441692.2012.762412 23398385 PMC3674154

[77] Asmare L, Goshu A, Alemu T, Gebeye E. HIV preexposure prophylaxis practice among urban female sex workers in Northwest Ethiopia: Using generalized structural equation modeling. J Int Assoc Provid AIDS Care. 2024;23. doi: 10.1177/23259582241302900 39632570 PMC11618946

[78] Bien-Gund CH, Ochwal P, Marcus N, Bair EF, Napierala S, Maman S, et al. Adoption of HIV pre-exposure prophylaxis among women at high risk of HIV infection in Kenya. PLoS One. 2022;17(9):e0273409. doi: 10.1371/journal.pone.0273409 36084050 PMC9462728

[79] Cowan FM, Machingura F, Ali MS, Chabata ST, Takaruza A, Dirawo J, et al. A risk-differentiated, community-led intervention to strengthen uptake and engagement with HIV prevention and care cascades among female sex workers in Zimbabwe (AMETHIST): A cluster randomised trial. Lancet Glob Health. 2024;12(9):e1424–35. doi: 10.1016/S2214-109X(24)00235-3 39151978 PMC11345450

[80] Cowan FM, Davey C, Fearon E, Mushati P, Dirawo J, Chabata S, et al. Targeted combination prevention to support female sex workers in Zimbabwe accessing and adhering to antiretrovirals for treatment and prevention of HIV (SAPPH-IRe): A cluster-randomised trial. Lancet HIV. 2018;5(8):e417–26. doi: 10.1016/S2352-3018(18)30111-5 30030134

[81] Jennings Mayo-Wilson L, Yen B-J, Nabunya P, Bahar OS, Wright BN, Kiyingi J, et al. Economic abuse and care-seeking practices for HIV and financial support services in women employed by sex work: A cross-sectional baseline assessment of a clinical trial cohort in Uganda. J Interpers Violence. 2023;38(1–2):NP1920–49. doi: 10.1177/08862605221093680 35510547 PMC9636073

[82] Fearon E, Phillips A, Mtetwa S, Chabata ST, Mushati P, Cambiano V, et al. How can programs better support female sex workers to avoid HIV infection in Zimbabwe? A prevention cascade analysis. J Acquir Immune Defic Syndr. 2019;81(1):24–35. doi: 10.1097/QAI.0000000000001980 30964804 PMC6467580

[83] Were D, Musau A, Mutegi J, Ongwen P, Manguro G, Kamau M, et al. Using a HIV prevention cascade for identifying missed opportunities in PrEP delivery in Kenya: Results from a programmatic surveillance study. J Int AIDS Soc. 2020;23 Suppl 3(Suppl 3):e25537. doi: 10.1002/jia2.25537 32602658 PMC7325512

[84] Shah P, Spinelli M, Irungu E, Kabuti R, Ngurukiri P, Babu H, et al. Factors associated with usage of oral-PrEP among female sex workers in Nairobi, Kenya, assessed by self-report and a point-of-care urine tenofovir immunoassay. AIDS Behav. 2024;28(11):3836–49. doi: 10.1007/s10461-024-04455-3 39136824 PMC11471708

[85] Durosinmi-Etti O, Nwala EK, Oki F, Ikpeazu A, Godwin E, Umoh P, et al. Willingness to pay for HIV prevention commodities among key population groups in Nigeria. Glob Health Sci Pract. 2022;10(5):e2100303. doi: 10.9745/GHSP-D-21-00303 36316139 PMC9622283

[86] Menza TW, Lipira L, Bhattarai A, Leon VC-D, Orellana ER. Prevalence and correlates of transactional sex among women of low socioeconomic status in Portland, OR. BMC Womens Health. 2020;20(1):219. doi: 10.1186/s12905-020-01088-1 33008421 PMC7532610

[87] Rao A, Mhlophe H, Comins C, Young K, Mcingana M, Lesko C. Persistence on oral pre-exposure prophylaxis (PrEP) among female sex workers in eThekwini, South Africa, 2016-2020. PLoS One. 2022;17(3):e0265434. doi: 10.1371/journal.pone.0265434 35290421 PMC8923438

[88] Eakle R, Gomez GB, Naicker N, Bothma R, Mbogua J, Cabrera Escobar MA, et al. HIV pre-exposure prophylaxis and early antiretroviral treatment among female sex workers in South Africa: Results from a prospective observational demonstration project. PLoS Med. 2017;14(11):e1002444. doi: 10.1371/journal.pmed.1002444 29161256 PMC5697804

[89] Mikomangwa WP, Moen K, Mmbaga EJ, Metta E, Kibusi SM, Leshabari MT, et al. HIV pre-exposure prophylaxis use during periods of unprotected sex among female sex workers in Tanga city, Tanzania: A control arm analysis of the pragmatic quasi-experimental trial. Front Public Health. 2024;12:1405765. doi: 10.3389/fpubh.2024.1405765 39081360 PMC11286493

[90] Kagaayi J, Batte J, Nakawooya H, Kigozi B, Nakigozi G, Strömdahl S, et al. Uptake and retention on HIV pre-exposure prophylaxis among key and priority populations in South-Central Uganda. J Int AIDS Soc. 2020;23(8):e25588. doi: 10.1002/jia2.25588 32785976 PMC7421540

[91] Sikazwe I, Musheke M, Chiyenu K, Ngosa B, Pry JM, Mulubwa C, et al. Programme science in action: lessons from an observational study of HIV prevention programming for key populations in Lusaka, Zambia. J Int AIDS Soc. 2024;27 Suppl 2(Suppl 2):e26237. doi: 10.1002/jia2.26237 38982890 PMC11233926

[92] Sarr M, Gueye D, Mboup A, Diouf O, Bao MDB, Ndiaye AJ, et al. Uptake, retention, and outcomes in a demonstration project of pre-exposure prophylaxis among female sex workers in public health centers in Senegal. Int J STD AIDS. 2020;31(11):1063–72. doi: 10.1177/0956462420943704 32819210 PMC7750667

[93] Ben Moussa A, Belhiba O, Sodqi M, Hajouji FZ, Salah NEI, Sakhri N, et al. PrEPare_Morocco a successful community-based PrEP delivery demonstration program for men who have sex with men and female sex workers in Morocco. AIDS Care. 2024;36(4):508–16. doi: 10.1080/09540121.2023.2263679 37795685

[94] Reza-Paul S, Lazarus L, Maiya R, Haldar P, Rewari BB, Venugopal MS, et al. The Ashodaya PrEP project: Lessons and implications for scaling up PrEP from a community-led demonstration project among female sex workers in Mysore, India. Glob Public Health. 2020;15(6):889–904. doi: 10.1080/17441692.2020.1724316 32070217 PMC7261403

[95] Jana S, Ray P, Roy S, Kadam A, Gangakhedkar RR, Rewari BB, et al. Successful integration of HIV pre-exposure prophylaxis into a community-based HIV prevention program for female sex workers in Kolkata, India. Int J STD AIDS. 2021;32(7):638–47. doi: 10.1177/0956462420983992 33596735 PMC8091404

[96] Rao A, Mhlophe H, Pretorius A, Mcingana M, Mcloughlin J, Shipp L, et al. Effect of implementation strategies on pre-exposure prophylaxis persistence among female sex workers in South Africa: An interrupted time series study. Lancet HIV. 2023;10(12):e807–15. doi: 10.1016/S2352-3018(23)00262-X 38040479 PMC10771038

[97] Rao A, Lesko C, Mhlophe H, Rucinski K, Mcingana M, Pretorius A, et al. Longitudinal patterns of initiation, persistence, and cycling on preexposure prophylaxis among female sex workers and adolescent girls and young women in South Africa. AIDS. 2023;37(6):977–86. doi: 10.1097/QAD.0000000000003500 36723509 PMC10079574

[98] Mbotwa CH, Kazaura MR, Moen K, Lichtwarck HO, Leshabari MT, Metta E, et al. Effect of an mHealth intervention on retention in HIV pre-exposure prophylaxis services among female sex workers: Preliminary evidence of the use of the Jichunge app in Dar es Salaam, Tanzania. Digit Health. 2023;9. doi: 10.1177/20552076231170507 37113256 PMC10126669

[99] Ndenkeh JJN, Bowring AL, Njindam IM, Folem RD, Fako GCH, Ngueguim FG, et al. HIV pre-exposure prophylaxis uptake and continuation among key populations in Cameroon: Lessons learned from the CHAMP program. J Acquir Immune Defic Syndr. 2022;91(1):39–46. doi: 10.1097/QAI.0000000000003012 35536113 PMC9377496

[100] Lichtwarck HO, Mbotwa CH, Kazaura MR, Moen K, Mmbaga EJ. Early disengagement from HIV pre-exposure prophylaxis services and associated factors among female sex workers in Dar es Salaam, Tanzania: A socioecological approach. BMJ Glob Health. 2023;8(12):e013662. doi: 10.1136/bmjgh-2023-013662 38154811 PMC10759139

[101] Mbotwa CH, Kazaura MR, Moen K, Sudfeld CR, Metta E, Leshabari MT, et al. Trial of an mHealth intervention to improve HIV prophylaxis for female sex workers, United Republic of Tanzania. Bull World Health Organ. 2024;102(12):852–60. doi: 10.2471/BLT.24.291516 39611190 PMC11601177

[102] Mubezi S, Malamba SS, Rwibasira GN, Uwineza J, Kayisinga J de D, Remera E, et al. Factors associated with retention and adherence on pre-exposure prophylaxis among men who have sex with men in Kigali, Rwanda. PLOS Glob Public Health. 2024;4(12):e0004063. doi: 10.1371/journal.pgph.0004063 39739715 PMC11687698

[103] Bero B, Zidana C, Showa SP. PrEP retention predictors among key populations in urban areas of Zimbabwe - A machine learning approach. Afr J AIDS Res. 2023;22(3):157–64. doi: 10.2989/16085906.2023.2237002 37905425

[104] Busza J, Phillips AN, Mushati P, Chiyaka T, Magutshwa S, Musemburi S, et al. Understanding early uptake of PrEP by female sex workers in Zimbabwe. AIDS Care. 2021;33(6):729–35. doi: 10.1080/09540121.2020.1832192 33043688

[105] Mboup A, Béhanzin L, Guédou FA, Geraldo N, Goma-Matsétsé E, Giguère K, et al. Early antiretroviral therapy and daily pre-exposure prophylaxis for HIV prevention among female sex workers in Cotonou, Benin: A prospective observational demonstration study. J Int AIDS Soc. 2018;21(11):e25208. doi: 10.1002/jia2.25208 31291057 PMC6287093

[106] Mboup A, Béhanzin L, Guédou F, Giguère K, Geraldo N, Zannou DM, et al. Comparison of adherence measurement tools used in a pre-exposure prophylaxis demonstration study among female sex workers in Benin. Medicine (Baltimore). 2020;99(21):e20063. doi: 10.1097/MD.0000000000020063 32481273 PMC7249870

[107] Franks J, Teasdale C, Olsen H, Wang C, Mushimebele N, Tenda Mazala R, et al. PrEP for key populations: Results from the first PrEP demonstration project in the Democratic Republic of the Congo. AIDS Care. 2022;34(3):359–62. doi: 10.1080/09540121.2021.1969332 34495772 PMC10627785

[108] Rugira E, Biracyaza E, Umubyeyi A. Uptake and persistence on HIV pre-exposure prophylaxis among female sex workers and men having sex with men in Kigali, Rwanda: A retrospective cross-sectional study design. Patient Prefer Adherence. 2023;17:2353–64. doi: 10.2147/PPA.S427021 37790862 PMC10542111

[109] Bhattacharjee P, McClarty L, Isac S, Kimani J, Emmanuel F, Kabuti R, et al. Applying the effective programme coverage framework to assess gaps in HIV prevention programmes for female sex workers and men who have sex with men in Nairobi, Kenya: Findings from an expanded Polling Booth Survey. J Int AIDS Soc. 2024;27 Suppl 2(Suppl 2):e26240. doi: 10.1002/jia2.26240 38982888 PMC11233849

[110] Nalukwago GK, Isunju JB, Muwonge T, Katairo T, Bunani N, Semitala F, et al. Adherence to oral HIV pre-exposure prophylaxis among female sex workers in Kampala, Uganda. Afr Health Sci. 2021;21(3):1048–58. doi: 10.4314/ahs.v21i3.12 35222566 PMC8843287

[111] Nakiganda LJ, Grulich AE, Poynten IM, Serwadda D, Bazaale JM, Jin J, et al. Self-reported and pill count measures of adherence to oral HIV PrEP among female sex workers living in South-Western Uganda. PLoS One. 2022;17(11):e0277226. doi: 10.1371/journal.pone.0277226 36355810 PMC9648770

[112] Matambanadzo P, Busza J, Mafaune H, Chinyanganya L, Machingura F, Ncube G, et al. “It went through the roof”: an observation study exploring the rise in PrEP uptake among Zimbabwean female sex workers in response to adaptations during Covid-19. J Int AIDS Soc. 2021;24 Suppl 6(Suppl 6):e25813. doi: 10.1002/jia2.25813 34713613 PMC8554216

[113] Mboup A, Diabaté S, Béhanzin L, Guédou FA, Zannou DM, Kêkê RK, et al. Determinants of HIV preexposure prophylaxis adherence among female sex workers in a demonstration study in Cotonou, Benin: A study of behavioral and demographic factors. Sex Transm Dis. 2021;48(8):565–71. doi: 10.1097/OLQ.0000000000001373 33448731 PMC8284381

[114] Nhamo D, Duma SE, Ojewole EB, Chibanda D, Cowan FM. Factors motivating female sex workers to initiate pre- exposure prophylaxis for HIV prevention in Zimbabwe. PLoS One. 2022;17(7):e0264470. doi: 10.1371/journal.pone.0264470 35797351 PMC9262226

[115] Mapetla KS, Mokgatle MM, Oguntibeju OO. Barriers and facilitators influencing the uptake and acceptability of HIV pre-exposure prophylaxis amongst commercial female sex workers in Tshwane district, Gauteng province: A descriptive study. Int J Innov Res Sci Stud. 2023;6(4):753–61. doi: 10.53894/ijirss.v6i4.1952

[116] Makhakhe NF, MeyerWeitz A, Sliep Y. Motivating factors associated with oral pre-exposure prophylaxis use among female sex workers in South Africa. J Health Psychol. 2022;27(12):2820–33. doi: 10.1177/13591053211072674 34991407

[117] Shipp LM, Ryan S, Comins CA, Mcingana M, Mulumba N, Guddera V, et al. PrEP discontinuation, cycling, and risk: Understanding the dynamic nature of PrEP use among female sex workers in South Africa. PLoS One. 2024;19(9):e0310489. doi: 10.1371/journal.pone.0310489 39325776 PMC11426525

[118] Ddaaki W, Strömdahl S, Yeh PT, Rosen JG, Jackson J, Nakyanjo N, et al. Qualitative assessment of barriers and facilitators of PrEP use before and after rollout of a PrEP program for priority populations in south-central Uganda. AIDS Behav. 2021;25(11):3547–62. doi: 10.1007/s10461-021-03360-3 34240317 PMC8603943

[119] Mujugira A, Nakyanzi A, Kasiita V, Kamusiime B, Nalukwago GK, Nalumansi A, et al. HIV self-testing and oral pre-exposure prophylaxis are empowering for sex workers and their intimate partners: A qualitative study in Uganda. J Int AIDS Soc. 2021;24(9):e25782. doi: 10.1002/jia2.25782 34473405 PMC8412089

[120] Sensoy Bahar O, Nabunya P, Nabayinda J, Witte SS, Kiyingi J, Mayo-Wilson LJ, et al. “I expected little, although I learned a lot”: perceived benefits of participating in HIV risk reduction sessions among women engaged in sex work in Uganda. BMC Womens Health. 2022;22(1):162.doi: 10.1186/s12905-022-01759-1 35562821 PMC9100307

[121] Stoebenau K, Muchanga G, Ahmad SS-O, Bwalya C, Mwale M, Toussaint S, et al. Barriers and facilitators to uptake and persistence on prep among key populations in Southern Province, Zambia: A thematic analysis. BMC Public Health. 2024;24(1):1617. doi: 10.1186/s12889-024-19152-y 38886691 PMC11184712

[122] Amogne MD, Sanders EJ, Belihu WB, Sundewall J, Agardh A. Condom failure and pre-exposure prophylaxis use experience among female sex workers in Ethiopia: A qualitative study. BMC Public Health. 2022;22(1):1079. doi: 10.1186/s12889-022-13468-3 35641959 PMC9158269

[123] Churu M, Kulemba K, Kapesa A, Kajura A, Wilfred D, Basinda N, et al. Factors associated with adherence to oral HIV pre-exposure prophylaxis among female sex workers in the Mara Region, Tanzania: A 2022 cross-sectional study. Med (Baltimore). 2023;102(36):e34824. doi: 10.1097/MD.0000000000034824 37682179 PMC10489305

[124] Leis M, McDermott M, Koziarz A, Szadkowski L, Kariri A, Beattie TS, et al. Intimate partner and client-perpetrated violence are associated with reduced HIV pre-exposure prophylaxis (PrEP) uptake, depression and generalized anxiety in a cross-sectional study of female sex workers from Nairobi, Kenya. J Int AIDS Soc. 2021;24 Suppl 2(Suppl 2):e25711. doi: 10.1002/jia2.25711 34164924 PMC8222843

[125] Nakiganda LJ, Bavinton BR, Poynten IM, Serwadda D, Bazaale JM, Grulich AE. Sexual pleasure and HIV-related worry in female sex workers on oral pre-exposure prophylaxis in south-western Uganda. Sex Health. 2024;21:SH23056. doi: 10.1071/SH23056 38267368

[126] Tolley EE, Bula A, Chitukuta M, Ndimande-Khoza N, Etima J, Namey E, et al. Preferences for oral and injectable PrEP among qualitative sub-study participants in HPTN 084. PLoS One. 2024;19(10):e0309811. doi: 10.1371/journal.pone.0309811 39441777 PMC11498703

[127] Busza J, Chiyaka T, Musemburi S, Fearon E, Davey C, Chabata S, et al. Enhancing national prevention and treatment services for sex workers in Zimbabwe: A process evaluation of the SAPPH-IRe trial. Health Policy Plan. 2019;34(5):337–45. doi: 10.1093/heapol/czz037 31157368

[128] Pelaez D, Weicker NP, Glick J, Mesenburg JV, Wilson A, Kirkpatrick H, et al. The PEARL study: A prospective two-group pilot PrEP promotion intervention for cisgender female sex workers living in Baltimore, MD, U.S. AIDS Care. 2021;33(sup1):1–10. doi: 10.1080/09540121.2021.1889954 33627006 PMC7983056

[129] Manguro GO, Musau AM, Were DK, Tengah S, Wakhutu B, Reed J, et al. Increased condom use among key populations using oral PrEP in Kenya: results from large scale programmatic surveillance. BMC Public Health. 2022;22(1):304. doi: 10.1186/s12889-022-12639-6 35164707 PMC8842980

[130] Giguere R, Frasca T, Dolezal C, Febo I, Cranston RD, Mayer K, et al. Acceptability of three novel HIV prevention methods among young male and transgender female sex workers in Puerto Rico. AIDS Behav. 2016;20(10):2192–202. doi: 10.1007/s10461-016-1387-9 27048236 PMC5448554

[131] Roberts DA, Hawes SE, Bousso Bao MD, Ndiaye AJ, Gueye D, Raugi DN, et al. Trends in reported sexual behavior and Y-chromosomal DNA detection among female sex workers in the Senegal preexposure prophylaxis demonstration project. Sex Transm Dis. 2020;47(5):314–20. doi: 10.1097/OLQ.0000000000001175 32187172 PMC7213512

[132] Atkins K, Kan L, Musau A, Reed J, Were D, Mohan D. Adaptation and psychometric evaluation of a scale to measure oral pre-exposure prophylaxis-related stigma among key and vulnerable populations in Kenya. J Int AIDS Soc. 2022;25 Suppl 1(Suppl 1):e25929. doi: 10.1002/jia2.25929 35818870 PMC9274213

[133] Kavanagh MM, Agbla SC, Joy M, Aneja K, Pillinger M, Case A. Law, criminalisation and HIV in the world: have countries that criminalise achieved more or less successful pandemic response? BMJ Global Health. 2021;6(8). doi: 10.1136/bmjgh-2021-006315 10.1136/bmjgh-2021-006315 PMC8330576 34341021

[134] Lyons CE, Schwartz SR, Murray SM, Shannon K, Diouf D, Mothopeng T, et al. The role of sex work laws and stigmas in increasing HIV risks among sex workers. Nat Commun. 2020;11(1):773. doi: 10.1038/s41467-020-14593-6 32071298 PMC7028952

[135] Shannon K, Strathdee SA, Goldenberg SM, Duff P, Mwangi P, Rusakova M, et al. Global epidemiology of HIV among female sex workers: Influence of structural determinants. Lancet. 2015;385(9962):55–71. doi: 10.1016/S0140-6736(14)60931-4 25059947 PMC4297548

[136] Makhakhe NF, Khumalo G. Developing a female sex worker-led program to improve the uptake of oral pre-exposure prophylaxis in South Africa: An intervention mapping study. Int J Environ Res Public Health. 2025;22(12). doi: 10.3390/ijerph22121862 PMC12732573 41464495

[137] Rock A, Shoen M, Shipp L, Mulumba N, Ryan S, Makama S, et al. “It would have been easier for me to give up if I were alone”: How a pilot individualized case management intervention influenced female sex workers’ oral HIV pre-exposure prophylaxis use in eThekwini, South Africa. BMC Public Health. 2026;26(1):884. doi: 10.1186/s12889-026-26375-8 41639823 PMC12983734

[138] Llibre JM, Iniesta C, Tittle V, Clement M, Rivero Á, Armenteros-Yeguas I. Closing the PrEP access gap in Europe: A strategic framework for equity and innovation among difficult-to-reach populations. J Antimicrob Chemother. 2026;81(3):dkag052. doi: 10.1093/jac/dkag052 41684246

[139] Li F, Xie C, Xiang F. Impact of mHealth on enhancing pre-exposure prophylaxis adherence and strengthening the HIV prevention cascade among key populations: A systematic review and meta-analysis. Front Public Health. 2025;13:1600773. doi: 10.3389/fpubh.2025.1600773 40642253 PMC12240955

[140] Pfau B, Saravis A, Cox SN, Wu L, Wittenauer R, Callen E, et al. User preferences on long-acting pre-exposure prophylaxis for HIV prevention in Eastern and Southern Africa: A scoping review. BMC Public Health. 2025;25(1):2361. doi: 10.1186/s12889-025-23529-y 40611019 PMC12225200

[141] O Murchu E, Marshall L, Teljeur C, Harrington P, Hayes C, Moran P, et al. Oral pre-exposure prophylaxis (PrEP) to prevent HIV: A systematic review and meta-analysis of clinical effectiveness, safety, adherence and risk compensation in all populations. BMJ Open. 2022;12(5):e048478. doi: 10.1136/bmjopen-2020-048478 35545381 PMC9096492

[142] Ngwoke I, Adedire AM, Danladi NP, Eze CC, Dada OK, Okesanya OJ. Barriers and facilitators of oral PrEP uptake against HIV Among selected priority populations: A global perspective. Curr HIV/AIDS Rep. 2026;23(1):14. doi: 10.1007/s11904-026-00783-2 41910679 PMC13035875

