# Supplementary material for: Implementing HIV pre-exposure prophylaxis (PrEP) among cisgender female sex workers: A worldwide scoping review to guide the future research agenda for HIV prevention
Source: PLOS Glob Public Health. 2026 Nov 13;6(11):e0007582. doi: 10.1371/journal.pgph.0007582 (PMC13766235; doi:10.1371/journal.pgph.0007582)
Supplement: Supplementary file 2 [file pgph.0007582.s003.docx]

**S3 Table. PrEP knowledge. [Scoping review on PrEP among female sex workers - until end 2024]**

*Classified by UNAIDS region, type of data, country and year of data collection*

| **Country** | **Years of data collection** | **PrEP availability countrywide at the time of the study** | **Type of data collection for PrEP knowledge*** | **Place of recruitment** | **Population size: FSW only** | **PrEP knowledge**  *(%: ever heard/aware of PrEP)* | **First author, Publication’s year** |
| --- | --- | --- | --- | --- | --- | --- | --- |
| **Eastern and Southern Africa** | | | | | | | |
| Ethiopia | 2022 | Yes | Quanti (IQ) | FSWs registered in two HIV NGOs | n=396 (only HIV-) | 98%  Good knowledge: 51% | Ademe, 2023 |
| Ethiopia | 2022 | Yes | Quanti (IQ) | FSWs registered in the FSW list of the city | n=334 (only HIV-) | 27% | Shibesh, 2023 |
| Ethiopia | 2022 | Yes | Quanti (IQ) | FSWs visiting drop-in centres | n=358 (only HIV-) | 82% | Berhe, 2024 |
| Malawi | 2017 | Very beginning | Quanti (IQ) | FSWs recruited in sex work venues | n=150 (only HIV-) | 21% | Lancaster, 2020 |
| Rwanda | 2019;  2020;  2021;  2022 | Through 11 primary-care health facilities  Initiation: Not clear Retention/Adherence: Not clear | Quanti (PD) | FSWs visiting primary-care health facilities | n=1343 (only HIV-) | 62% | Munyaneza, 2024 |
| South Africa | 2017, 2018 | Yes | Quanti (IQ) | FSWs visiting NDoH clinic visits | n=203 (of whom 187 HIV-) | 86% | Pillay, 2020 |
| South Africa | 2020 | Yes | Quanti (IQ) | FSWs selected through a respondent driven sampling with seeds identified in bars and shebeens | n=199 | 51% | Little, 2022 |
| Tanzania | 2015, 2016 | No | Quanti (IQ) | FSWs recruited in sex work venues | n=293 (only HIV-) | 8% | Beckham, 2022 |
| Tanzania | 2018, 2019, 2020 | Very beginning (since 2018) in demonstration and research projects | Quanti (IQ) | FSWs selected through a respondent driven sampling | n=700 (only HIV-) at baseline, n=562 at 12 months | 67% at baseline  97% at 12 months | Faini, 2022 |
| Tanzania | 2021, 2022, 2023 | Yes | Quanti (IQ) | FSWs selected through a respondent driven sampling and included in a quasi-experimental trial evaluating the effectiveness of a mHealth intervention for PrEP roll-out | n=313 (only HIV-) | 37% at baseline | Mikomangwa, 2024 |
| Uganda | 2017, 2018 | Very beginning | Quanti (IQ) | FSWs selected using a snowball sampling | n=56 (only HIV-) | 45% | Muwonge, 2020 |
| Uganda | 2019 | Yes | Quanti (IQ) | FSWs recruited in sex work venues | n=60 (only HIV-) | 33.3% | Lunkuse, 2022 |
| Uganda | 2023 | Yes | Quanti (IQ) | FSWs recruited in sex work venues and through a respondent driven sampling | n=273 (only HIV-) | 100% | Bongomin, 2023 |
| Zimbabwe | 2016 | No, but PrEP offered through the SAPPH-IRe trial nested in the HIV Sisters programme for FSW | Quanti (IQ) | FSWs selected through a respondent driven sampling | N=611 (only HIV-) | 60.9% | Fearon, 2019 |
| Zimbabwe | 2016, 2017 | Very beginning | Quanti (IQ) | FSWs selected using a snowball sampling | n=131 (only HIV-) | 54% (35% considered with sufficient knowledge) | Mudzviti, 2020 |
| Zimbabwe | 2017, 2018, 2019 | No, but study part of the DREAMS demonstration project | Quanti (IQ) | FSWs selected through a respondent driven sampling | n=963 women who sell sex (only HIV-) in 2017, n=524 in 2018, n=538 in 2019 (only HIV-)  Note: include 66% of women who considered themselves as FSW | 50.3% in 2017  95.7% by 2019 | Hensen, 2021 |
| Kenya | 2016, 2017 | Very beginning | Quali (IDI) | FSWs selected using a snowball sampling | n=45 | Low awareness  - Confusion between PrEP and PEP | Bazzi, 2019 |
| South Africa | 2014 | No | Quali (FGD) | FSWs recruited by peer educators through work/social networks | n=69 in 8 FGD | Low awareness  - Initial understanding of PrEP by equating it with contraception | Eakle, 2018 |
| South Africa | 2015, 2016, 2017 | No, but study part of a demonstration project | Quali (IDI) | FSWs visiting HIV clinics | n=18 at baseline, n=10 2^nd^ round, n=6 3^rd^ round | Low awareness  - Disbelief as during decades messages were that condoms was the only way to prevent HIV transmission  - Confusion with ARV for HIV+ | Eakle, 2019 |
| South Africa | 2018 | Yes | Quali (IDI, FGD) | FSWs selected using a snowball sampling | IDI: n=17  FGD: n=9 in 2 groups | Confusion between PrEP and ARV treatment | Makhakhe, 2022 |
| **Western and Central Africa** | | | | | | | |
| Nigeria | 2020 | Yes | Quanti (IQ) | FSWs visiting a community-based clinic | n=344 (only HIV-) | 77% | Omo-Emmanuel, 2023 |
| Nigeria | 2020 | Yes | Quanti (IQ, PD) | FSWs recruited in sex work venues | n=1458 | 20% | Olakunde, 2024 |
| **Middle East and North Africa** | | | | | | | |
| **Western and central Europe and North America** | | | | | | | |
| USA, Maryland | Missing | Yes | Quanti (IQ) | FSWs recruited in two mobile health services | n=60 | 33% | Peitzmeier, 2017 |
| USA, Rhode Island | 2014, 2015, 2016, 2017 | Not clear | Quanti (IQ) | FSWs recruited in a STI clinic | n=34 | 6.3% | Sosnowy, 2020 |
| USA, Maryland | 2016, 2017 | Very beginning | Quanti (IQ) | FSWs recruited in sex work venues | n=232 (only HIV-) | 21% | Tomko, 2019 |
| Spain | 2022 | Yes | Quanti (IQ) | FSWs recruited in sex work venues | n=102 | 9.8% | Vazquez Guillamet, 2024 |
| USA, Maryland | 2017, 2018 | Only for MSM and trans women | Quali (CF, FGD) | FSWs selected from community organizations and health providers | CF: n=6  FGD: n=10 in 2 groups | Low awareness  - Misconceptions: “PrEP = gay man’s pil”’ | Glick, 2022 |
| USA, Massachusetts | 2021, 2022 | Yes | Quali (IDI) | FSWs visiting two bridge clinics | n=25 (who self-reported HIV-) | High level of awareness, through social networks and local harm reduction, methadone or bridge clinic services. Yet, clinical priorities (treatment of substance use disorders) and women’s survival needs (being unhoused) can be barriers to PrEP education. | Harris, 2024 |
| **Eastern Europe and Central Asia** | | | | | | | |
| Kazakhstan | 2021 | Very beginning, only in AIDS centers | Quali (IDI, FGD) | FSWs visiting HIV NGOs | Only FSWs also being drug users  ID: n=30  FGD: n=18 in 4 groups | (-qual) confusion between PrEP and HIV treatment | McCrimmon, 2023 |
| **Asia and Pacific** | | | | | | | |
| Cambodia | 2019 | No | Quanti (IQ) | FSWs selected through a respondent driven sampling | n=1003 | 31.8%  (+quanti) ever married | Garg, 2022 |
| China | 2009, 2010 | No | Quanti (IQ) | FSWs selected using a snowball sampling | n=1611 | 16.5% | Peng, 2012 |
| China | 2009, 2010 | No | Quanti (IQ) | FSWs selected through a convenience sampling with the assistance of local social and health-care institutions and snowball  sampling | n=405 (who self-reported HIV- or unknown HIV status) | 15.1% | Ye, 2014 |
| China | 2012 | No | Quanti (IQ) | FSWs recruited in sex work venues | n=1466 | 10.2% | Poon, 2019 |
| **Latin America and the Caribbean** | | | | | | | |
| Jamaica | Missing | No | Quanti (IQ) | FSWs selected through word-of-mouth and snowball  sampling | n=101 | 30.1% | Logie, 2021 |

*IDI: in-depth interview; FGD: focus-group discussion; IQ: individual questionnaire; CF: community forum*

** if several types of data were collected, only the type(s) of data collection with specific results on PrEP knowledge are indicated*
