# Supplementary material for: Implementing HIV pre-exposure prophylaxis (PrEP) among cisgender female sex workers: A worldwide scoping review to guide the future research agenda for HIV prevention
Source: PLOS Glob Public Health. 2026 Nov 13;6(11):e0007582. doi: 10.1371/journal.pgph.0007582 (PMC13766235; doi:10.1371/journal.pgph.0007582)
Supplement: Supplementary file 3 [file pgph.0007582.s004.docx]

**S4 Table. PrEP a priori acceptability [Scoping review on PrEP among female sex workers - until end 2024]**

*Classified by UNAIDS region, type of data, country and year of data collection*

| **Country** | **Year of data collection** | **PrEP availability countrywide at the time of the study** | **Type of data collection for PrEP a priori acceptability / perceptions*** | **Place of recruitment** | **Population size: FSW only** | **PrEP a priori acceptability**  **[Type PrEP]**  *(%: interest/willingness to take PrEP)* | **First author, Publication’s year** |
| --- | --- | --- | --- | --- | --- | --- | --- |
| **Eastern and Southern Africa** | | | | | | | |
| Ethiopia | 2022 | Yes | Quanti (IQ) | FSWs visiting drop-in centres | n=358 (only HIV-) | [Global PrEP]  68%  (+quan) drop-in center not far from their home, PrEP knowledge  (-quan cited) fear of stigma, side effects, lack of information, preference for condoms | Berhe, 2024 |
| Rwanda | 2019;  2020;  2021;  2022 | Through 11 primary-care health facilities  Initiation: Not clear Retention/Adherence: Not clear | Quanti (PD) | FSWs visiting primary-care health facilities | n=1343 (only HIV-) | [Oral]  93% | Munyaneza, 2024 |
| South Africa | 2015 | No | Quanti (IQ) | FSWs selected through a respondent driven sampling | n=203 | [Oral, Gel, Injection, Vaginal rings]  (+quan) preferences for injectable products compared to oral PrEP and microbicide gels | Quaife, 2018 |
| South Africa | 2020 | Yes | Quanti (IQ) | FSWs selected through a respondent driven sampling with seeds identified in bars and shebeens | n=199 | [Implant]  86%  (+quan) long acting but dissolvable, does not require surgical removal, desire to receive it from a family planning provider, prefer a product that would provide dual protection against HIV and unintended pregnancies | Little, 2022 |
| Tanzania | 2018, 2019, 2020 | Very beginning (since 2018) in demonstration and research projects | Quanti (IQ) | FSWs selected through a respondent driven sampling | n=700 (only HIV-) at baseline, n=562 at 12 months | [Oral]  98% at baseline, 96% at M12 | Faini, 2022 |
| Tanzania | 2022 | Not clear  Initiation: Not clear  Retention/Adherence: Not clear | Quanti (IQ) | FSWs recruited through a respondent-driven sampling | n=428 (HIV-) | [Oral]  80% | Martin, 2023 |
| Uganda | 2017, 2018 | Very beginning | Quanti (IQ) | FSWs selected using snowball sampling | n=56 (only HIV-) | [Oral]  86%  (-quan cited) fear of stigma, pill burden, happy with condoms | Muwonge, 2020 |
| Uganda | 2019 | Yes | Quanti (IQ) | FSWs recruited in sex work venues | n=60 (only HIV-) | [Oral]  (-quan: cited) high pill burden  [Injection]  (-quan: cited) fear of side-effects | Lunkuse, 2022 |
| Uganda | 2019, 2020 | Yes | Quanti (IQ) | FSWs enrolled in a trial evaluating a combination intervention  adding microfinance components to traditional HIV  risk reduction | n=322 (only HIV-) | [Oral]  91%  (+quan) engage in sex work recently, social support from family | Witte, 2022 |
| Zimbabwe | 2016, 2017 | Very beginning | Quanti (IQ) | FSWs selected using snowball sampling | n=131 (only HIV-) | [Oral]  78%  (+quan) higher age, high knowledge, unprotected sexual intercourse in the last 3 months  (-quanti) cost of the PrEP | Mudzviti, 2020 |
| Zambia, Uganda | 2016 | No | Quanti (IQ) | FSWs recruited by peer educators | n=633 in Zambia  n=749 in Uganda  (only HIV-) | [Oral]  91% Zambia; 66% Uganda  [Injection]  88% Zambia; 77% Uganda  [Gel]  49% Zambia; 51% Uganda  [Vaginal rings]  40% Zambia; 21% Uganda | Ortblad, 2018 |
| Malawi | Not clear | Very beginning | Mix (IQ, FGD, IDI) | FSWs recruited in sex work venues | IQ: n=150 (only HIV-)  FGD: n=44 in 8 groups  IDI : n=5 | [Oral]  96% very willing; 1% probably willing  (–qual) other competing responsibilities (chores, work, rest) making it challenging to spend time in clinic to receive PrEP | Lancaster, 2020 |
| Tanzania | 2015, 2016, 2017 | No | Mix (IQ, IDI) | FSWs recruited in sex work venues | IQ: n=293 (HIV- only)  FGD: n=20 in 2 groups  IDI: n=10 (HIV- only) | [Global PrEP: Oral, Injection, Vaginal rings]  58%  (+quan) having had STI symptoms or diagnosis in the past 6m, having higher social cohesion scores  (+qual) backup to condom use in case the client refuses to use one, uses force, becomes violent, offers more money for condomless sex or if the broken condom  88% preference for long-acting PrEP  [Oral]  (+qual) familiarity with pills use  (-qual) daily use, easy to forget to take - especially if alcohol use at work, fear of stigma from clients/partners (if confusion between PrEP and HIV treatment), fear of interaction with alcohol  [Injection]  (+qual) less frequent to use, easy to hide from partners and clients, familiarity with injections (family planning), belief in power and efficacy, fits with spontaneous short-term or long-term mobility  (-qual) fear of side-effects (as inability to clear it quickly from the body), discomfort with needles  [Vaginal rings]  (+qual) less frequent to use  (-qual) placement in vagina, fear of feeling it (herselves or clients), fear of displacements during sex, fear of infertility/changes to menstruation | Beckham, 2022 |
| Kenya | 2014, 2015 | No | Quali (IDI) | FSWs recruited via convenience sampling in bars and nightclubs | n=21 (self-reported HIV- status) | [Oral]  (+qual) HIV protection in situation of inconsistence condom use, expression of self-love and self-care  (-qual) concerns about potential side-effects of PrEP | Restar, 2017 |
| Kenya | 2016, 2017 | Very beginning | Quali (IDI) | FSWs selected using a snowball sampling | n=45 | [Oral]  (+qual) additional HIV protection in case of condomless sex due to violence, a lapse after alcohol use, or for higher pay | Bazzi, 2019 |
| Kenya | 2019-2020 | Yes | Quali (FGD, observations) | FSWs visiting health facilities | FGD: n=28 in 2 groups  Observations: n=9 | [Microarray patch]  (observations) difficulties regarding usability: cleaning of the application site, pressing down firmly  (+qual) no risk to forget it  (-qual) size if the microarray patch that could be smaller | Gachigua, 2023 |
| Malawi | 2016 | Not clear | Quali (FGD) | FSWs recruited in sex work venues | n=44 in 8 groups (only HIV-) | [Oral]  (+qual) in love for one’s life, anticipating negative economic effects associated with HIV acquisition  (-qual) unpredictable daily lives, fear of stigma, fear of side-effects, alcohol | Shea, 2019 |
| South Africa | 2011, 2012 | No | Quali (FGD) | FSWs visiting health facilities | n=33 in 3 groups | [Oral, Gel, Injection]  (+qual) PrEP can help earn more money  (-qual) PrEP does not protect against other STI, fear of side effects  [Oral]  (-qual) fear of low adherence especially when alcohol use  [Injectable]  (+qual) preference as one dose would last for a prolonged period of time  [Gel]  (-qual) afraid to forget to apply it | Mack, 2014 |
| South Africa | 2014 | No | Quali (FGD) | FSWs recruited by peer educators through work/social networks | n=69 in 8 groups | [Oral]  (+qual) HIV protection in case of condom burst, sexual violence or to protect from regular partner who can have sex with other women (but not to replace condom as PrEP does not protect against unwanted pregnancies or other STIs)  (-qual) fear of low adherence (not motivate to take a daily pill, or pill forget if alcohol or drug use), fear of side-effects | Eakle, 2018 |
| South Africa | 2019 | Yes | Quali (IDI) | FSWs recruited from shebeens and taverns with a snowball sampling | n=8 (among more individuals) | [Oral]  (-qual) fear of stigma, fear of poor adherence  [Implant]  (+qual) outweigh the burden of daily oral PrEP  (-qual) fear of side-effects (but would be able to tolerate it if high degree of protection and effective counselling on side effects) | Brown, 2022 |
| South Africa | 2021 | Yes | Quali (FGD) | FSWs recruited through community outreach | FGD: n=10 in 2 groups | [Vaginal Davirapine ring]  (-qual) fear of side effects, and that the ring is invasive or might feel discomfort or pain while inserting it, concerns about the impact of everyday use, vaginal hygiene, or the possibility of the ring being felt during sexual intercourse  [Long-acting cabotegravir (injection)]  (+qual) familiarity with injectable contraceptive, ensures continuation as no risk to forget to take pills, convenient and discreet to use | Mataboge, 2023 |
| Tanzania | 2021, 2022 | Yes | Quali (IDI) | FSWs recruited through a  peer chain referral method and eligible to PrEP | n=40 | [Oral]  (+quali) feeling of freedom from HIV fear and positive effect on wellbeing and autonomy, PrEP as empowering and a way to prioritize health and test for HIV more often  (-qual) fear of stigma associated with PrEP, as PrEP can inadvertently reveal engagement in sex work, and some were afraid that others would assume they are HIV-positive as PrEP looks like ART; burden of daily PrEP use especially when women have unpredictable schedules and can spontaneously travel, fear of low retention (taking time to go to clinical visits away from children and from sex work) | Lichtwarck, 2024 |
| Tanzania | 2017-2018 | Yes | Quali (IDI) | FSWs recruited in sex work venues and included in a cohort | IDI: n=20 | [General]  (+qual) PrEP protect themselves in their work, especially in the case of a client intentionally breaking a condom  (-qual) why take pills why not sick  [Oral]  (+qual)  (-qual) daily burden of remembering to take a pill, especially in the reality of heavy alcohol and with unexpected travelling, fear of side-effects, fear of violence, stigma or refusal to pay for sex if pills discovered by a client, fear that PrEP pills considered by clients are antiretroviral treatments  [Long-acting]  (+qual) more logistically feasible (relief from the stress of worrying about missed pills), more discrete (reduce stigma)  (-qual) fear of side-effects, fear of needles, skepticism about what would be injected | Mantsios, 2022 |
| Uganda | 2017, 2018, 2019 | Very beginning | Quali (IDI) | FSWs enrolled in a longitudinal cohort of women at high risk of HIV and STIs, recruited in sex work venues | n=30 at several time points (HIV- only) | [Oral]  (+qual) PrEP presented by trusted healthcare staff, PrEP allow to protect against HIV during sex work but also with regular partners, PrEP is not reliant on others and is not a preventive method that need to be used at the time of sexual intercourse | Kawuma, 2021 |
| Zambia | 2021 | Yes | Quali (FGD) | FSWs identified by health centres staff | n=43 FSW | [Global PrEP]  (+quali) to protect their well-being and stay HIV-negative, benefits in case men do not use or incorrectly use condoms  [Ring and CAB]  (+quali) not having to remember daily dosing (especially when women do not have fixed home or who travel frequently), discreet use, longer duration  [injectable CAB]  (+qual) protective for vaginal and anal sex  (-qual) non-aligned injection schedules every two months with depot medroxyprohesterone acetate contraception every three months  [Ring]  (-quali) fear of the PrEP ring become dislodges, pushed through the cervix (especially in case of ‘tough sex’ or partner has large penis). | Evens, 2024 |
| South Africa  Uganda | Not clear | Yes | Quali (FGD, observations) | FSWs known by program sites and clinics | FGD: n=12 in 2 groups  Observations: n=13 | [Microarray patch]  (+qual) discreet, painless, decrease the burden of daily oral pills, possibility of self-administration  (-qual) concerns regarding potential side effects | Ismail, 2023 |
| **Western and Central Africa** | | | | | | | |
| Ghana | 2020 | Very beginning (since 2020) | Quanti (IQ) | FSW recruited in sex work venues | n=5107 (only HIV-) | [Oral]  54%  (+quan) younger, already been married/cohabiting, having already heard of PrEP, already in couple, already having injected drugs, already screened for STI | Guure, 2022 |
| Nigeria | 2020 | Yes | Quanti (IQ) | FSWs visiting a community-based clinic | n=344 (only HIV-) | [Oral]  8%  (-quan cited) condom use, lack of knowledge, fear of side effect, traditional beliefs associated with HIV infection | Omo-Emmanuel, 2023 |
| Ivory Coast | 2016, 2017 | No | Mix (IQ, FGD, IDI) | IQ: FSW recruited by peer educators or when they visited NGOs’ community clinics  FGD and IDI: FSWs recruited during the outreach activities of peer educators on sex work venues | IQ: n=1000  IDI: n=22  FGD: n=44 in 8 groups | [Oral]  98.6%  (+qual) useful especially with regular partners with who they do not use any condom  (-qual) fear of side-effects, concerns regarding costs, compatibility with pregnancy and adherence | Becquet, 2020 |
| Nigeria | Missing | Yes | Quali (FGD) | Not clear | FGD: n=80 in 8 groups | [Oral]  (-qual) fear of stigmatization from clients as PrEP has the same colour as ARV for HIV infection, high mobility = availability/accessibility of drugs, low priority regarding healthcare | Emmanuel, 2020 |
| **Middle East and North Africa** | | | | | | | |
| Morocco | 2021 | Through ALCS (Association de Lutte Contre le Sida) sexual health clinics  Initiation: Not clear  Retention/Adherence: Not clear | Quali (IDI) | FSWs visiting health centres | n=10 | [Injection]  (+qual) more convenient than daily pills (no risk to forget it)  [Vaginal rings]  (-qual) concerns about putting something into the vagina | Moussa, 2024 |
| **Western and central Europe and North America** | | | | | | | |
| USA, Maryland | Missing | Yes | Quanti (IQ) | FSWs recruited in two mobile health services | n=58 | [Oral]  65%  (+quan) recent experience of client-perpetrated violence  [Vaginal rings]  76%  (+quan) recent experience of client-perpetrated violence | Peitzmeier, 2017 |
| USA, Maryland | 2016, 2017 | Not clear | Quanti (IQ) | FSW recruited in sex work venues | n=236 (only HIV-) | [Injection, Gel, Vaginal rings]  67% interest in at-least one event-driven or long-acting PrEP formulation: preferences for quarterly arm injections (56%) compared to quarterly abdomen injections (29%), monthly vaginal rings (25%), topical gel (25%) and intravenous infusions (20%).  18% universal acceptors, 24% injectable acceptors, 58% non acceptors  (+quan) condomless vaginal sex with clients, injection of drugs, client-perpetrated condom coercion, client-perpetrated sexual violence | Rosen, 2022 |
| USA, Maryland | 2016, 2017 | Very beginning | Quanti (IQ) | FSWs recruited in sex work venues | n=232 (only HIV-) | [Oral]  74%  (+quan) PrEP perception as easy to take, moving to unfamiliar geographical area to sell sex, not having current STI | Tomko, 2019 |
| USA, California | 2017, 2018 | Yes | Quanti (IQ) | FSWs recruited through flyers  and advertisements as well as providers at testing sites, clinics and community-based organizations | n=21 (only HIV-) | [Oral]  95% | Blumenthal, 2021 |
| USA, California | 2019, 2020 | Yes | Quanti (IQ) | FSWs recruited through online dissemination and word of mouth | n=83 (only HIV-) | [Oral]  (+quan) providing others with PrEP knowledge, importance of free care  (-quan) anticipated disapproval or stigmatizing stereotypes from others | Ramos, 2024 |
| Spain | 2022 | Yes | Quanti (IQ) | FSWs recruited in sex work venues | n=102 (only HIV-) | All type of PrEP: 72.5%  (+quan) perception of HIV PrEP efficacy, inconsistent condom use or perception of risk despite consistent condom use  [Oral]: 59.0%  [Injection]: 57.8%  [Vaginal ring]: 22.5% | Vazquez Guillamet, 2024 |
| USA, Maryland | 2016, 2017 | Yes | Quali (FGD) | FSWs recruited through the distribution within a cohort study of street-based FSW and the city health department’s needle exchange program | n=15 in 4 groups (only HIV-) | [Oral]  (-qual) concerns regarding adherence as unpredictable daily life  [Implant]  (-qual) fear of stigma as difficult to hide, fear of side effect  [Injection]  (+qual) outweigh the burden of daily oral pills  (-qual) less control over, and fear of any irreversible side-effects | Footer, 2019 |
| USA, Maryland | 2016, 2017 | Only for MSM and trans women | Quali (FGD, IDI, CM) | FSWs selected from community organizations and health providers | CM: n=6  FGD: n=10 in 2 groups | [Oral]  (-qual) lack of PrEP knowledge, concerns about adherence especially when substance use or because of no daily routine | Glick, 2022 |
| USA, Massachusetts | 2021, 2022 | Yes | Quali (IDI) | FSWs visiting two bridge clinics | n=25 (only self-reported HIV-) | [Injection]  (+qual) overcome barriers to daily oral pill  (-qual) fear of side effects, fear of adherence challenges if needed to have 2-month follow-up | Harris, 2024 |
| **Eastern Europe and Central Asia** | | | | | | | |
| Kazakhstan | 2021 | Very beginning, only in AIDS centers | Quali (IDI, FGD) | FSWs visiting HIV NGOs | Only FSWs also being drug users (and only self-reported HIV-)  ID: n=30  FGD: n=18 in 4 groups | [Oral]  (+qual) to protect their health  (-qual) concerns regarding side effects and regarding daily pills | McCrimmon, 2023 |
| **Asia and Pacific** | | | | | | | |
| Cambodia | 2019 | No | Quanti (IQ) | FSWs selected through a respondent driven sampling | n=1003 | [Oral]  67%  (+quan) previous knowledge of PrEP, no additional income source other than sex work | Garg, 2022 |
| China | 2009, 2010 | No | Quanti (IQ) | FSWs recruited in sex work venues | n=395 | [Oral]  74%  (+quan) higher thwarted belongingness, higher trust in physicians, higher self-efficacy in using PrEP  (-quan) higher stigma belief | Jackson, 2013 |
| China | 2009, 2010 | No | Quanti (IQ) | FSWs selected through a convenience sampling with the assistance of local social and health-care institutions and snowball  sampling | n=405 | [Oral]  86%  (+quan) working in male dominated venues (versus in the streets), higher outcome, poor family relationship, good HIV knowledge, not realizing HIV risk from unfamiliar clients, not being forced to use condoms by the gatekeeper, having already used drugs to prevent sexually transmitted infections, consistent use of condoms  (-quan/cited) fear of side-effects, no perception of HIV risk, concerns about objections from family, concerns about discrimination by others | Ye, 2014 |
| China | 2009, 2010 | Not clear | Quanti (IQ) | FSWs selected using a snowball sampling | n=1611 | [Oral, Gel]  69%, 72.6% if PrEP free, 80.6% if already lots of PrEP users  (+quan) Han ethnicity, urban residence, knowledge about HIV, STI history, history of preventive medication for STI, interest about HIV/AIDS  (-quant/cited) concerns about the safety, the efficacy and the costs | Peng, 2012 |
| China | 2012 | No | Quanti (IQ) | FSWs recruited in sex work venues | n=1466 | [Oral]  35.5%  (+quan) older age, having regular clients, having been tested for HIV, having greater decision-making involvement in condom use  (+quan/cited) free, no side effect | Poon, 2019 |
| India | 2015 | No | Mix (IQ, FGD, IDI) | IQ: FSWs selected through a respondent driven sampling  FGD and IDI: FSWs recruited using peer educators’ contact lists | IQ: n=427 FSW  FGD: n=38 in 6 groups  IDI: n=35 | [Oral]  95% interest in taking PrEP - 89% they would take PrEP every day  (+qual) safety if condom break or forced sex, or with regular partner; allow to maintain health and protect ability to support themselves as well as their dependents  (-qual/-quant: cited) daily use and long term-adherence, especially when frequent travel and unpredictable work schedules or because of alcohol use, fear of side-effects, fear of stigma | Reza-Paul, 2016 |
| India | 2013, 2014 | No | Quali (FGG, IDI) | FSWs recruited through NGOs, community-based organization and key community gatekeepers | IDI: n=39  FGD: n=14 in 2 groups | [Oral]  (+qual) experience of coercive condom-less sex, way of empowerment to protect against HIV, possibility of financial gains (if condomless sex)  (-qual) fear of stigma of using HIV treatment product, fear of side-effects (physical and regarding reproductive health), fear of low adherence due to alcohol use and no daily routine  [Long-acting PrEP (in general)]  (+qual) when oral PrEP adherence is difficult | Sahay, 2021 |
| Malaysia | Missing | No | Quali (IDI) | FSWs recruited via NGOs that provide outreach and education using digital flyers | n=30 | [Oral]  (+qual) way to proactively prevent HIV, especially when they forget to use condoms while using drugs  (-qual) PrEP cost, fear of low adherence as irregular routine, fear of side effect | Foley, 2024 |
| **Latin America and the Caribbean** | | | | | | | |
| Jamaica | Missing | No | Quanti (IQ) | FSWs recruited through word-of-mouth and snowball  sampling | n=114 (only HIV-) | [Oral]  84.2% | Logie, 2021 |
| Mexico | 2016, 2017 | No | Quanti (IQ) | FSWs recruited in sex work venues ; to be part of a larger trial evaluating a text message-based behavioural HIV prevention intervention | n=271 (only HIV-) | [Oral, Gel, Implant, Injection, Vaginal douche, Vaginal rings]  >95%  Preference for oral pill or vaginal gel (compared to vaginal douche or rings) | Pines, 2019 |
| Mexico | 2016, 2017 | Not clear | Quanti (IQ) | FSWs recruited in sex work venues ; to be part of a larger trial evaluating a text message-based behavioural HIV prevention intervention | n=295 (only HIV-) | [Oral]  (-quan cited) do not have money to pay PrEP, not convince with complete protection of PrEP against HIV, worry to lose clients because of stigma (they’ll assume HIV+ status), healthcare clinics have limited hours of operation | Jain, 2020 |
| Mexico | 2010, 2011, 2012 | No | Mix (IQ, IDI) | FSWs recruited within a cohort | IQ: n=185  IDI: n=28 | [Gel]  89%  (qual+) when condom negotiation is difficult or in case of accidents | Robertson, 2013 |
| Dominican Republic | 2017-2018 | Yes | Quali (IDI) | FSWs recruited in sex work venues and included in a cohort | N=20 | [General]  (+qual) PrEP protect themselves in their work, especially in the case of a client intentionally breaking a condom  (-qual) why take pills why not sick  [Oral]  (+qual)  (-qual) daily burden of remembering to take a pill, especially in the reality of heavy alcohol and with unexpected travelling, fear of side-effects, fear of violence, stigma or refusal to pay for sex if pills discovered by a client, fear that PrEP pills considered by clients are antiretroviral treatments  [Long-acting]  (+qual) more logistically feasible (relief from the stress of worrying about missed pills), more discrete (reduce stigma)  (-qual) fear of side-effects, fear of needles, skepticism about what would be injected | Mantsios, 2022 |
| Peru | Missing | Not clear | Quali (FGD) | FSWs recruited in sex work and other venues | n=15 in 3 groups | [Oral]  (+qual) method of self-care, back-up protection when condoms are forgotten or break, or for casual sex  (-qual) fear of side effects, concerns regarding remembering to take pills for this daily regimen | Galea, 2011 |

*IDI: in-depth interview; FGD: focus-group discussion; IQ: individual questionnaire; CM: consultative meetings*

*(+qual) / (+quan): facilitators / factors positively associated with PrEP a priori acceptability, in qualitative and quantitative studies, respectively*

*(-qual) / (quan-): barriers / factors negatively associated with PrEP a priori acceptability, in qualitative and quantitative studies, respectively*

** if several types of data were collected, only the type(s) of data collection with specific results on PrEP a priori acceptability are indicated*
