# Supplementary material for: Implementing HIV pre-exposure prophylaxis (PrEP) among cisgender female sex workers: A worldwide scoping review to guide the future research agenda for HIV prevention
Source: PLOS Glob Public Health. 2026 Nov 13;6(11):e0007582. doi: 10.1371/journal.pgph.0007582 (PMC13766235; doi:10.1371/journal.pgph.0007582)
Supplement: Supplementary file 4 [file pgph.0007582.s005.docx]

**S5 Table. PrEP effective acceptability [Scoping review on PrEP among female sex workers - until end 2024]**

*Classified by UNAIDS region, type of data, country and year of data collection*

| **Country** | **Year of data collection** | **PrEP delivery**  *including specific information regarding steps for PrEP initiation and support for retention/adherence* | **Type of study and data collection for PrEP effective acceptability*** | **Place of recruitment** | **Population size: FSW only, all HIV-** | **PrEP effective acceptability**  **[Type PrEP/Outcomes]**  *(%: use/retention/adherence)* | **First author, Publication’s year** |
| --- | --- | --- | --- | --- | --- | --- | --- |
| **Eastern and Southern Africa** | | | | | | | |
| Ethiopia | 2022 | In a health center  Initiation: Not clear  Retention/Adherence: Not clear | Quanti (IQ) | FSWs registered in the FSW list of the city | n=334 (only PrEP eligible) | [Oral/use: PrEP uptake]  16%  (+quan) history of STI  (-quan) single, only one parent alive  (-quan cited) lack of information, fear of stigma, lack of trust, transportation issues, worry about side effects | Shibesh, 2023 |
| Ethiopia | 2022 | Through three NGOs that serve integrated HIV services  Initiation: Not clear  Retention/Adherence: Not clear | Quanti (IQ) | FSWs visiting NGOs health services | n=561 (only HIV-) | [Oral/use: current use]  29%  (+quan) duration of living in sex work, level of education above college | Asmare, 2024 |
| Ethiopia | 2022 | By two NGO through a community-based approach  Initiation: Not clear  Retention/Adherence: Not clear | Quanti (IQ) | FSWs registered in two HIV NGOs | n=396 (only HIV-) | [Oral/use: ever used]  46%  (+quan) younger age, higher education level, good knowledge | Ademe, 2023 |
| Kenya | 2017, 2018 | In HIV clinics, antenatal clinics and drop-in centers for key populations  Initiation: Not clear  Retention/adherence: Not clear | Quanti (IQ: baseline, M6, M12) | FSWs recruited in sex venues | n=322 (only HIV-) | [Oral/use: current use]  11.1% | Bien-Gund, 2022 |
| Kenya | 2019 | Through the Kenya Aids Control Project clinics  Initiation: Not clear  Retention/Adherence: Not clear | Quanti (IQ) | FSWs visiting HIV clinics | IQ: n=215 (only HIV-) | [Oral/use: current use]  42.3%  (-quan) client emotional violence  (+quan) client physical violence | Leis, 2021 |
| Kenya | 2019 | In acute care services  Initiation: Not clear  Retention/Adherence: Not clear | Quanti (IQ) | FSWs visiting HIV clinics | n=217 (only HIV-): 101 acute care users and 116 outpatient care only users | [Oral/use: current use]  48.5% of acute care users  49.1% of outpatient care only users | Poxon, 2023 |
| Rwanda | 2019;  2020;  2021;  2022 | Through 11 primary-care health facilities  Initiation: Not clear Retention/Adherence: Not clear | Quanti (PD) | FSWs visiting primary-care health facilities and included in a cohort | n=1343 who initiated PrEP | [Oral/retention]  At M1: 92%  At M3: 90%  At M6: 86%  At M9: 79%  At M12: 76%  (-quan: retention at M1) low HIV risk perception, living with others vs alone, having part-time employment or being students (vs having a full-time job)  (-quan: retention at M12) low HIV risk perception only | Munyaneza, 2024 |
| Rwanda | 2019,  2020 | Through 10 health centers in Kigali  Initiation: Not clear  Retention/Adherence: Not clear | Quanti (PD) | FSWs visiting primary-care health facilities | n=4824 (only HIV-)  n=2219 who initiated PrEP | [Oral/use: PrEP uptake]  45.5%  (+quan) younger age, living alone (compared to with family and roommates)  [Oral/retention: 1 year]  78.50% | Rugira, 2023 |
| Rwanda | 2020  2021 | In five health centres in the Rwanda HIV program.  Initiation: Not clear  Retention/Adherence: Not clear | Quanti (PD) | FSWs visiting healthcare centres and included in a cohort | n=268 who initiated PrEP | [Oral/retention]  At M1: 81%  At M6: 67%  At M12: 53%  (+quan) always using condoms, wanted 2 children or more | Mubezi, 2023 |
| South Africa | 2015, 2016, 2017 | Through the TAPS project in clinics  Initiation: one week after screening & biological assessment  Retention/Adherence: short SMS reminders for clinic visits + positive messages around adherence | Quanti (PD) | FSWs visiting clinics and included in a cohort | n=351 (HIV-)  n=219 who initiated PrEP | [Oral/use: PrEP assessment & PrEP initiation]  PrEP assessment: 69% (241/351)  PrEP initiation: 98% (219/224 eligible)  [Oral/retention: among those on PrEP]  53% at M1, 44% at M3, 30% at M6, 22% at M12  [Oral/adherence: taking PrEP daily]  70-85% | Eakle, 2017 |
| South Africa | 2016, 2017, 2018, 2019, 2020 | Through mobile van health clinics and drop-in center cellness clinics.  Initiation: Not clear  Retention/Adherence: Not clear | Quanti (PD) | FSWs visiting an HIV NGOs and included in a cohort | n=1659 in 2016 to 4446 in 2020 (HIV-)  n=2776 who initiated PrEP | [Oral/use: PrEP initiation]  9% in 2016, 27% in 2020  [Oral/adherence: persistence of PrEP]  53% at M1, 33% at M4, 18% at M7, 9% at M12  (-quan) younger age  -> 13% restarted PrEP with a median of 3 months | Rao, 2022 |
| South Africa | 2016, 2017, 2018, 2019, 2020,  2021 | Through the TB HIV Care NGO.  Initiation: Not clear  Retention/Adherence: several strategies including: i) clinical mentoring for providers, ii) mobile van PrEP provision, iii) SMS PrEP refill reminders, iv) SMS support texts, v) case management approach, vi) loyalty rewards program (incentives in the form of airtime to access online services) | Quanti (PD) | FSWs visiting an HIV NGOs and included in a cohort | n=11020 who initiated PrEP | [Oral/retention]  At 1 month following initiation: 33%  (+quan) SMS support, clinical mentoring  (-quan) loyalty rewards  At 4 months: 18%  (+quan) clinical mentoring  (-quan) loyalty rewards | Rao, 2023 |
| South Africa | 2016, 2017, 2018, 2019, 2020,  2021 | Through the TB HIV Care NGO. Not clear.  Initiation: Not clear  Retention/Adherence: several strategies including: i) clinical mentoring for providers, ii) mobile van PrEP provision, iii) SMS PrEP refill reminders, iv) SMS support texts, v) case management approach, vi) loyalty rewards program (incentives in the form of airtime to access online services) | Quanti (PD) | FSWs visiting an HIV NGOs and included in a cohort | n=65782 (HIV-)  n=12581 who initiated PrEP | [Oral/use]  19%  Increase over time  [Oral/retention: 1 month]  41%  (+ quan) older age  [Oral/Re-initiation: restarting PrEP within 12 months of an initial PrEP prescription, after >2 month gap in PrEP]  9% | Rao, 2023 |
| South Africa | 2020 | Not clear  Initiation: Not clear  Retention/Adherence: Not clear | Quanti (IQ) | FSWs selected through a respondent driven sampling with seeds identified in bars and shebeens | n=101 aware of PrEP | [Oral/use]  24% | Little, 2022 |
| Tanzania | 2018, 2019, 2020 | Through demonstration and various research projects  Initiation: Not clear  Retention/adherence: Not clear | Quanti (IQ) | FSWs selected through a respondent driven sampling | n=700 (only HIV-) at baseline, n=562 at 12 months | [Oral/use: ever initiated the use of PrEP during the 12m follow-up period]  8%  (+quan) having already been married or cohabiting, sex with an HIV+ partner | Faini, 2022 |
| Tanzania | 2021 | Not clear.  Initiation: Not clear  Retention/Adherence: Appointment for PrEP refill 28 days following enrolment.  Use of an mHealth application on a smartphone, including information abvout PrEP, daily reminders to take PrEP, an online forum for anonymous discussions between PrEP users and the opportunity to consult a doctor and/or a peer educator online. | Quanti (PD, ID) | FSWs including in a quasi-experimental trial evaluating the effectiveness of a mHealth intervention for PrEP roll-out | n=470 who initiated PrEP | [Oral/retention: at 1 month after initiating PrEP]  28%  (+quan) use of an mHealth application, low sex work stigma, condom use during last sex with paying partner | Mbotwa, 2023 |
| Tanzania | 2021 | Through a sex worker-friendly satellite clinic.  Initiation: At the first visit  Retention/Adherence: Use of an mHealth app to promote adherence to medication and follow-up visits (alarms, possibility to contact staff) | Quanti (PD) | FSWs included in a cohort from an intervention site of a quasi-experimental trial evaluating the effectiveness of a mHealth intervention for PrEP roll-out | n=470 who initiated PrEP | [Oral/retention: having attended the first scheduled follow-up appointment in the PrEP programme (28 days after enrolment)]  25%  (+quan) higher number of clients, no mental distress | Lichtwarck, 2023 |
| Tanzania | 2021, 2022 | Not clear.  Initiation: Not clear  Retention/Adherence: Appointment for PrEP refill 28 days following enrolment.  In the control arm: reminders calls by peer educators to attend scheduled follow-up visits.  In the intervention arm: use of an mHealth application on a smartphone, including information about PrEP, daily reminders to take PrEP, an online forum for anonymous discussions between PrEP users and the opportunity to consult a doctor and/or a peer educator online. | Quanti (PD, ID) | FSWs including in a quasi-experimental trial evaluating the effectiveness of a mHealth intervention for PrEP roll-out | n=783 included in a clinical randomized trial  (n=470 in the intervention arm; n=313 in the control arm) | [Oral/retention: at 1 month after initiating PrEP]  25%  (+quan) use of an mHealth application (28% in the Intervention arm; 20% in the Control arm) | Mbotwa, 2024 |
| Tanzania | 2022 | Not clear  Initiation: Not clear  Retention/Adherence: Not clear | Quanti (IQ) | FSWs recruited through a respondent-driven sampling | n=428 (HIV-) | [Oral/use: ever used]  55% (of whom 97% currently)  (+quan) >2 children, positive attitude towards PrEP  [Oral/use: PrEP adherence]  (-quan cited) side effects (headaches, nausea, weight loss) | Martin, 2023 |
| Tanzania | 2022 | In clinics  Initiation: Not clear  Retention/Adherence: Not clear | Quanti (IQ) | FSWs recruited in health facilities | n=360 who initiated PrEP | [Oral/adherence: self-report + pill count]  - self report: 43%  - pill count: 48%  (+quan) living with family members, like pills packaging, having good perception of PrEP  - 58% declared challenges in accessing PrEP  (-quan cited) stigma, lack of privacy when accessing pills, distance from health facilities, work overload  - 65% with challenges in taking PrEP  (+quan cited) watch and phone reminder applications  (-quan cited) forgetfulness, side effects | Churu, 2023 |
| Tanzania | 2021, 2022, 2023 | In a health centre  Initiation: Not clear  Retention/Adherence: phone calls if missed visit | Quanti (IQ) | FSWs included in a cohort from a control group of a quasi-experimental trial evaluating the effectiveness of a mHealth intervention for PrEP roll-out | n=313 | [Oral/use: ever use]  At 6M and 12M: 99.5%  [Oral/adherence: >= 2 doses per week for anal sex and >=6 doses per week for vaginal sex]  Anal sex: 8% M6, 10% M12  Vaginal sex: 10% M6, 4% M12  (+quanti) living with friends (versus alone), self-reporting good health status, refusing condomless sex with a steady partner | Mikomangwa, 2024 |
| Uganda | 2018 | Through an urban clinic offering PrEP to FSWs and other key populations.  Initiation: Not clear  Retention/Adherence: phone calls if missed visit | Quanti (IQ) | FSWs visiting a clinic | n=126 who initiated PrEP | [Oral/adherence: taking PrEP 6 to 7 days/week]  71%  (-quan) not using condom on last sex act with clients, long term contraception methods (versus pills)  (-quan cited) side-effects ++, pill burden, stigma, size of pills, forget to take pills, PrEP stopping if unexpected trip | Nalukwago, 2021 |
| Uganda | 2018, 2019 | Through the Rakai Health Sciences Program including two central facilities and eight outreach sites  Initiation: Not clear  Retention/Adherence: phone calls if missed visit | Quanti (PD) | FSWs visiting one of the central facilities or outreach sites | n=1419 (HIV-) | [Oral/use: PrEP acceptance]  94% | Kagaayi, 2020 |
| Uganda | 2019, 2020 | Not clear  Initiation: Not clear  Retention/Adherence: Not clear | Quanti (IQ) | FSWs included in a trial evaluating adding savings,  financial literacy, and mentorship to traditional HIV risk reduction | n=350 (HIV-) | [Oral/use: already received a PrEP prescription]  16.6%  (+quan) having lower economic abuse | Mayo-Wilson, 2023 |
| Uganda | 2019, 2020 | Not clear  Initiation: Not clear  Retention/Adherence: Not clear | Quanti (IQ) | FSWs enrolled in a trial evaluating a combination intervention  adding microfinance components to traditional HIV  risk reduction | n=322 (HIV-) of whom n=286 not already on PrEP | [Oral/use: ever taking PrEP, agree to initiate PrEP]  11% ever on PrEP, 55% of those not on PrEP agree to initiate  (+quan) high family stigma related to sex work, low social support from friends  (+quan cited) inability to adhere to daily medication, fear of side-effects, fear of stigma | Witte, 2022 |
| Uganda | 2021 | Through the national PrEP program including clinics and outreach facilities.  Initiation: Outreach sensitization with peer leaders.  Retention/Adherence: Refill schedules flexible and usually made according to client preference. Phone calls when missed visits. | Quanti (IQ) | FSWs attending their PrEP routine follow-up in a health centres (facility-based or community-based) | n=524 who initiated PrEP | [Oral/adherence: pill count >85% or reporting taking PrEP everyday in the last 3m]  71% pill count, 50% self-report  (+quan) >35 years old, divorced/separated/widowed vs single/never married, having had a STI in the last 3m, having had sex with >100 person in the last 3m, preferring to take PrEP everyday | Nakiganda, 2022 |
| Uganda | 2023 | Not clear.  Initiation: Not clear  Retention/Adherence: Not clear | Quanti (IQ) | FSWs recruited in sex work venues and through a respondent driven sampling | n=273 (HIV-) | [Oral/use: in the past 3 months]  66%  (+quan) having regular source of income besides sex work, working in rural area, having depression symptoms, having already used postexposure prophylaxis | Bongomin, 2023 |
| Zambia | 2019;  2020;  2021;  2022;  2023 | 2019-2021: Through government health facilities, with key population-designated static safe spaces.  Since end-2021: PrEP offer on site with a decentralized approach with more convenient access.  Initiation: Involvement of peer navigators to conduct FSW community mobilization activities  Retention/Adherence: PrEP support groups managed by peer navigators | Quanti (PD) | FSWs recruited in sex work venues | n=7087 (HIV-) | [Oral/use: PrEP initiation]  98% | Sikazwe, 2024 |
| Zimbabwe | 2014, 2015, 2016 | Through the SAPPH-IRe trial nested in the HIV Sisters programme for FSW  Initiation: Not clear  Retention/adherence: SMS appointment reminders, phone calls if missed appointment, peer medication support, intensive community mobilization | Quanti (PD) | FSWs included in an HIV prevention trial | n=1302 (HIV- who were offered PrEP)  n=500 who initiated PrEP | [Oral/use: started PrEP]  38%  [Oral/retention: returned for at least one follow-up visit]  81% | Cowan, 2018 |
| Zimbabwe | 2014, 2015, 2016 | Through the SAPPH-IRe trial nested in the HIV Sisters  Initiation: Not clear  Retention/adherence: SMS appointment reminders, phone calls if missed appointment, peer medication support, intensive community mobilization | Quanti (IQ) | FSWs selected through a respondent driven sampling | N=611 (HIV-) | [Oral/use: ever been offered PrEP]  28.8%  [Oral/use-adherence: current taking PrEP every day]  15.6%  (+quan) older, longer duration of sex work (+ for women with steady partners: being adherent to condoms with those partners)  (-quan) having alcohol 2-3 times per week | Fearon, 2019 |
| Zimbabwe | 2017, 2018, 2019 | Through urban clinics.  Initiation: Not clear  Retention/Adherence: Not clear | Quanti (PD) | FSWs visiting PrEP services in clinics | n=145 who initiated PrEP | [Oral/retention: On PrEP 6m after initiation]  85% | Bero, 2023 |
| Zimbabwe | 2019, 2020, 2021 | In primary care clinics  Initiation: Not clearrmation  Retention/Adherence: In the intervention group: peer-led microplanning and self-help groups | Quanti (IQ) | FSWs included in a trial nested in the national programme for FSWs | Control group (usual care): n=1096 HIV-  Intervention group (outreach sites or drop-in centres): n=1200 HIV-  N=569 reporting taking PrEP | [Oral/use]  - Ever used  In the control group: 24.3%  In the intervention group: 32.4%  - Current used  In the control group: 15.7%  In the intervention group: 22.4%  [Oral/adherence: tenofovir diphosphate concentrations]  2% ≥700 fmol/dried blood spot punch  5.9% 350-700 fmol/punch | Cowan, 2024 |
| Zimbabwe | 2020 | Through the national Sisters porgamme, with services delivered to FSW in fixed-clinics, drop-in centres or mobile-clinics.  Initiation: Involvement of peer educators to encourage PrEP use.  Retention/Adherence: Phone calls with a clinician at week 1 and after the 1-month follow-up clinic visit, for side-effetcs and adherence counselling. Possibility for FSW to report any adverse events via phone and WhatsApp. Virtual support through Whatsapp with peer educators (to address PrEP myths, encourage uptake and adherence, and check concerns). | Quanti (PD) | FSWs receiving services from a comprehensive HIV prevention and treatment programme for sex workers, at facility or within the community | n=9,407 in total (HIV-): 1500-3000 each month | [Oral/use: PrEP initiation]  33.7% in total; peak at 51% (when Covid-19 epidemic with less movement from FSW)  (+quan) higher in 20-24 years old (while lower in >40 years old) | Matambanadzo, 2021 |
| Kenya | 2017, 2018, 2019 | Integration of PrEP into routine health services in drop-in centres, public and private health facilities.  Initiation: Community mobilization by peer educators, who referred FSW interested in PrEP to the facilities. HIV test, eligibility screening and clinical assessment occurred the same day.  Retention/Adherence: Planned one month after PrEP initiation. Adherence support with SMS reminders, phone calls reminders, PrEP support groups and adherence buddies. | Mix (PD, FGD) | FSWs included in a large-scale routine service PrEP delivery project | PD: n=211927 (HIV-)  FGD: n=44  N=17794 who initiated PrEP | [Oral/use: PrEP initiation]  (sreening: 42% of the 211927 HIV-)  28% of the 63724 eligible  (+quan) higher in younger FSW  (-qual) myths and misconceptions about PrEP, co-location of both PrEP and HIV services  [Oral/retention]  31% at M1, 8% at M3  (+quan) higher in older FSW  [Oral/adherence]  (-qual) side-effects (tiredness), stigma related to misconceptions about PrEP (associated with being HIV+), time for clinic visits | Were, 2020 |
| Kenya | 2023 | Key Populations clinics in Nairobi  Initiation: first visit after HIV testing  Adherence: via rapid urine-based test for tenofovir | Mix (IQ, FGD) | FSWs recruited in sex work venues | IQ: n=658  FGD: not clear | [Oral/use]  4.4% (n= 57 tested)  (-quali) side effects, stigma, low perceived risk, daily pill taking | Bhattacharjee, 2024 |
| South Africa | 2017, 2018 | PrEP offered in sex worker clinics and provided for a minimum of three months  Initiation: Not clear  Retention/Adherence: Not clear | Mix (IQ; IDI) | FSWs visiting NDoH clinic visits | IQ: n=175 (previously heard of PrEP)  IDI: n=20 | [Oral/use]  38% current user; 28% previous user; 34% never user  (+quan cited) feeling being at risk for HIV, having multiple partners  (-quan cited) not being offered PrEP, fear of side effect, fear of stigma, concerns with taking daily pills  (+qual) want to remain HIV-, partner refuse condom, encouragement from others, receiving comprehensive information (posters, fact sheet, FAQs…)  [Oral/adherence]  (-quan cited) side effects affecting daily life (stomach pain, vomiting, nausea), feeling stigmatized | Pillay, 2020 |
| Zimbabwe | 2014, 2015, 2016 | Through clinics in the SAPPH-IRe trial nested in the HIV Sisters programme for FSW  Initiation: Not clear  Retention/adherence: SMS appointment reminders, phone calls if missed appointment, peer medication support, intensive community mobilization | Mix (PD, IDI) | FSWs included in the trial | PD: n=1302  IDI: n=36 | [Oral/use: registered to take PrEP on those eligible]  38%  (-qual) too numerous eligibility screening appointments from PrEP registration to PrEP initiation, fear of side-effects | Busza, 2019 |
| Zimbabwe | 2014,  2015,  2016 | Through clinics of the the SAPPH-IRe trial nested in the HIV Sisters programme for FSW  Initiation: Not clear  Retention/adherence: SMS appointment reminders, phone calls if missed appointment, peer medication support, intensive community mobilization | Mix (PD, IDI) | FSWs included in the trial | PD: n=1403  IDI (2014 only): n=19 (6 never on PrEP, 3 new on PrEP, 2 who initiated PrEP but defaulter, 8 on PrEP with good adherence) | [Oral/use: initiated PrEP after expressing PrEP interest and initial screening; no delay mentioned]  45.5%  (+qual) discussion with family members or peers  (-qual) fear of side effects, concerns about taking a daily pill, bad experience with medication  [Oral/retention: returned for at least one follow-up appointment; no delay mentioned]  81.5%  (-qual) travelling  [Oral/adherence]  (+qual) strategies to remember pills (i.e phone alarm, keeping pills in the same bag as the phone, taking PrEP alongside oral contraceptives, linking drug taking to a daily event), peer adherence support  (-qual) side-effects (dizziness, headaches, stomach pain, nausea, sleepiness, general malaise/weakness) | Busza, 2021 |
| Zimbabwe | 2017, 2018, 2019 | Through the DREAMS demonstration project.  Initiation: Not clear  Retention/adherence: Not clear | Mix (IDI, IQ) | FSWs selected through a respondent driven sampling | IQ: n=963 in 2017, n=538 in 2019 (only HIV-)  IDI: n=43  Note: include 66% of women who considered themselves as FSW | [Oral/use: ever taking PrEP]  7% in 2017, 34% in 2019  (+quan) high number of clients, longer time in sex work, having visited a FSW program, agreed that friends/peers would support PrEP use  (-qual) fear that PrEP would disclose sex work, HIV-related stigma, costs of going to the clinic to access PrEP, concerns regarding adherence  [Oral/adherence: currently use PrEP]  11.5%  (-qual) not receiving support from family, side-effects, experience of stigma | Hensen, 2021 |
| Ethiopia | 2021 | Through drop-in centres offering confidential, comprehensive, anonymous services for FSW since 2020  Initiation: Not clear  Retention/adherence: Not clear | Quali (IDI) | FSWs selected using a snowball sampling | n=17 (10 who started PrEP, 1 who discontinued, 6 who did not start PrEP) | [Oral/use]  (+qual) minimize the risk of contracting HIV in case of condom failure  (-qual) PrEP misconceptions, poor confidence/self-doubt, burden of daily pill, fear of low PrEP adherence especially in case of alcohol consumption, fear of clients ask them to have sex without condom if they know they take PrEP, fear of side-effects  [Oral/adherence]  (-qual) adverse side-effects (nausea, gastritis, bloating, nightmares, back pain, diarrhoea), stigma as confusion between PrEP and ART in the community, alcohol use, forget if taken in the morning | Amogne, 2022 |
| South Africa | Missing | Through mobile clinics operated by various NGO.  Initiation: Not clear  Retention/Adherence: Not clear | Quali (IDI) | FSWs recruited in sex work venues | n=24 interested in PrEP use | [Oral/PrEP use]  (+quali) PrEP awareness, perceived benefits for HIV prevention, availability of mobile clinics  [Oral/PrEP retention]  (-quali) shortage of PrEP or unavailability in clinics, side effects (vomiting, menstrual changes, nausea, swelling, loss of appetite), lack of awareness about collection points or clinics being too far, concerns regarding relying solely on pills, fear of stigmatization | Mapetla, 2023 |
| South Africa | 2015, 2016, 2017 | Through the TAPS project in clinics  Initiation: possible 1 week after biological assessment  Retention/adherence: short SMS reminders for clinic visits + positive messages around adherence | Quali (IDI) | FSWs visiting HIV clinics | n=18 1^st^ round (M3)  n=10 2^nd^ round (M6)  n=6 3^rd^ round (M9) | [Oral/use]  (+qual) having safer sex and protect their own health especially if condoms breakage or in case of violence or with their main partners (with who condoms are not usually employed), the fact to see other person taking PrEP  (-qual) fear to be tested for HIV, disbeliefs and lack of knowledge regarding PrEP efficacy (after decades of messaging regarding condoms), fear to be identified as HIV+  [Oral/retention]  (+qual) counselling from clinician to cycle on and off PrEP when they went back home or stopped sex work for a time  [Oral/adherence]  (+qual) reminder strategies (such as phone alarms, stashing pills in case of late nights or placing the pill bottle in a routine location like next to a tooth brush), PrEP sharing, supports from family and friends | Eakle, 2019 |
| South Africa | 2018 | Not clear.  Initiation: Not clear  Retention/Adherence: Not clear | Quali (IDI, FGD) | FSWs selected using a snowball sampling | IDI: n=17  FGD: n=9 in 2 groups | [Oral/use]  (+qual) empowering tool for HIV prevention, source of freedom from HIV scars, especially when partner do not use condom | Makhakhe, 2022 |
| South Africa | 2018 | Not clear.  Initiation: Not clear  Retention/Adherence: Not clear | Quali (IDI, FGDI) | FSWs selected using a snowball sampling | IDI: n=17  FGD: n=9 in 2 groups | [Oral/use]  (-qual) low perception of HIV risks, PrEP misunderstanding and misconceptions in the community (especially related to confusion with ARVs) -> fear of stigmatization including conflicts with peers or broken relationships; fear that PrEP jeopardize hidden sex work identity | Makhakhe, 2022 |
| South Africa | 2020 | Through a drop-in centre  Initiation: Not clear  Retention/Adherence: Not clear | Quali (IDI) | FSWs recruited in a trial evaluating adaptive HIV treatment strategies | n=36 HIV- (on PrEP or not) | [Oral/use-adherence]  (+qual) high HIV risk perception, desire to live a long life, concerns regarding condoms breaking or condomless sex  (-qual) experiencing side effects (nausea, loss of appetite, headaches), limited understanding of how to use PrEP correctly, stigma tied to sex work and to PrEP use (especially wehn visiting family), high mobility and temporary relocation (for example eo go back to their hometown), service delivery and PrEP accessibility (when mobility), communication issues (phone number change), criminalization of sex work, COVID-19 policies (lockdowns, travel restrictions) | Shipp, 2024 |
| Tanzania | 2021, 2022 | Through a NGO satellic clinic  Initiation: Not clear  Retention/Adherence: Not clear | Quali (IDI) | FSWs recruited through a  peer chain referral method and eligible to PrEP | n=40 | [Oral/use-adherence]  (+quali) feeling of freedom from HIV fear and positive effect on wellbeing and autonomy, PrEP as empowering and a way to prioritize health and test for HIV more often  (-qual) side effects (nausea, dizziness, hunger, feeling of a racing heart) | Lichtwarck, 2024 |
| Uganda | 2016/17,  2019 | Through the RHSP PrEP demonstration program  Initiation: Not clear  Retention/adherence: including follow-up phone reminders and adherence counselling | Quali (IDI, FGD) | FSWs recruited from an existing list of community members who had agreed to be contacted to participate in studies as well as using a snowball sampling | IDI (2016/17): n=12  IDI (2019): n=17 (3 declined PrEP, 2 newly started PrEP, 6 on PrEP and adherent, 2 on PrEP but non adherent, 4 discontinued PrEP; among others)  FGD (2019): n=62 women (including other community members) | [Oral/use]  (+qual) effective method especially when partners giving financial incentives for condomless sex, forcing condomless sex or unplanned sex work without having condoms, supportive peer environment, counselling from experienced PrEP users  (-qual) fear of stigma/violence if PrEP is mistaken for ART  [Oral/adherence]  (+qual) counselling from experienced PrEP users, phone reminders  (-qual) mobility, side-effects that can affect daily functions as well as energy and work performance, drug sharing, alcohol use (forget daily pill or miss picking refills)  [Oral/retention]  (+qual) peers can pick up refills for others  (-qual) occupation limiting time off work (no time to go for appointments or to pick up refills) | Ddaaki, 2021 |
| Uganda | 2017, 2018, 2019 | Through the PR-e-Exposure Prophylaxis Priming of Immune Effectors  Initiation: Not clear  Retention/Adherence: Not clear | Quali (IDI) | FSWs enrolled in a longitudinal cohort of women at high risk of HIV and STIs, recruited in sex work venues | n=30 at different time points (only HIV-) | [Oral/adherence]  (-qual) fear of stigmatization while being seen taking pills (as HIV+), alcohol use that inhibit the ability to take the pills at a consistent time, irregular working hours, side effects | Kawuma, 2021 |
| Uganda | 2018, 2019, 2020 | Through the Empower study, a randomized trial evaluating HIV self-testing and PrEP  Initiation: Not clear  Retention/Adherence: Monthly phone interviews and quarterly clinic visits | Quali (IDI) | FSWs recruited in a trial evaluating HIV self-testing | n=21 | [Oral/use]  (+qual) safer if no condom (economic empowerment) -> reduce anxiety and permit pleasure (relational empowerment), reduce the need for clinic visits | Mujugira, 2021 |
| Uganda | 2019, 2020 | Not clear.  Initiation: Not clear  Retention/Adherence: Not clear | Quali (IDI) | FSWs recruited in a trial evaluating an economic empowerment component | n=20 | [Oral/use]  (+qual) allow to feel safe in case of the customer wants unprotected sex | Sensoy Bahar, 2022 |
| Zambia | 2021 | Not clear.  Initiation: Not clear  Retention/Adherence: Not clear | Quali (FGD) | FSWs identified by health centres staff | n=43 FSW who initiated PrEP | [Oral/adherence]  (-quali) difficulty of remembering a daily pill (especially when highly mobile), experience of side effect (weight changes, feeling ‘burnt’, dizziness, headaches, nausea, weakness), fear of stigmatization as PrEP looks like ARVs | Evens, 2024 |
| Zambia | 2021 | Through a clinic with staff trained in key population sensitivity, safety and security  Initiation: Community mobilization with CHWs  Retention/Adherence: PrEP support groups managed by peer navigator | Quali (IDI, FGD) | FSWs recruited in the clinics PrEP was offered | IDI: n=12  FGD: n=13 in 2 groups | [Oral/use-adherence]  (+qual) perceived HIV risk, difficulty with turning away clients willing to pay more for sex without condom, sexual violence including coerced sex with police officers, willing to remain healthy and HIV-free for their children, social support (family, friends, partners)  (-qual) experience of side-effects (vomiting, diarrhoea, headaches, dizziness, nightmares, fatigue), confusion between PrEP and ART and fear of being identified as a HIV-positive person with consequences (partner violence, being thrown out of living situation, relationships ending, losing clients), anticipated stigma, excessive alcohol use, mobility, size of pills, having to take a pill every day at the same time, long queues and waiting times, clinic hours conflicting with work schedules, drug stock-out especially during COVID-19 | Stoebenau, 2024 |
| Zimbabwe | 2020 | In a specialized clinic for FSWs  Initiation: Not clear  Retention/Adherence: Not clear | Quali (IDI) | FSWs selected in a specialized clinic where they initiated PrEP | n=20 who initiated PrEP | [Oral/use]  (+qual) self-protection from HIV especially if unprotected sex, condom bursting, way to earn more money in case of unprotected sex, encouragement from others, need to take care in children, prior participation in HIV prevention research studies, gender based violence | Nhamo, 2022 |
| Malawi;  South Africa;  Uganda;  Zimbabwe | 2020,  2021,  2022 | Both oral and injectable as part as randomized trial  Initiation: Not clear  Retention/Adherence: Not clear | Quali (IDI) | FSWs recruited in a trial evaluating oral PrEP versus injectable Cabotegravir | n=19 | [Injectable CAB/use]  (+qual) more convenient than daily oral regimen (not require daily remembering, fit better into women’s lifestyles especially when travelling or having unpredictable work), more effective protection as longer duration, privacy from sexual clients or friends/family  (-qual) painful but tolerated  [Oral/use-adherence]  (-qual) concerns about PrEP adherence, especially if late night work, heavy drinking and unexpected travelling, side effects (hunger pains, dizziness, nausea, headache, diarrhea, sleepiness, or sweating) | Tolley, 2024 |
| **Western and Central Africa** | | | | | | | |
| Benin | 2014, 2015, 2016 | Through a demonstration project in Cotonou  Initiation: Not clear  Retention/Adherence: Community workers regularly reminded study participants of their visits by phone or by visiting them at home or their work place. | Quanti (IQ, PD) | FSWs included in a PrEP demonstration study | n=256 at baseline, n=225 at D14, n=189 at M6, n=150 at M12 | [Oral/retention: PrEP follow-up visits]  88% at D14, 80% at M3, 74% at M6, 59% at M12, 51% at M24  [Oral/adherence: self-report of 7 pills in the last week]  <60% | Mboup, 2018 |
| Benin | 2014, 2015, 2016 | Through a demonstration project.  Initiation: Not clear  Retention/Adherence: Monthly visits, with possible supply for 3 months if needed. | Quanti (PD) | FSWs included in a PrEP demonstration study | n=255 who initiated PrEP | [Oral/retention: PrEP follow-up visits]  88% at D14, 80% at M3, 74% at M6, 59% at M12, 30% at M18, 12% at M24  [Oral/optimal adherence: tenofovir concentration, self-report, pill count; 7 pills/week]  TDF concentration: 26% (50% at D14 and 15% at M24)  Self-report: 56% (78% at D14, 46% at M18, and 60% at M24)  Pill count: 19% (46% at D14, 10% at M18, and 31% at M24) | Mboup, 2020 |
| Benin | 2014, 2015, 2016 | Through a PrEP demonstration study among professional FSWs  Initiation: Not clear  Retention/Adherence: Monthly visits | Quanti (IQ, PD) | FSWs included in a PrEP demonstration study | n=255 who initiated PrEP | [Oral/retention: end of the study]  M12: 55.5%  M18: 50.8%  (-quan) mobility  [Oral/adherence: all pills taken in the last 7 days]  34,1% - decrease over  (+quan) older age, high intention to adhere  (-quan) time of follow-up (long time = decrease follow-up) | Mboup, 2021 |
| Cameroon | 2019, 2020 | Since 2019 countrywide.  CHAMP programme, through drop-in centres.  Initiation: Peer mobilization in community during routine activities and online outreach. Several steps for PrEP screening  Retention/Adherence: Monthly visits in the first 3 months, then 3-month PrEP dispensation for ahdrent clients. Call phone 7-10 days  after initiation to enquire about side effects, adherence, and offer support. | Quanti (PD) | FSWs included in a programme offering PrEP services | n=9081 mobilized (having ask for more PrEP information)  n=1128 eligible to start PrEP | [Oral/use: PrEP assessment then PrEP initiation]  PrEP assessment: 15% among those mobilized  PrEP initiation: 61% among those eligible  [Oral/retention: PrEP continuation = PrEP drug refill]  26% at M3, 19% at M6, 5% at M12  (-quan) multiple missing appointments, drug stuckout or expired, nonaherence, severe undesired side-effects, mobility, no longer at substantial risk | Ndenkeh, 2022 |
| Democratic Republic of Congo | 2018 | Through a demonstration project in key-populations-friendly clinics  Initiation: immediate  Retention/Adherence: counselling at initiation then each visit | Quanti (PD) | FSWs included in a PrEP demonstration study | Not clear how many FSWs were offered daily PrEP  n=277 HIV- who initiated PrEP | [Oral/use: PrEP initiation]  (-quan cited) no interested in daily medication, no believe they need PrEP  [Oral/retention: PrEP follow-up visits]  62% at M1, 81% at M3, 87% at M6 | Franks, 2022 |
| Ghana | 2020 | Very beginning (since 2020)  Initiation: Not clear  Retention/Adherence: Not clear | Quanti (IQ) | FSW recruited in sex work venues | n=998 (HIV- who already heard of PrEP) | [Oral/use: already take PrEP]  6%  (+quan) older age, already screened for STI | Guure, 2022 |
| Kenya | 2019 | Through a sex worker outreach program  Initiation: Not clear  Retention/Adherence: Not clear | Quanti (IQ) | FSW visiting a clinic and included in a longitudinal study | n=746 HIV- (baseline questionnaire) | [Oral/use: current PrEP use]  24%  (+quan) having completed secondary education, feeling empowered to use PrEP, having low wealth tertile, high alcohol and/or substance use score, having a current non-paying intimate partner, not having experience violence from an intimate partner in the past 6m, having support from sex worker organizations, experiencing sex work stigma, seeking health services in spite of sex work stigma, experiencing violence from authorities in the past 6m  [Oral/adherence: self-reported (nb of missing doses in the last 7 days) and using LFA urine test]  Self-report over the last 7 days: 85%  LFA measure (adherence in the last 4-7 days): 31%  (+quan / LFA) : having completed primary education or less, having a high wealth tertile, having experienced emotional violence in childhood, being nulliparous. | Shah, 2024 |
| Nigeria | 2020 | Since 2015 countrywide  Initiation: Not clear  Retention/Adherence: Not clear | Quanti (IQ) | Not clear | n=824 | [Oral/use: currently use]  19%  (+quan) low cost of PrEP  (-quan) difficult to take it daily, need to still use condom while on PrEP | Durosinmi-Etti, 2022 |
| Nigeria | 2020 | Not clear | Quanti (IQ) | FSWs visiting a community-based clinic | n=344 (only HIV-) | [Oral/use: already]  42%  (+quan cited) do not want to use condom, clients paid more not to use condom | Omo-Emmanuel, 2023 |
| Nigeria | 2020 | Initiation: Not clear  Retention/Adherence: Not clear | Quanti (IQ, PD) | FSWs recruited in sex work venues | n=288 (only those who already know PrEP) | [Oral/use: PrEP uptake]  14%  (+quanti) formerly married, single  (-quanti) others or none religion than christianity, unemployed, out of the labor force | Olakunde, 2024 |
| Senegal | 2015, 2016 | Through the PrEP Senegal Demonstrations project in four government-run clinics in Dakar  Initiation: At the enrollment visit  Retention/Adherence: Visits were scheduled at seven days, at 1 month and every 3 months until 12 months after enrolment. Adherence counselling was routinely provided at each visit. | Quanti (PD) | FSWs included in a demonstration project | n=324 eligible to PrEP  n=267 who initiated PrEP | [Oral/use: PrEP initiation]  82%  [Oral/retention]  90% at M1, 80% at M6 and 73% at M12  (+quan) older age  [Oral/adherence: self-reported ; with TFV/FCT concentration]  Self-reported: About 50-60%  (-quan cited) forgot, too busy with other things, ran out of study pills  FTV/FCT concentration: 51.8% | Sarr, 2020 |
| **Middle East and North Africa** | | | | | | | |
| Morocco | 2017,  2018 | Through ALCS (Association de Lutte Contre le Sida) sexual health clinics  Initiation: At the enrolment  Retention/Adherence: Every months during 5 months follow-up | Quanti (PD) | FSWs visiting health centres | n=97 HIV- | [Oral/use: PrEP initiation]  83%  [Oral/retention]  26% at M3  16% M5+ | Ben Moussa, 2024 |
| Morocco | 2021 | Through ALCS (Association de Lutte Contre le Sida) sexual health clinics  Initiation: Not clear  Retention/Adherence: Not clear | Quali (IDI) | FSWs visiting health centres | n=10 | [Oral/use-adherence]  (+qual) being protected in case of clients’ refusal to use condoms, inability to negotiate condom use with clients and condom failure  (-qual) fear of being labelled as HIV-positive and being stigmatized, preference for condoms they used to use and they also protect from unwanted pregnancy, not feeling sick, lack of PrEP awareness among healthcare workers and FSW community, financial burden (for transportation from and to the PrEP clinic), size of PrEP pills, experience of side effects (vomiting), concerns regarding daily use due to lifestyle (substance use, heavy drinking) | Moussa, 2024 |
| **Western and central Europe and North America** | | | | | | | |
| USA, Rhode Island | 2014, 2015, 2016, 2017 | Not clear | Quanti (PD) | FSWs recruited in a STI clinic | n=34 | [Oral/use: ever taken PrEP]  0% | Sosnowy, 2020 |
| USA, Oregon | 2016 | Not clear  Initiation: Not clear  Retention/Adherence: Not clear | Quanti (IQ) | FSWs recruited through respondent-driven sampling | n=334 | [Oral/use: ever taken PrEP]  <1% | Menza, 2020 |
| USA, Maryland | 2019, 2020 | Through a harm reduction drop-in center. PEARL trial.  Initiation: Same day as enrolment  Retention/Adherence: PrEP available at a pharmacy of participant’s choice. In intervention arm: i) peer navigators doing face-to-face and phone counseling,  appointment reminders, referrals, and  accompanying participants to medical and social service appointments, ii) mHealth including appointment and daily medication reminders, iii) 2 hours small group sessions to discuss PrEP. | Quali (IDI, FGD) | FSWs visiting a health centre and included in a trial | n=39 | [Oral/use]  (-qual) medical mistrust, concers regarding the class action lawsuit against Gilead  [Oral/retention]  (-qual) intermittent phone access and housing instability limiting the involvement of peer navigators  [Oral/adherence]  (-qual) side-effects | Pelaez, 2021 |
| USA, Massachusetts | 2021, 2022 | Through two bridge clinics located in a hospitals.  Initiation: Not clear  Retention/Adherence: Not clear | Quali (IDI) | FSWs visiting two bridge clinics | n=25 (only self-reported HIV-) | [Oral/use, adherence]  (+qual) to protect in case of inadequate condom access or condom failure during sex work  (-qual) concerns about medication side effects, mistrust in medical settings, fear of daily PrEP adherence when lack housing (reason for PrEP stop) | Harris, 2024 |
| **Eastern Europe and Central Asia** | | | | | | | |
| **Asia and Pacific** | | | | | | |  |
| India | 2016, 2017 | In both outreach and static community clinics.  Initiation: Involvement of peer outreach workers, who identified potential participants in the field, and accompanied them to the lab after counselling and physical examination. Results available the same day to minimise loss to follow-up.  Retention/Adherence: PrEP dispensation individualised | Quanti (PD) | FSWs included in a demonstration project | n=652 eligible to PrEP  n=647 who initiated PrEP | [Oral/use: enrolled to PrEP]  >99%  [Oral/retention: PrEP follow-up visits]  98% at each visit (M1, M3, M6, M9, M12, M15)  [Oral/adherence: self-reported daily adherence in the last month]  75% at M1, 69% at M3, 90% at M6 | Reza-Paul, 2020 |
| India | 2016, 2017, 2018 | Through a PrEP demonstration project  Initiation: one week after screening & biological assessment  Retention/Adherence: PrEP delivery and adherence support either at weekly clinic pickup or through home delivery by a peer educator | Quanti (PD, IQ) | FSWs included in a PrEP demonstration project | n=728 eligible to PrEP  n=678 who initiated PrEP | [Oral/use: PrEP initiation]  93%  [Oral/retention]  89% at M15  (-quan cited) main reasons for drop-out: no longer doing sex work, PrEP side effects, lack of interest  [Oral/adherence: self-report daily adherence + plasma tenofovir≥40 ng/ml]  Self-report: 73%  Plasma tenofovir: 40-65% | Jana, 2021 |
| **Latin America and the Caribbean** | | | | | | | |

*IDI: in-depth interview; FGD: focus-group discussion; IQ: individual questionnaire; CM: consultative meetings; PD: programmatic data; OBS: observations*

*(+qual) / (+quan): facilitators / factors positively associated with PrEP effective acceptability, in qualitative and quantitative studies, respectively*

*(-qual) / (-quan): barriers / factors negatively associated with PrEP effective acceptability, in qualitative and quantitative studies, respectively*

** if several types of data were collected, only the type(s) of data collection with specific results on PrEP a posteriori acceptability are indicated*
