# Supplementary material for: Implementing HIV pre-exposure prophylaxis (PrEP) among cisgender female sex workers: A worldwide scoping review to guide the future research agenda for HIV prevention
Source: PLOS Glob Public Health. 2026 Nov 13;6(11):e0007582. doi: 10.1371/journal.pgph.0007582 (PMC13766235; doi:10.1371/journal.pgph.0007582)
Supplement: Supplementary file 5 [file pgph.0007582.s006.docx]

**S6 Table. Other outcomes of PrEP use [Scoping review on PrEP among female sex workers - until end 2024]**

*Classified by UNAIDS region, type of data, country and year of data collection*

| **Country** | **Years of data collection** | **Type of data collection*** | **Place of recruitment** | **Population size: FSW only** | **Impact**  **[Outcome]** | **First author, Publication’s year** |
| --- | --- | --- | --- | --- | --- | --- |
| **Eastern and Southern Africa** | | | | | | |
| Kenya | 2017, 2018, 2019 | Quanti (PD) | FSWs visiting health facilities to receive PrEP | n=2621 | [Condom use: self-reported condom use]  Significant increase in self-reported condom use over time until M3 since PrEP initiation. (+quan) being >35 years old, having a live-in partner (married/cohabiting), receiving care in a drop-in center, not reported recent history of STI, not reported engaging sex under the influence of alcohol and recreational drugs | Manguro, 2022 |
| Kenya | 2018, 2019, 2020 | Quanti (ID) | FSWs visiting health facilities to receive PrEP | n=626 | [PrEP related-stigma at baseline: based on a scale with 12 items adapted from the Berger HIV stigma scale]  Higher score on disclosure concerns, then PrEP norms, then interpersonal stigma, then negative self-image.  PrEP-related stigma associated with low HIV risk perception. | Atkins, 2022 |
| Uganda | 2021 | Quanti (IQ) | FSWs attending their PrEP routine follow-up in a health centres | n=524 | [PrEP related to sexual wellbeing and HIV-related worry]  80.9%: sex more pleasurable because of PrEP  76.3%: less worried about acquiring HIV | Nakiganda, 2024 |
| **Western and Central Africa** | | | | | | |
| Benin | 2014, 2015, 2016 | Quanti (cohort : IQ) | FSWs included in a PrEP demonstration project | n=255 at baseline; 189 at M6; 151 at M12; 76 at M18; 30 at M24 | [Unprotected sex (vaginal sex act and inconsistent condom use or condom malfunction: i) self-report, ii) biological measurements]  No risk compensation while on PrEP  [STI]  Significant decrease of STI over time since PrEP initiation: 15.8% at baseline -> 9.0% at M12 -> 2.1% at M24 | Giguère, 2019 |
| Senegal | 2015, 2016 | Quanti (ID) | FSWs included in a PrEP demonstration project | n=267 | [Condom use: self-reported condom use + presence of Y-chromosomal DNA in vaginal swabs]  No increase in self-reported condomless sex or Yc-DNA detection over time until M12 since PrEP initiation. | Roberts, 2020 |
| **Middle East and North Africa** | | | | | | |
| **Western and central Europe and North America** | | | | | | |
| **Eastern Europe and Central Asia** | | | | | | |
| **Asia and Pacific** | | | | | | |
| India | 2016, 2017 | Quanti (PD) | FSWs included in a demonstration project | n=645 | [Condom use: self-reported from M1 to M15 on PrEP]   - during last sex with clients: >98% - during last sex with repeat clients: from 86% to 95% - during last week with regular partner: 62-63% | Reza-Paul, 2020 |
| India | 2016, 2017, 2018 | Quanti (PD) | FSWs included in a demonstration trial | N=728 | [Need of STIs treatment]  39% enrolment -> 13% at M15  [Consistent condom use]  At enrolment: 91% with occasional clients, 76% with repeat clients, 10% with intimate partners  Stable/increase over time since PrEP initiation | Jana, 2021 |
| **Latin America and the Caribbean** | | | | | | |

*IDI: in-depth interview; FGD: focus-group discussion; IQ: individual questionnaire; CM: consultative meetings; PD: programmatic data; OBS: observations*
