# Supplementary material for: Implementing HIV pre-exposure prophylaxis (PrEP) among cisgender female sex workers: A worldwide scoping review to guide the future research agenda for HIV prevention
Source: PLOS Glob Public Health. 2026 Nov 13;6(11):e0007582. doi: 10.1371/journal.pgph.0007582 (PMC13766235; doi:10.1371/journal.pgph.0007582)
Supplement: Supplementary file 6 [file pgph.0007582.s007.docx]

PGPH-D-26-01781
Implementing HIV pre-exposure prophylaxis (PrEP) among female sex workers: a worldwide scoping review to guide the future research agenda for HIV prevention
PLOS Global Public Health

Dear Editor and Reviewers,

We would like to thank you for all your comments, which have helped us improve the manuscript.

Please find below our responses to the Editor and Reviewers, along with the page numbers corresponding to the changes in the marked-up version of the manuscript.

Best regards,

Mélanie Plazy

**Journal Requirements:**

1. Please ensure all the standard sections present, (e.g. Abstract, Introduction, Results, Discussion, Methods), in the correct order, and that any references to subheadings in your main text are correct. An outline of the required sections can be consulted in our submission guidelines here: 
<https://journals.plos.org/globalpublichealth/s/submission-guidelines#loc-parts-of-a-submission>.

We thank the Editor for this comment. We confirm that we had followed the submission guidelines with the main standards sections in the following order: Abstract, Introduction, Methods, Results, Discussion.

In the submission guidelines, it is indeed indicated that: “The following elements can be renamed as needed and presented in any order”. Please do not hesitate to clarify this point if there is a need to modify any information.

2. We note that the most recent literature search was completed over a year before the manuscript was submitted to the journal. Systematic and scoping reviews should provide an up-to-date overview of the research topic covered. Therefore, we request that you please update your searches as part of your revision to ensure that the most up-to-date literature is included in your review, or provide scientific justification for not doing so. Please ensure you mention this in your response to reviewers, and discuss whether this has any impact on the Results and Conclusions of the study. If you are submitting a protocol, it is only necessary to state whether the searches will be updated prior to the publication of the results.

We thank the Editor for this comment regarding the currency of the literature search. We have carefully considered the possibility of updating the search as requested.

Unfortunately, this would involve substantially more work than a simple literature update. We have conducted a preliminary search on Pubmed to assess the extent of the additional literature, which yielded more than 150 records requiring careful screening, with approximately 55 potentially relevant studies requiring full-text assessment and detailed data extraction/content analysis. Given the extent of this additional work, we are concerned that undertaking an expedited update would not allow us to maintain the methodological rigor of the original review.

Nevertheless, we believe that the conceptual framework developed from the evidence available through December 2024 provides a valuable and relevant contribution to understanding PrEP implementation among female sex workers worldwide, and offers a useful basis for informing future research and PrEP programmes.

We have added a paragraph in the limitations to discuss the possibility that subsequent evidence may have extended or modified our findings (l.370-374).

*“Third, the resulting conceptual framework reflects a comprehensive synthesis of the available evidence on PrEP among cisgender FSWs worldwide through December 2024. However, subsequent research has been published since the search was conducted and may have further enriched or modified these findings, highlighting the need for continued monitoring of the evolving evidence on PrEP among sex workers.”*

3. We note that your Data Availability Statement is currently as follows: All relevant data are within the paper and its Supporting information files.
Please confirm at this time whether or not your submission contains all raw data required to replicate the results of your study. Authors must share the “minimal data set” for their submission. PLOS defines the minimal data set to consist of the data required to replicate all study findings reported in the article, as well as related metadata and methods
https://journals.plos.org/globalpublichealth/s/data-availability#loc-minimal-data-set-definition

 For example, authors should submit the following data:
 - The values behind the means, standard deviations and other measures reported;
 - The values used to build graphs;
 - The points extracted from images for analysis.
 
Authors do not need to submit their entire data set if only a portion of the data was used in the reported study.
If your submission does not contain these data, please either upload them as Supporting Information files or deposit them to a stable, public repository and provide us with the relevant URLs, DOIs, or accession numbers. For a list of recommended repositories, please see 
https://journals.plos.org/globalpublichealth/s/recommended-repositories

 If there are ethical or legal restrictions on sharing a de-identified data set, please explain them in detail (e.g., data contain potentially sensitive information, data are owned by a third-party organization, etc.) and who has imposed them (e.g., an ethics committee). Please also provide contact information for a data access committee, ethics committee, or other institutional body to which data requests may be sent. If data are owned by a third party, please indicate how others may request data access.

If the reviewer comments include a recommendation to cite specific previously published works, please review and evaluate these publications to determine whether they are relevant and should be cited. There is no requirement to cite these works unless the editor has indicated otherwise.

We confirm that the Supplementary files contain all the data needed to:

i) create the Figure 2 (description of the studies) as the tables are organized by themes (PrEP knowledge, PrEP a priori acceptability, PrEP acceptability) and for each of these tables, we present UNAIDS region as well methodology for all the included papers.

ii) reproduce the Figure 3 (conceptual framework) as all this has been developed using all the results presented in the tables in the Supplementary files

**Figure Resubmissions:**

While revising your submission, we strongly recommend that you use PLOS’s NAAS tool (<https://ngplosjournals.pagemajik.ai/artanalysis>) to test your figure files. NAAS can convert your figure files to the TIFF file type and meet basic requirements (such as print size, resolution), or provide you with a report on issues that do not meet our requirements and that NAAS cannot fix.

After uploading your figures to PLOS’s NAAS tool - <https://ngplosjournals.pagemajik.ai/artanalysis>, NAAS will process the files provided and display the results in the "Uploaded Files" section of the page as the processing is complete. If the uploaded figures meet our requirements (or NAAS is able to fix the files to meet our requirements), the figure will be marked as "fixed" above. If NAAS is unable to fix the files, a red "failed" label will appear above. When NAAS has confirmed that the figure files meet our requirements, please download the file via the download option, and include these NAAS processed figure files when submitting your revised manuscript.

We confirm that we have used the PLOS’s NAAS tool to test the figure files.

**Reviewers' comments:**

**Reviewer's Responses to Questions**

1. Does this manuscript meet PLOS Global Public Health’s publication criteria? Is the manuscript technically sound, and do the data support the conclusions? The manuscript must describe methodologically and ethically rigorous research with conclusions that are appropriately drawn based on the data presented.

Reviewer #1: Yes

Reviewer #2: Yes

Reviewer #3: Partly

2. Has the statistical analysis been performed appropriately and rigorously?

Reviewer #1: N/A

Reviewer #2: Yes

Reviewer #3: I don't know

3. Have the authors made all data underlying the findings in their manuscript fully available (please refer to the Data Availability Statement at the start of the manuscript PDF file)?

The PLOS Data policy requires authors to make all data underlying the findings described in their manuscript fully available without restriction, with rare exception. The data should be provided as part of the manuscript or its supporting information, or deposited to a public repository. For example, in addition to summary statistics, the data points behind means, medians and variance measures should be available. If there are restrictions on publicly sharing data—e.g. participant privacy or use of data from a third party—those must be specified.

Reviewer #1: Yes

Reviewer #2: Yes

Reviewer #3: Yes

4. Is the manuscript presented in an intelligible fashion and written in standard English?

PLOS Global Public Health does not copyedit accepted manuscripts, so the language in submitted articles must be clear, correct, and unambiguous. Any typographical or grammatical errors should be corrected at revision, so please note any specific errors here.

Reviewer #1: Ye

Reviewer #2: Yes

Reviewer #3: Yes

**5. Review Comments to the Author**

**Reviewer #1** (Pam Baatsen)**:** Overall, this is a well-written, interesting and timely scoping review that synthesises a large body of literature on PrEP among female sex workers. The topic is highly relevant and the review brings together valuable evidence on PrEP implementation in a population that remains relatively underrepresented in implementation research. The manuscript is generally well organised and will be a useful resource for researchers and programme implementers. I have several comments that I believe would further strengthen the manuscript.

We thank the reviewer for their interest in the topic and for their positive and encouraging comments on our work.

• The manuscript states that an adapted conceptual framework was used to organise the findings, but it is not entirely clear how this framework was adapted, or how it informed the synthesis of the included studies. It would be helpful to explain on what basis this was done.

We thank the reviewer for this comment.

We have developed our own conceptual framework based on two previous frameworks on the medication use and adherence.

-> the framework of Kvarnstrom et al was on Medication Adherence in Patients with a Chronic Condition. This framework identified barriers at six levels (patient, illness, medication, healthcare and system, social and culture, logistical and financial) as well as facilitators at five levels (adherence behaviour skills, information and knowledge, motivation, logistical and financial, healthcare and system).

-> the framework of Peh et al was on factors contributing medication adherence. It identified five main domains related to the medication, the condition, the patient, the socioeconomic status, and the healthcare system/healthcare professional.

For our framework, we kept the following five more relevant domains: patients’ characteristics, individual knowledge and perceptions, medication, social processes, structural and health system. We added a sixth domain related to sex work characteristics.

Once we had extracted all the results in the tables, we have synthetized and organized them according to the framework.

We have clarified this in the methods section (l.127-139) and data analysis (l.141-149)::

*“Data extraction.*

*Data extraction was organized in two steps. First, to provide a global cartography of available data on PrEP among FSWs, each included paper was independently reviewed by two research assistants (EM, CM, RM, SAP) to extract the following information using the PICO Portal software: Unaids region, country, study type, outcomes, type of PrEP, and study years. Any discrepancies were resolved with one senior researcher (MP, VB or JL). Secondly, a senior researcher (MP) with the help of a junior researcher (SAP) extracted specific results focusing on each of the following outcomes in using standardized Word tables (S3 Table, S4 Table, S5 Table): i) PrEP knowledge, ii) a priori PrEP acceptability (willingness or interest), iii) effective PrEP acceptability (use, retention, or adherence), iv) other outcomes of PrEP use. A coding system was used to classify findings as barriers or facilitators and according to whether they were derived from quantitative or qualitative data. Standardized data extraction tools (on PICO Portal software and on Word tables) were jointly developed by the three senior researchers (MP, VB, JL).*

*Data analysis.*

*A conceptual framework was first developed based on two conceptual frameworks on medication use and adherence [12,13]. Our conceptual framework includes six relevant domains derived from these frameworks, related to i) patients’ characteristics, ii) individual knowledge and perceptions, iii) medication, iv) social processes, v) structural and health system characteristics. We also added a sixth domain related to sex work characteristics. After data extraction, all drivers and barriers to a priori and effective acceptability of PrEP were analyzed and categorized within this conceptual framework.”*

• The exclusion criteria described in the Abstract appear to differ from those presented in the Methods section. A consistent description would be required.

We have modified the Abstract as follow (l.38-41):

*“Titles and abstracts, then full texts, were independently screened and papers were excluded if: not presenting specific or stratified results on cisgender FSWs; not focusing on PrEP; focusing on biological aspects of PrEP; not original research; modelling studies; not written in English or French; no full text available.”*

• The full title indicates that the review aims to "guide the future research agenda". Although research gaps are briefly mentioned in the Discussion, a more elaborate section identifying priority research questions and implementation gaps would better reflect the promise made in the title.

We thank the reviewer for this helpful comment. We have modified the Conclusion paragraph to better present the priorities for the future research agenda (l.392-412).

*In conclusion, this scoping review revealed that, although there is significant interest in PrEP, sustained engagement with oral PrEP is influenced by various constraints related to sex work, social processes and the healthcare system. This scoping review also highlights several research priorities. First, research should expand beyond the geographical concentration of existing evidence in Eastern and Southern Africa, particularly towards regions where evidence remains scarce, including the Middle East and North Africa and Eastern Europe and Central Asia. Second, as biomedical HIV prevention research continues to advance, efforts should focus on to addressing the specific challenges that FSWs face in preventing HIV, and identifying the most effective, suitable, and equitable HIV prevention strategies, including long-acting and other non-daily PrEP options. Research should determine whether these approaches can overcome barriers associated with daily pill-taking, mobility, unstable housing, unpredictable schedules and concerns about privacy, and identify which prevention options are preferred by different groups of FSWs. Finally, future research should include more longitudinal and implementation studies to improve sustained PrEP engagement under real-world conditions. Such studies should evaluate differentiated service delivery models and aligns the assess how PrEP delivery of prevention can be better aligned with the lived realities of sex work, mobility, stigma and constraints to service access. Together, these priorities can help translate the high interest in PrEP among FSWs into sustained HIV prevention impact.*

• In several instances, barriers are presented without explaining the underlying mechanisms. For example, unstable housing and alcohol use are listed as barriers to PrEP education (lines 165–166), while alcohol use is also mentioned as a barrier to adherence (lines 179–180). It would strengthen the review to briefly explain the mechanisms reported in the included studies.

We have added more explanations regarding the mechanisms in the results according to what was reported in the included studies:

*“However, one of the main barriers to a priori oral PrEP acceptability included concerns regarding adherence to a daily pill, especially considering that FSWs can have unpredictable lives, which can make it difficult to maintain a regular medication routine, or in the context of alcohol use, which can affect the ability to remember daily pill-taking [13,14,25,36,39,45,50–54,56,59,61,65,66].”* (l.193-196)

*[…]*

*“The burden of daily PrEP was cited by FSWs as a major barrier to oral PrEP initiation [29,32,40,82,84,104,121,122], retention and adherence [55,58,92,109,110,115,122,123,126], especially in the context of alcohol use, which can interfere with remembering to take the pills daily [58,71,81,118,122,126], or lack of housing, which can make it difficult to store medication safely [37].”* (l.238-241)

• Side effects are repeatedly identified as a barrier to PrEP adherence (e.g. lines 184 and 199–200), but the manuscript does not specify which side effects were most commonly reported. A brief summary would make these findings more informative for readers and programme implementers.

l. 184 and 199-200, side-effects referred to concerns about PrEP in relation to a priori acceptability (interest, willingness). The included studies did not provide sufficient detail on the specific side-effects that FSWs feared.

We have therefore added the side-effects experienced by FSWs and reported as barriers to PrEP continuation and adherence (l.246-251):

*“Fear of side effects could also prevent PrEP initiation [16,29,37,40,104,117,121,122,127], while experiencing side effects (e.g., headaches, gastrointestinal symptoms such as stomach pain, nausea, vomiting, bloating and diarrhoea, nightmares, back pain, menstrual changes, swelling, sweating, loss of appetite, weight loss and fatigue) was found as a barrier to PrEP continuation [29,32,48,52,55,58,71,82,95,99,104,109,110,115,117,118,121–123,126,128].”*

All these side-effects are also reported in the following Supplementary file: S5 Table. PrEP effective acceptability

• As expected for a scoping review, no formal quality assessment of the included studies was performed. While this is appropriate, the limitations of drawing conclusions from studies of varying methodological quality could be discussed more explicitly.

We agree with the reviewer. We have modified the sentence in the limitations part (l.375-378):

*“As a scoping review, it we did not formally assess the methodological quality or risk of bias of the included studies. This may affect the robustness and reliability of some findings and make it more difficult to assess the strength of the available evidence.”*

• As acknowledged by the authors, the majority of included studies originate from Eastern and Southern Africa. The Discussion could elaborate further on how this geographical concentration may limit the transferability of the findings to other settings where the organisation of sex work, health systems, and HIV prevention programmes differ.

We agree with the reviewer. We have expanded the limitation paragraph (l.381-388).

*“Lastly, publication bias remains a potential concern, as the results of some PrEP implementation projects, particularly those conducted in programmes or non-research settings, may have not been published. The geographical concentration of the evidence in Eastern and Southern Africa may also limit the transferability of our findings to other settings. The organisation and working conditions of sex work, healthcare systems, access to HIV prevention services, and the availability and implementation of PrEP may differ substantially across regions. Consequently, determinants of PrEP engagement and the feasibility or acceptability of specific service delivery models identified in the included studies may not be directly applicable to FSWs in other contexts.”*

• The Discussion briefly mentions differentiated service delivery models and long-acting PrEP. Given the rapidly evolving field, it would be helpful to briefly explain what is meant by differentiated service delivery and to discuss how newer PrEP modalities may help address some of the barriers identified in this review.

We have clarified what differentiated service delivery means (l.321-325):

*“In this context, improving accessibility and flexibility of services, including differentiated service delivery models that adapt where, when and how PrEP is delivered to the needs and preferences of FSWs, may help address these barriers. This could include outreach or easily accessible services, short waiting times and convenient opening hours [15,34,53,67,70,121,138].”*

We have also developed the paragraph on long-acting PrEP (l.334-341):

*“In this context, although research on long-acting PrEP remains limited among FSWs, the available studies suggest a strong interest in these methods, particularly injectable PrEP [140]. Such HIV prevention methods are perceived as reducing the daily pill burden, enhancing discretion, and potentially being better suited to unpredictable schedules and mobility. These potential advantages could be further enhanced when long-acting PrEP is delivered through differentiated service delivery models adapted to FSWs' needs, for example through outreach or community-based services that reduce the need for frequent clinic visits.”*

Minor comments

1. Several implementation terms and abbreviations would benefit from being defined when first introduced. For example CAB; the role of a peer navigator.

Thank you.
We have added the acronym the first time we use Cabotegravir (CAB) (l.82)

We have also added a definition for peer navigator (l.264): *“(i.e., trained peers with shared community experience who support FSWs in accessing PrEP)”*

2. The manuscript uses the term "prostitution sites" (line 225). Unless there is a specific reason to retain the original wording from the included studies, I would recommend using the more commonly accepted term "sex work sites".

We thank the reviewer. We have made the changes.

3. There are several minor grammatical and typographical errors throughout the manuscript (for example around line 131). A careful language edit would improve readability.

Thank you for this comment. We have reviewed all the manuscript using <https://languagetool.org>.

**Reviewer #2:** This manuscript portrays the importance of database analysis of PrEP, as a consideration for a better public policy regarding PrEP, which is very valuable for further studies. Even though the included studies mostly from Africa, the manuscript portrays the essential core points of what should be consider to make a better public policy regarding PrEP

We thank the reviewer for their interest in the topic.

**Reviewer #3:** Thank you for the opportunity to review this paper. The authors have done an admirable job of synthesizing a broad swatch of research.

We thank the reviewer for their interest our work, and for his positive assessment of our efforts to synthesize the available evidence on this important topic.

However, I have several concerns related to the scope and purpose of this scoping review as well as the methodological approach which limited my ability to assess the results and conclusions drawn by the authors.

1) Throughout abstract, introduction, methods, and discussion, the authors alternate between describing this as a review of PrEP interventions or of PrEP knowledge, interest, uptake, and use more generally around FSWs. This must be clarified, as these are two separate research questions. It appears as though this is about PrEP more broadly, since it does not look like articles had to be about “interventions.”

We thank the reviewer for their comment. Indeed, this scoping review is on all evidence of PrEP implementation among FSWs. We do not see where we wrote the term “intervention”?

2) Framing must specify that this is focused on cisgender women engaged in sex work only. This is mentioned briefly, but this should be clearly stated in the introduction and justified in the methods. Terminology should also be clarified (e.g., a statement along the lines of “in this manuscript, we use the terminology “female sex workers” to refer to cisgender women engaged in sex work.”)

We agree with the reviewer. We have added the term “cisgender” in the Title as well as in the objective of the abstract and the introduction part.

We have also explained this choice in the methods section (l.110-112):

*“We focused specifically on cisgender women to maintain a clear and sufficiently homogeneous scope, as the social, structural and healthcare contexts influencing PrEP engagement may differ from those of transgender women and other gender-diverse populations.”*

3) Introduction would benefit from better clarifying the purpose of this review in particular and how it adds to existing evidence. I recommend engaging with existing systematic reviews such as:

o Glick JL, Russo R, Jivapong B, Rosman L, Pelaez D, Footer KHA, Sherman SG. The PrEP Care Continuum Among Cisgender Women Who Sell Sex and/or Use Drugs Globally: A Systematic Review. AIDS Behav. 2020 May;24(5):1312-1333. doi: 10.1007/s10461-019-02733-z.

o Mpirirwe R, Segawa I, Ojiambo KO, Kamacooko O, Nangendo J, Semitala FC, Kyambadde P, Kalyango JN, Kiragga A, Karamagi C, Katahoire A, Kamya M, Mujugira A. HIV pre-exposure prophylaxis uptake, retention and adherence among female sex workers in sub-Saharan Africa: a systematic review. BMJ Open. 2024 Apr 25;14(4):e076545. doi: 10.1136/bmjopen-2023-076545.

The review from Mpirirwe has already been cited (ref 9 in the previous version; ref 10 in this updated version).

We have reviewed the introduction part to add the review from Glick et al (l.87-90):

*“Two previous literature reviews have been conducted on oral PrEP use among female sex workers: one included studies published up to the end of 2018 [9], while the other focused on Africa [10]. No recent comprehensive synthesis of PrEP research among cisgender FSWs worldwide is currently available.”*

4) The search strategy should be described separately from time frame (lines 96-99) for clarity. Describe search strategy (E.g., MESH terms) and then describe timeframe for published articles. In addition, I have some concerns regarding the search strategy (for example, why were articles required to have “PrEP” AND “HIV” in title or abstract? Were keywords also searched? How were the terms for sex workers chosen?). Was this strategy reviewed by a librarian?

We thank the reviewer for this comment. We have clarified the search strategy in the Methods, including the fact that fields were specific to the database. We have also separated the description of the search strategy from the search timeframe and specified that records published up to 31 December 2024 were included. The complete database-specific search equations are provided in S2 Fig.

We have clarified this point (l.98-105):

*“We have conducted a scoping literature review following the Preferred Reporting Items for Systematic Reviews and Meta-Analysis extension for Scoping Reviews (PRISMA-ScR) guidelines [11] (S1 Table). Six databases covering medical and social sciences literature were used: Pubmed, Scopus, SocINDEX, APA PsycArticles, APA PsycInfo, Psychology and Behavioral Sciences Collection. The search strategy combined the following terms, searched using database-specific Title and Abstract fields: (“PrEP” OR “preexposure prophylaxis” OR “pre-exposure prophylaxis”) AND (“HIV” OR “AIDS”) AND “sex-work*” (S2 Fig). The search included records published up to 31 December 2024.”*

No MeSH terms were used. The combination of PrEP and HIV-related terms was used to specifically identify studies addressing HIV pre-exposure prophylaxis. The term “sex work*” was selected as a commonly used term in the field to capture studies referring to sex work and sex workers, including in the title or abstract. The search strategy was developed by the research team and reviewed by the librarian of our research centre.

5) Why was “transactional sex” excluded from this review? This is a commonly used term, particularly in research conducted in sub-Saharan Africa. How was this defined and why was this form of sex work excluded?

We thank the reviewer for raising this point. In this review, we focused specifically on female sex workers, and therefore did not include studies focusing exclusively on transactional sex. We considered transactional sex as distinct from sex work, as the former may refer to sexual relationships involving an exchange of money, goods or other resources without necessarily constituting sex work as an occupation or livelihood.

6) How was data extraction standardized across qualitative and quantitative study types? How were data analyzed? What analyses were conducted?

7) Please better describe the conceptual framework, particularly how it was adapted to PrEP as a preventative medication rather than disease treatment.

Data extraction was conducted using standardized tables presented in the Supplementary files.

For numerical data describing knowledge, a priori acceptability (interest), and effective acceptability (use/uptake, retention and adherence), we specified the definitions in the text highlighted in grey.

For barriers and facilitators to PrEP a priori and effective acceptability, we used a coding system classifying findings as barriers (-) or facilitators (+) and according to whether they came from quantitative data (quan) or qualitative data (qual). The extracted data were synthesized descriptively and narratively across studies.

Regarding the conceptual framework, we have developed our own conceptual framework based on two previous frameworks on the medication use and adherence.

-> the framework of Kvarnstrom et al was on Medication Adherence in Patients with a Chronic Condition. This framework identified barriers at six levels (patient, illness, medication, healthcare and system, social and culture, logistical and financial) as well as facilitators at five levels (adherence behaviour skills, information and knowledge, motivation, logistical and financial, healthcare and system).

-> the framework of Peh et al was on factors contributing medication adherence. It identified five main domains related to the medication, the condition, the patient, the socioeconomic status, and the healthcare system/healthcare professional.

For our framework, we kept the following five more relevant domains: patients’ characteristics, individual knowledge and perceptions, medication, social processes, structural and health system. We added a sixth domain related to sex work characteristics.

We have clarified this in the methods section (l.127-149):

*“Data extraction.*

*Data extraction was organized in two steps. First, to provide a global cartography of available data on PrEP among FSWs, each included paper was independently reviewed by two research assistants (EM, CM, RM, SAP) to extract the following information using the PICO Portal software: Unaids region, country, study type, outcomes, type of PrEP, and study years. Any discrepancies were resolved with one senior researcher (MP, VB or JL). Secondly, a senior researcher (MP) with the help of a junior researcher (SAP) extracted specific results focusing on each of the following outcomes in using standardized Word tables (S3 Table, S4 Table, S5 Table): i) PrEP knowledge, ii) a priori PrEP acceptability (willingness or interest), iii) effective PrEP acceptability (use, retention, or adherence), iv) other outcomes of PrEP use. A coding system was used to classify findings as barriers or facilitators and according to whether they were derived from quantitative or qualitative data. Standardized data extraction tools (on PICO Portal software and on Word tables) were jointly developed by the three senior researchers (MP, VB, JL).*

*Data analysis.*

*A conceptual framework was first developed based on two conceptual frameworks on medication use and adherence [12,13]. Our conceptual framework includes six relevant domains derived from these frameworks, related to i) patients’ characteristics, ii) individual knowledge and perceptions, iii) medication, iv) social processes, v) structural and health system characteristics. We also added a sixth domain related to sex work characteristics. After data extraction, all drivers and barriers to a priori and effective acceptability of PrEP were analyzed and categorized within this conceptual framework.”*

8) A definition of a priori and effective PrEP acceptability is needed in the methods section.

A priori acceptability refers to willingness / interest.

Effective acceptability refers to uptake/use, retention and adherence.

These definitions are already defined:

- in Table 1.

- in Data extraction part (l.132-136): “*Secondly, a senior researcher (MP) with the help of a junior researcher (SAP) extracted specific results focusing on each of the following outcomes in using standardized Word tables (S3 Table, S4 Table, S5 Table): i) PrEP knowledge****, ii) a priori PrEP acceptability (willingness or interest), iii) effective PrEP acceptability (use/uptake, retention, or adherence)****, iv) other outcomes of PrEP use*.”

9) The results section would benefit from greater clarity on how the authors came to these findings. While I recognize there is a very large number of studies being synthesized here, understanding the context and type of study as results are being compared is important. In addition, I was anticipating greater discussion of the contextual and geographic differences, particularly given the global nature of this review and the large number of studies includes.

We thank the reviewer for this comment. We agree that the large number of included studies and their heterogeneity make it challenging to present the context and characteristics of each study alongside every finding without substantially reducing the readability of the Results. We have therefore clarified the study characteristics and geographical distribution in the descriptive results and supplementary tables, and have indicated the type of evidence (quantitative or qualitative) when presenting drivers and barriers. Given the global scope and the heterogeneity of the evidence, we focused the synthesis on common patterns across settings while highlighting important geographical differences where these were apparent. More detailed information on individual studies, including country, UNAIDS region, study design, and outcomes, is provided in the Supplementary tables.

10) The results and discussion talk extensively about PrEP interest and acceptability pre/post PrEP “implementation” or “becoming widely available.” How was this defined? Was this based on how study authors discussed the context of their studies? This needs to be clarified.

While extracting data from the included papers, we assessed whether PrEP was already available in the country at the time of the study. When this information was not reported in the manuscript, we searched online sources to identify the date of the first PrEP programmes in the country.

11) I would urge the authors to be cautious regarding their conclusions on risk compensation. While I agree that there has been limited evidence demonstrating increased sexual risk behaviour and/or bacterial STI acquisition among PrEP users linked to that PrEP use, there continue to be rising rates of bacterial STIs in correlation with rising PrEP usage in many regions and populations.

We thank the reviewer.
We have replaced this sentence: “*They also support the expansion of PrEP programmes without concern for systematic risk compensation among FSWs*.” by “*However, the available evidence remains insufficient to exclude risk compensation in all contexts or to draw conclusions about broader population-level trends in bacterial STI incidence.*” (l.348-349)

12) The discussion would also benefit from a consideration of how these results compare or contrast with findings from PrEP-related reviews among other key populations.

We thank the reviewer for this comment.

We have added a paragraph at the end of the discussion part, before the strengths and limitations (l.352-361):

*“All these findings are broadly consistent with those reported among other key populations, as shown in a recent narrative review [143] identifying facilitators and barriers to oral PrEP uptake at the individual level (e.g. awareness, HIV risk perception, and competing priorities), interpersonal and community levels (e.g. stigma, provider-client interactions, and peer and community support), health system and institutional levels (e.g. opening hours), structural and policy levels (e.g. poverty and economic hardship, gender inequality, and criminalization). However, our findings also highlight barriers that may be particularly relevant to FSWs, including the unpredictability and mobility associated with sex work, concerns about disclosure of sex work, and the need for services adapted to their working conditions. These findings underscore the importance of developing PrEP delivery approaches that are responsive to the specific social and structural contexts of each key population.”*

Typographical:

• UNAIDS should be in all caps

• Table 1: What is “IMRD”?

• Line 67-69, specify this is global average stat

• I recommend reviewing the entire manuscript for flow and readability, particularly given the complexity of the project.

We thank the reviewer.

IMRD was referred to “Introduction, Methods, Results, Discussion”. We have clarified this: *“Reports that did not clearly present the following sections: Introduction, Methods, Results, Discussion”*

We have also made the changed to consider the other comments and for better readability.
