# Supplementary material for: Implementing HIV pre-exposure prophylaxis (PrEP) among cisgender female sex workers: A worldwide scoping review to guide the future research agenda for HIV prevention
Source: PLOS Glob Public Health. 2026 Nov 13;6(11):e0007582. doi: 10.1371/journal.pgph.0007582 (PMC13766235; doi:10.1371/journal.pgph.0007582)
Supplement: Peer review history

# Author comment: Author response to Decision Letter 0

**DOI:** 10.1371/journal.pgph.0007582.r001

**Transfer Alert**

This paper was transferred from another journal. As a result, its full editorial history (including decision letters, peer reviews and author responses) may not be present.

19 May 2026

# Peer review: Decision Letter 0

**DOI:** 10.1371/journal.pgph.0007582.r002

**Authors:** Matthew N Berger

25 Aug 2026

PGPH-D-26-01781

Implementing HIV pre-exposure prophylaxis (PrEP) among female sex workers: a worldwide scoping review to guide the future research agenda for HIV prevention

PLOS Global Public Health

Dear Dr. Plazy,

Thank you for submitting your manuscript to PLOS Global Public Health. After careful consideration, we feel that it has merit but does not fully meet PLOS Global Public Health’s publication criteria as it currently stands. Therefore, we invite you to submit a revised version of the manuscript that addresses the points raised during the review process.

Please submit your revised manuscript by Oct 24 2026 11:59PM. If you will need more time than this to complete your revisions, please reply to this message or contact the journal office at globalpubhealth@plos.org. When you're ready to submit your revision, log on to https://www.editorialmanager.com/pgph/ and select the 'Submissions Needing Revision' folder to locate your manuscript file.

Please include the following items when submitting your revised manuscript:

- A letter that responds to each point raised by the editor and reviewer(s). You should upload this letter as a separate file labeled 'Response to Reviewers'.
- A marked-up copy of your manuscript that highlights changes made to the original version. You should upload this as a separate file labeled 'Revised Manuscript with Track Changes'.
- An unmarked version of your revised paper without tracked changes. You should upload this as a separate file labeled 'Manuscript'.

Guidelines for resubmitting your figure files are available below the reviewer comments at the end of this letter.

As the corresponding author, your ORCID iD is verified in the submission system and will appear in the published article. PLOS supports the use of ORCID, and we encourage all coauthors to register for an ORCID iD and use it as well. Please encourage your coauthors to verify their ORCID iD within the submission system before final acceptance, as unverified ORCID iDs will not appear in the published article. *Only*  the individual author can complete the verification step; PLOS staff *cannot*  verify ORCID iDs on behalf of authors.

We look forward to receiving your revised manuscript.

Kind regards,

Matthew N Berger, PhD

Academic Editor

PLOS Global Public Health

**Journal Requirements:**

1. Please ensure all the standard sections present, (e.g. Abstract, Introduction, Results, Discussion, Methods), in the correct order, and that any references to subheadings in your main text are correct. An outline of the required sections can be consulted in our submission guidelines here:

https://journals.plos.org/globalpublichealth/s/submission-guidelines#loc-parts-of-a-submission.

2. We note that the most recent literature search was completed over a year before the manuscript was submitted to the journal. Systematic and scoping reviews should provide an up-to-date overview of the research topic covered. Therefore, we request that you please update your searches as part of your revision to ensure that the most up-to-date literature is included in your review, or provide scientific justification for not doing so. Please ensure you mention this in your response to reviewers, and discuss whether this has any impact on the Results and Conclusions of the study. If you are submitting a protocol, it is only necessary to state whether the searches will be updated prior to the publication of the results.

3. We note that your Data Availability Statement is currently as follows: All relevant data are within the paper and its Supporting information files.

Please confirm at this time whether or not your submission contains all raw data required to replicate the results of your study. Authors must share the “minimal data set” for their submission. PLOS defines the minimal data set to consist of the data required to replicate all study findings reported in the article, as well as related metadata and methods

https://journals.plos.org/globalpublichealth/s/data-availability#loc-minimal-data-set-definition

For example, authors should submit the following data:

- The values behind the means, standard deviations and other measures reported;

- The values used to build graphs;

- The points extracted from images for analysis.

Authors do not need to submit their entire data set if only a portion of the data was used in the reported study.

If your submission does not contain these data, please either upload them as Supporting Information files or deposit them to a stable, public repository and provide us with the relevant URLs, DOIs, or accession numbers. For a list of recommended repositories, please see

https://journals.plos.org/globalpublichealth/s/recommended-repositories

If there are ethical or legal restrictions on sharing a de-identified data set, please explain them in detail (e.g., data contain potentially sensitive information, data are owned by a third-party organization, etc.) and who has imposed them (e.g., an ethics committee). Please also provide contact information for a data access committee, ethics committee, or other institutional body to which data requests may be sent. If data are owned by a third party, please indicate how others may request data access.

If the reviewer comments include a recommendation to cite specific previously published works, please review and evaluate these publications to determine whether they are relevant and should be cited. There is no requirement to cite these works unless the editor has indicated otherwise.

**Additional Editor Comments (if provided):**

Reviewer 1

Overall, this is a well-written, interesting and timely scoping review that synthesises a large body of literature on PrEP among female sex workers. The topic is highly relevant and the review brings together valuable evidence on PrEP implementation in a population that remains relatively underrepresented in implementation research. The manuscript is generally well organised and will be a useful resource for researchers and programme implementers. I have several comments that I believe would further strengthen the manuscript.

• The manuscript states that an adapted conceptual framework was used to organise the findings, but it is not entirely clear how this framework was adapted, or how it informed the synthesis of the included studies. It would be helpful to explain on what basis this was done.

• The exclusion criteria described in the Abstract appear to differ from those presented in the Methods section. A consistent description would be required.

• The full title indicates that the review aims to "guide the future research agenda". Although research gaps are briefly mentioned in the Discussion, a more elaborate section identifying priority research questions and implementation gaps would better reflect the promise made in the title.

• In several instances, barriers are presented without explaining the underlying mechanisms. For example, unstable housing and alcohol use are listed as barriers to PrEP education (lines 165–166), while alcohol use is also mentioned as a barrier to adherence (lines 179–180). It would strengthen the review to briefly explain the mechanisms reported in the included studies.

• Side effects are repeatedly identified as a barrier to PrEP adherence (e.g. lines 184 and 199–200), but the manuscript does not specify which side effects were most commonly reported. A brief summary would make these findings more informative for readers and programme implementers.

• As expected for a scoping review, no formal quality assessment of the included studies was performed. While this is appropriate, the limitations of drawing conclusions from studies of varying methodological quality could be discussed more explicitly.

• As acknowledged by the authors, the majority of included studies originate from Eastern and Southern Africa. The Discussion could elaborate further on how this geographical concentration may limit the transferability of the findings to other settings where the organisation of sex work, health systems, and HIV prevention programmes differ.

• The Discussion briefly mentions differentiated service delivery models and long-acting PrEP. Given the rapidly evolving field, it would be helpful to briefly explain what is meant by differentiated service delivery and to discuss how newer PrEP modalities may help address some of the barriers identified in this review.

Minor comments

1. Several implementation terms and abbreviations would benefit from being defined when first introduced. For example CAB; the role of a peer navigator.

2. The manuscript uses the term "prostitution sites" (line 225). Unless there is a specific reason to retain the original wording from the included studies, I would recommend using the more commonly accepted term "sex work sites".

3. There are several minor grammatical and typographical errors throughout the manuscript (for example around line 131). A careful language edit would improve readability.

Reviewer 3

Thank you for the opportunity to review this paper. The authors have done an admirable job of synthesizing a broad swatch of research.

However, I have several concerns related to the scope and purpose of this scoping review as well as the methodological approach which limited my ability to assess the results and conclusions drawn by the authors.

1) Throughout abstract, introduction, methods, and discussion, the authors alternate between describing this as a review of PrEP interventions or of PrEP knowledge, interest, uptake, and use more generally around FSWs. This must be clarified, as these are two separate research questions. It appears as though this is about PrEP more broadly, since it does not look like articles had to be about “interventions.”

2) Framing must specify that this is focused on cisgender women engaged in sex work only. This is mentioned briefly, but this should be clearly stated in the introduction and justified in the methods. Terminology should also be clarified (e.g., a statement along the lines of “in this manuscript, we use the terminology “female sex workers” to refer to cisgender women engaged in sex work.”)

3) Introduction would benefit from better clarifying the purpose of this review in particular and how it adds to existing evidence. I recommend engaging with existing systematic reviews such as:

o Glick JL, Russo R, Jivapong B, Rosman L, Pelaez D, Footer KHA, Sherman SG. The PrEP Care Continuum Among Cisgender Women Who Sell Sex and/or Use Drugs Globally: A Systematic Review. AIDS Behav. 2020 May;24(5):1312-1333. doi: 10.1007/s10461-019-02733-z.

o Mpirirwe R, Segawa I, Ojiambo KO, Kamacooko O, Nangendo J, Semitala FC, Kyambadde P, Kalyango JN, Kiragga A, Karamagi C, Katahoire A, Kamya M, Mujugira A. HIV pre-exposure prophylaxis uptake, retention and adherence among female sex workers in sub-Saharan Africa: a systematic review. BMJ Open. 2024 Apr 25;14(4):e076545. doi: 10.1136/bmjopen-2023-076545.

4) The search strategy should be described separately from time frame (lines 96-99) for clarity. Describe search strategy (E.g., MESH terms) and then describe timeframe for published articles. In addition, I have some concerns regarding the search strategy (for example, why were articles required to have “PrEP” AND “HIV” in title or abstract? Were keywords also searched? How were the terms for sex workers chosen?). Was this strategy reviewed by a librarian?

5) Why was “transactional sex” excluded from this review? This is a commonly used term, particularly in research conducted in sub-Saharan Africa. How was this defined and why was this form of sex work excluded?

6) How was data extraction standardized across qualitative and quantitative study types? How were data analyzed? What analyses were conducted?

7) Please better describe the conceptual framework, particularly how it was adapted to PrEP as a preventative medication rather than disease treatment.

8) A definition of a priori and effective PrEP acceptability is needed in the methods section.

9) The results section would benefit from greater clarity on how the authors came to these findings. While I recognize there is a very large number of studies being synthesized here, understanding the context and type of study as results are being compared is important. In addition, I was anticipating greater discussion of the contextual and geographic differences, particularly given the global nature of this review and the large number of studies includes.

10) The results and discussion talk extensively about PrEP interest and acceptability pre/post PrEP “implementation” or “becoming widely available.” How was this defined? Was this based on how study authors discussed the context of their studies? This needs to be clarified.

11) I would urge the authors to be cautious regarding their conclusions on risk compensation. While I agree that there has been limited evidence demonstrating increased sexual risk behaviour and/or bacterial STI acquisition among PrEP users linked to that PrEP use, there continue to be rising rates of bacterial STIs in correlation with rising PrEP usage in many regions and populations.

12) The discussion would also benefit from a consideration of how these results compare or contrast with findings from PrEP-related reviews among other key populations.

Typographical:

• UNAIDS should be in all caps

• Table 1: What is “IMRD”?

• Line 67-69, specify this is global average stat

• I recommend reviewing the entire manuscript for flow and readability, particularly given the complexity of the project.

[Note: HTML markup is below. Please do not edit.]

Reviewers' comments:

Reviewer's Responses to Questions

**Comments to the Author**

1. Does this manuscript meet PLOS Global Public Health’s publication criteria?

Reviewer #1: Yes

Reviewer #2: Yes

Reviewer #3: Partly

**********

2. Has the statistical analysis been performed appropriately and rigorously?

Reviewer #1: N/A

Reviewer #2: Yes

Reviewer #3: I don't know

**********

3. Have the authors made all data underlying the findings in their manuscript fully available (please refer to the Data Availability Statement at the start of the manuscript PDF file)?

The PLOS Data policy

Reviewer #1: Yes

Reviewer #2: Yes

Reviewer #3: Yes

**********

4. Is the manuscript presented in an intelligible fashion and written in standard English?

Reviewer #1: Yes

Reviewer #2: Yes

Reviewer #3: Yes

**********

**Reviewer #1:**  Overall, this is a well-written, interesting and timely scoping review that synthesises a large body of literature on PrEP among female sex workers. The topic is highly relevant and the review brings together valuable evidence on PrEP implementation in a population that remains relatively underrepresented in implementation research. The manuscript is generally well organised and will be a useful resource for researchers and programme implementers. I have several comments that I believe would further strengthen the manuscript.

• The manuscript states that an adapted conceptual framework was used to organise the findings, but it is not entirely clear how this framework was adapted, or how it informed the synthesis of the included studies. It would be helpful to explain on what basis this was done.

• The exclusion criteria described in the Abstract appear to differ from those presented in the Methods section. A consistent description would be required.

• The full title indicates that the review aims to "guide the future research agenda". Although research gaps are briefly mentioned in the Discussion, a more elaborate section identifying priority research questions and implementation gaps would better reflect the promise made in the title.

• In several instances, barriers are presented without explaining the underlying mechanisms. For example, unstable housing and alcohol use are listed as barriers to PrEP education (lines 165–166), while alcohol use is also mentioned as a barrier to adherence (lines 179–180). It would strengthen the review to briefly explain the mechanisms reported in the included studies.

• Side effects are repeatedly identified as a barrier to PrEP adherence (e.g. lines 184 and 199–200), but the manuscript does not specify which side effects were most commonly reported. A brief summary would make these findings more informative for readers and programme implementers.

• As expected for a scoping review, no formal quality assessment of the included studies was performed. While this is appropriate, the limitations of drawing conclusions from studies of varying methodological quality could be discussed more explicitly.

• As acknowledged by the authors, the majority of included studies originate from Eastern and Southern Africa. The Discussion could elaborate further on how this geographical concentration may limit the transferability of the findings to other settings where the organisation of sex work, health systems, and HIV prevention programmes differ.

• The Discussion briefly mentions differentiated service delivery models and long-acting PrEP. Given the rapidly evolving field, it would be helpful to briefly explain what is meant by differentiated service delivery and to discuss how newer PrEP modalities may help address some of the barriers identified in this review.

Minor comments

1. Several implementation terms and abbreviations would benefit from being defined when first introduced. For example CAB; the role of a peer navigator.

2. The manuscript uses the term "prostitution sites" (line 225). Unless there is a specific reason to retain the original wording from the included studies, I would recommend using the more commonly accepted term "sex work sites".

3. There are several minor grammatical and typographical errors throughout the manuscript (for example around line 131). A careful language edit would improve readability.

**Reviewer #2:**  This manuscript portrays the importance of database analysis of PrEP, as a consideration for a better public policy regarding PrEP, which is very valuable for further studies. Even though the included studies mostly from Africa, the manuscript portrays the essential core points of what should be consider to make a better public policy regarding PrEP

**Reviewer #3:**  Thank you for the opportunity to review this paper. The authors have done an admirable job of synthesizing a broad swatch of research.

However, I have several concerns related to the scope and purpose of this scoping review as well as the methodological approach which limited my ability to assess the results and conclusions drawn by the authors.

1) Throughout abstract, introduction, methods, and discussion, the authors alternate between describing this as a review of PrEP interventions or of PrEP knowledge, interest, uptake, and use more generally around FSWs. This must be clarified, as these are two separate research questions. It appears as though this is about PrEP more broadly, since it does not look like articles had to be about “interventions.”

2) Framing must specify that this is focused on cisgender women engaged in sex work only. This is mentioned briefly, but this should be clearly stated in the introduction and justified in the methods. Terminology should also be clarified (e.g., a statement along the lines of “in this manuscript, we use the terminology “female sex workers” to refer to cisgender women engaged in sex work.”)

3) Introduction would benefit from better clarifying the purpose of this review in particular and how it adds to existing evidence. I recommend engaging with existing systematic reviews such as:

o Glick JL, Russo R, Jivapong B, Rosman L, Pelaez D, Footer KHA, Sherman SG. The PrEP Care Continuum Among Cisgender Women Who Sell Sex and/or Use Drugs Globally: A Systematic Review. AIDS Behav. 2020 May;24(5):1312-1333. doi: 10.1007/s10461-019-02733-z.

o Mpirirwe R, Segawa I, Ojiambo KO, Kamacooko O, Nangendo J, Semitala FC, Kyambadde P, Kalyango JN, Kiragga A, Karamagi C, Katahoire A, Kamya M, Mujugira A. HIV pre-exposure prophylaxis uptake, retention and adherence among female sex workers in sub-Saharan Africa: a systematic review. BMJ Open. 2024 Apr 25;14(4):e076545. doi: 10.1136/bmjopen-2023-076545.

4) The search strategy should be described separately from time frame (lines 96-99) for clarity. Describe search strategy (E.g., MESH terms) and then describe timeframe for published articles. In addition, I have some concerns regarding the search strategy (for example, why were articles required to have “PrEP” AND “HIV” in title or abstract? Were keywords also searched? How were the terms for sex workers chosen?). Was this strategy reviewed by a librarian?

5) Why was “transactional sex” excluded from this review? This is a commonly used term, particularly in research conducted in sub-Saharan Africa. How was this defined and why was this form of sex work excluded?

6) How was data extraction standardized across qualitative and quantitative study types? How were data analyzed? What analyses were conducted?

7) Please better describe the conceptual framework, particularly how it was adapted to PrEP as a preventative medication rather than disease treatment.

8) A definition of a priori and effective PrEP acceptability is needed in the methods section.

9) The results section would benefit from greater clarity on how the authors came to these findings. While I recognize there is a very large number of studies being synthesized here, understanding the context and type of study as results are being compared is important. In addition, I was anticipating greater discussion of the contextual and geographic differences, particularly given the global nature of this review and the large number of studies includes.

10) The results and discussion talk extensively about PrEP interest and acceptability pre/post PrEP “implementation” or “becoming widely available.” How was this defined? Was this based on how study authors discussed the context of their studies? This needs to be clarified.

11) I would urge the authors to be cautious regarding their conclusions on risk compensation. While I agree that there has been limited evidence demonstrating increased sexual risk behaviour and/or bacterial STI acquisition among PrEP users linked to that PrEP use, there continue to be rising rates of bacterial STIs in correlation with rising PrEP usage in many regions and populations.

12) The discussion would also benefit from a consideration of how these results compare or contrast with findings from PrEP-related reviews among other key populations.

Typographical:

• UNAIDS should be in all caps

• Table 1: What is “IMRD”?

• Line 67-69, specify this is global average stat

• I recommend reviewing the entire manuscript for flow and readability, particularly given the complexity of the project.

**********

what does this mean?). If published, this will include your full peer review and any attached files.

**Do you want your identity to be public for this peer review?**  If you choose “no”, your identity will remain anonymous but your review may still be made public.

For information about this choice, including consent withdrawal, please see our Privacy Policy

Reviewer #1: **Yes:**  Pam Baatsen

Reviewer #2: No

Reviewer #3: No

**********

[NOTE: If reviewer comments were submitted as an attachment file, they will be attached to this email and accessible via the submission site. Please log into your account, locate the manuscript record, and check for the action link "View Attachments". If this link does not appear, there are no attachment files.]

**Figure Resubmissions:**

# Author comment: Author response to Decision Letter 1

**DOI:** 10.1371/journal.pgph.0007582.r003

23 Sep 2026

Attachment Submitted filename: Response to Reviewers.docx

# Peer review: Decision Letter 1

**DOI:** 10.1371/journal.pgph.0007582.r004

**Authors:** Matthew N Berger

23 Oct 2026

Implementing HIV pre-exposure prophylaxis (PrEP) among cisgender female sex workers: a worldwide scoping review to guide the future research agenda for HIV prevention

PGPH-D-26-01781R1

Dear Mme Plazy,

We are pleased to inform you that your manuscript 'Implementing HIV pre-exposure prophylaxis (PrEP) among cisgender female sex workers: a worldwide scoping review to guide the future research agenda for HIV prevention' has been provisionally accepted for publication in PLOS Global Public Health.

Before your manuscript can be formally accepted you will need to complete some formatting changes, which you will receive in a follow up email. A member of our team will be in touch with a set of requests.

Please note that your manuscript will not be scheduled for publication until you have made the required changes, so a swift response is appreciated.

IMPORTANT: The editorial review process is now complete. PLOS will only permit corrections to spelling, formatting or significant scientific errors from this point onwards. Requests for major changes, or any which affect the scientific understanding of your work, will cause delays to the publication date of your manuscript.

If your institution or institutions have a press office, please notify them about your upcoming paper to help maximize its impact. If they'll be preparing press materials, please inform our press team as soon as possible -- no later than 48 hours after receiving the formal acceptance. Your manuscript will remain under strict press embargo until 2 pm Eastern Time on the date of publication. For more information, please contact globalpubhealth@plos.org.

Thank you again for supporting Open Access publishing; we are looking forward to publishing your work in PLOS Global Public Health.

Best regards,

Matthew N Berger, PhD

Academic Editor

PLOS Global Public Health

***********************************************************

Reviewer Comments (if any, and for reference):

Reviewer's Responses to Questions

**Comments to the Author**

Reviewer #1: All comments have been addressed

Reviewer #3: All comments have been addressed

**********

publication criteria?

Reviewer #1: Yes

Reviewer #3: Yes

**********

3. Has the statistical analysis been performed appropriately and rigorously?

Reviewer #1: N/A

Reviewer #3: N/A

**********

4. Have the authors made all data underlying the findings in their manuscript fully available (please refer to the Data Availability Statement at the start of the manuscript PDF file)?

The PLOS Data policy

Reviewer #1: Yes

Reviewer #3: Yes

**********

5. Is the manuscript presented in an intelligible fashion and written in standard English?

Reviewer #1: Yes

Reviewer #3: Yes

**********

Reviewer #1: I have no further comments on the article as the authors have successfully addressed all my earlier comments.

Reviewer #3: (No Response)

**********

what does this mean?). If published, this will include your full peer review and any attached files.

**Do you want your identity to be public for this peer review?**  If you choose “no”, your identity will remain anonymous but your review may still be made public.

For information about this choice, including consent withdrawal, please see our Privacy Policy

Reviewer #1: **Yes:**  Pam Baatsen

Reviewer #3: No

**********
